# An integrated lifestyle intervention for mitigation of adverse effects associated with androgen deprivation therapy for prostate cancer (STAMINA): a multicentre, randomised trial

**DOI:** 10.1016/S1470-2045(26)00304-9

**Published:** 2026-10

**Authors:** Liam Bourke, Derek J Rosario, Sophie Reale, Said Ibeggazene, Aidan Innes, Alison Scope, Grace Price, Steph Taylor, Liz Steed, Eileen Sutton, Jamie Stokes, Jenny Hewison, Dylan Morrissey, Patrick Doherty, Janet Brown, David Meads, John L O’Dwyer, Davina Deniszczyc, Diana M Greenfield, Malcolm Mason, Michelle Collinson, Amanda Farrin, STAMINA investigators, Amit Bahl, Julia Brown, Claire Brotherton, Michelle Cain, Noel Clarke, Philip Cornford, Marcus Cumberbatch, Kim Davenport, Florence Day, James Dyer, Abi Fisher, Chee Goh, Bea Hamilton, Suzanne Hartley, Claire Hulme, Kieran Jefferson, Ben Kelly, Kate Linton, Ellen Mason, Emma McNaught, Chidi Molokwu, Nanette Muttrie, Raj Persad, Tim Porter, Kasra Saeb-Parsy, Matt Simms, Joe Sullivan, Chris Taylor, Rebecca Turner, Simon Williams, Geoffrey Wong

**Affiliations:** a School of Health and Social Care, CARe Research Centre, Sheffield Hallam University, Sheffield, UK; b Sheffield Teaching Hospitals NHS Trust, Sheffield, UK; c Nuffield Health, Epsom, Surrey, UK; d Wolfson Institute of Population Health, Queen Mary University of London, London, UK; e University of Bristol, Bristol, UK; f Leeds Institute of Clinical Trials Research, University of Leeds, Leeds, UK; g Leeds Institute of Health Sciences, University of Leeds, Leeds, UK; h Sports and Exercise Medicine, Queen Mary University of London, London, UK; i Barts Health NHS Trust, London, UK; j Department of Health Sciences, University of York, York, UK; k Division of Clinical Medicine, University of Sheffield, Sheffield, UK; l Cardiff University, Cardiff, UK; m Edinburgh Clinical Trials Unit, University of Edinburgh, Edinburgh, UK

## Abstract

**Background:**

Androgen deprivation therapy (ADT) for prostate cancer causes substantial adverse effects. Despite consistent national and international guideline recommendations, supervised exercise is rarely integrated into care. We aimed to determine whether the STAMINA lifestyle intervention, embedded into cancer care, would improve cancer-specific quality of life and fatigue versus behaviourally Optimised Usual Care in men with prostate cancer in England.

**Methods:**

STAMINA was a multicentre, randomised trial done across 15 UK National Health Service (NHS) trusts in England. Men on ADT for prostate cancer were eligible. Participants were randomly assigned (5:4) via computer-generated minimisation, stratified by age, ADT duration, chemotherapy or androgen receptor pathway inhibitor therapy, and radiotherapy, to the STAMINA lifestyle intervention or to Optimised Usual Care. Participants were aware of treatment allocation. The STAMINA lifestyle intervention comprised supervised aerobic and resistance exercise for 12 months, dietary advice, behavioural support, and complimentary gym membership. Optimised Usual Care comprised clinician training, educational materials, behavioural prompts, and safety-to-exercise checks. Primary outcomes were the Functional Assessment of Cancer Therapy–prostate (FACT-P) and the Functional Assessment of Chronic Illness Therapy–fatigue (FACIT-F) subscale at 12 months, analysed by intention to treat (according to randomised treatment). This trial is registered with ISRCTN (ISRCTN46385239), and recruitment is complete.

**Findings:**

Between Jan 20, 2022, and June 12, 2023, 700 men were randomly assigned to STAMINA lifestyle intervention (n=389) or Optimised Usual Care (n=311). Median age was 71·6 years (IQR 66·3–76·0), and 680 (97%) of 700 participants were White. Primary outcome data were available for 345 (89%) of 389 participants in the STAMINA lifestyle intervention group and 251 (81%) of 311 in the Optimised Usual Care group. The STAMINA lifestyle intervention was superior to Optimised Usual Care in terms of FACT-P score (adjusted mean difference 4·5, 97·232% CI 1·7–7·2; p=0·0004) and FACIT-F score (1·9, 0·4–3·4; p=0·0068). Three intervention-related serious adverse events occurred in the STAMINA lifestyle intervention group (transient loss of consciousness, leg pain or weakness, and back pain); all participants recovered. No treatment-related deaths occurred.

**Interpretation:**

The STAMINA lifestyle intervention meets best practice guidelines, can be implemented in NHS trusts in England, and offers clinicians a clear basis for identification and prescription of a supervised exercise and dietary advice intervention to mitigate negative effects associated with ADT.

**Funding:**

National Institute for Health Research.

## Introduction

The incidence and prevalence of prostate cancer are rising but so too is survival.^1^ As such, there is an ever-expanding survivorship population living with the adverse effects of treatment, reflected in an accelerating rate of prostate cancer-related disability-adjusted life years internationally.^2^ As prostate cancer survival improves, mitigating the toxicity of treatments and improving quality of life has been declared of strategic priority in the UK^3^ and the USA.^4^ Medical castration (referred to as androgen deprivation therapy [ADT]), either in isolation for metastatic disease or in combination with other modalities such as radiotherapy, androgen receptor pathway inhibitors, or chemotherapy, is prescribed at some point in around 50% of men with prostate cancer and has a documented negative impact on cancer-specific quality of life, particularly associated with fatigue in up to 77% of men treated.^5^

Evidence from small trials of limited follow-up^6^ suggests supervised exercise training as a strategy to mitigate ADT-associated problems. It is noteworthy that restricting men on ADT from physical activity has been reported to result in profoundly worse cancer-specific quality of life and fatigue over 12 months (28% and 33% deterioration from baseline, respectively) compared with men who are exercising.^7^ Providing supervised exercise training programmes for men on ADT is consistently featured in national (the National Institute for Health and Care Excellence [NICE]) and international (European Association of Urology [EAU]) prostate cancer guidelines, but is rarely delivered in practice.^8^

**Research in context**

**Evidence before this study**

Men undergoing medical castration (known as androgen deprivation therapy [ADT]) experience profound negative impacts on their quality of life and worsening fatigue. Exercise interventions can have important clinically relevant benefits for people with cancer, but only if they can be sustained. However, very few providers within the health-care system offer any such interventions (even short-term) to mitigate these problems, despite national and international guidelines consistently highlighting this as best practice. Inherent limitations within the evidence base, a lack of bespoke professional training, uncertainty over how to make exercise safe, as well as missing crucial infrastructure for referral and delivery are major driving factors behind this. This also makes large, multicentre clinical-effectiveness and cost-effectiveness trials very difficult to undertake successfully at scale.

We updated findings from our 2016 systematic review to look for randomised clinical trials (RCTs) assessing cancer-specific quality of life or fatigue in men on ADT with at least 12 months of follow-up using the following criteria. We searched MEDLINE (via EBSCO, searching Medline, Sports Discus, CINAHL databases) on Sept 26, 2025 using search terms “exercise or physical* or activ* or resistance training* or weight training or lifestyle* AND prostate cancer or prostatic neoplasms or prostate carcinoma”, applying search date range 2016–25, and restricting search to: human participants only, RCTs only involving men on ADT, and evaluating cancer-specific quality of life or fatigue using previously validated patient-reported outcome measures. This resulted in a further 197 articles identified at title stage, 23 abstracts, and five new relevant trials included in the discussion after full-text review.

**Added value of this study**

In the largest study of its kind to date, to our knowledge, the STAMINA lifestyle intervention provided superior mitigation effects on the Functional Assessment of Cancer Therapy–prostate (FACT-P), the Functional Assessment of Chronic Illness Therapy–fatigue, FACT–general (FACT-G), and the EuroQol five dimensions, five levels outcomes at 12 months, over a behaviourally Optimised Usual Care as well as consistently superior exercise behaviour. STAMINA lifestyle intervention provided cancer-specific quality-of-life benefits, which exceeded the minimum clinically important difference threshold (ie, >3 points on FACT-G) and importantly provided positive long-term effects across subdomains. STAMINA lifestyle intervention is cost-effective compared with Optimised Usual Care, being deliverable for £13 920 per quality-adjusted life years and costing less than £1000 per annum even under the more expensive scenario examined. A unique feature of STAMINA lifestyle intervention was the integration of the lifestyle prescription into the standard prostate cancer clinical pathway for men being prescribed ADT. Partnering with Nuffield Health, who delivered supervised exercise via their national infrastructure, ensured that if demonstrated to be positive, STAMINA lifestyle intervention could be implemented at scale immediately. These data directly address lingering uncertainties around the quality of evidence and the absence of core delivery infrastructure underpinning the utility of these interventions.

**Implications of all the available evidence**

The importance of quality of life in cancer survivors should not be underestimated. A recent meta-analysis of trials showed that better quality of life and lower fatigue outcomes do translate to better overall survival for patients with cancer. Clinicians now have a robust evidence base for offering lifestyle interventions for men on ADT and a fully developed identification, referral, and delivery network across the country. While this intervention can be implemented in the National Health System in England, based on published attempts to date, the STAMINA design serves also as the most widely generalisable basis for delivery of a supervised exercise and dietary advice intervention to mitigate negative effects associated with ADT. It is a reasonable hypothesis that the STAMINA model could translate well to other cancer groups that would also benefit from such programmes—eg, breast cancer and bowel cancer.

Previous work has shown the willingness of clinicians to be involved in delivering guideline-recommended exercise programmes; however, scepticism exists around the quality of evidence and the absence of quality-assured behavioural support. Additionally, the embedded infrastructure for delivery of structured exercise programmes in clinical populations does not exist in the UK^8^ or North America, as asserted in a recent editorial on the CHALLENGE trial.^9^ The inability to recruit participants for the recent INTERVAL-GAP-4 trial, which compared a combined supervised and self-managed exercise programme with self-directed exercise in men with metastatic prostate cancer, emphasised this problem.^10^ An integrated clinical pathway with reliable large-scale infrastructure for delivery is essential to the design of such therapeutic strategies if they are to succeed.

We hypothesised that a long-term lifestyle prescription consisting of supervised exercise training with dietary advice and behavioural support for men on ADT could be integrated into core-National Health Service (NHS) prostate cancer care. Following initial prescription by the treating clinical team, the STAMINA lifestyle intervention would be delivered in a community setting by upskilled personal trainers. In this randomised controlled trial (RCT), we tested the effects of the STAMINA lifestyle intervention on cancer-specific quality of life and fatigue, along with adverse effects and cost-effectiveness. The STAMINA lifestyle intervention was compared with an active comparator—a behaviourally optimised version of the usual clinical care pathway termed Optimised Usual Care—in accordance with existing clinical guidelines recommending supervised exercise for men on ADT.

## Methods

### Study design and participants

This was a multicentre, randomised superiority trial of STAMINA lifestyle intervention versus Optimised Usual Care with a two-level partially nested design. The trial protocol has previously been published^11^ and subsequent amendments (appendix pp 1–4) were approved by West of Scotland Research Ethics Committee (1 20/WS/0069). No amendments to eligibility criteria, primary outcomes, or analysis plans were made. The trial was conducted in accordance with the principles of Good Clinical Practice, the Declaration of Helsinki. The University of Leeds independent Clinical Trials Research Unit ensured trial monitoring and outcome data integrity*.*

Our patient and public involvement group was involved extensively throughout design, conduct, documentation, oversight, and outputs for the STAMINA Programme Development and full Programme Grant. Development work incorporating these data have been published.^11^ Patient and public involvement input was key in selecting our primary outcomes, and the panel met with the investigators at regular intervals during the 7 years of the programme. During design meetings, patient and public involvement representatives felt strongly that a no-intervention control was not justifiable in the STAMINA RCT due to extant clinical guidelines, data from our development work and ethics committee stipulations.

Participants were recruited from NHS secondary care centres across across 15 UK National Health Service (NHS) trusts in England (appendix p 27). Men already receiving ADT, or due to commence ADT over the ensuing 12 weeks, for prostate cancer were eligible for inclusion. Individuals were excluded if they were previously involved in pre-pilot work, were participating in concurrent lifestyle intervention trials, had a life expectancy of less than 12 months, had absolute contraindications to exercise participation as per guidelines, or were unable to provide consent or complete study assessments. After initial counselling by their NHS clinical team, including verbal consent, participants were referred to the study researchers for full eligibility screening, informed consent for trial participation, and collection of baseline data over the telephone before random allocation. Ethnicity data were self-reported by participants. Sex data were not collected. This trial is registered with ISRCTN, ISRCTN46385239, and recruitment is complete.

### Randomisation and masking

Trial researchers enrolled participants and conducted randomisation. To account for potential clustering at the level of the clinical exercise specialist in the STAMINA lifestyle intervention group, participants were randomly assigned (5:4) to either STAMINA lifestyle intervention or Optimised Usual Care via an online randomisation system at the University of Leeds Clinical Trials Research Unit to ensure allocation concealment, stratified by age (<70 years *vs* ≥70 years), duration on ADT (≤12 weeks *vs* >12 weeks), receipt of chemotherapy or novel androgen receptor pathway inhibitors (yes *vs* no), and receipt of radiotherapy (yes *vs* no).^11^ A computer-generated minimisation programme incorporating a random element (allocation to minimised group probability p=0·8) allocated participants and ensured groups were well balanced for strata. Participants, NHS staff, and Nuffield Health staff were aware of treatment allocation, but researchers involved in data collection, general practitioners, and research nurses were not informed of allocation. Trial researchers who analysed the data were unblinded but not involved in outcome data collection.

### Procedures

For Optimised Usual Care, clinical staff treating prostate cancer in participating urology and oncology departments received bespoke training to recognise men on ADT who were eligible for STAMINA, recommend exercise training as part of NICE guidelines, refer men to the research team, and record their participation in the medical notes. Training included education around (NICE and EAU) lifestyle recommendations to support men on ADT in making and maintaining required behavioural changes during clinical follow-up. Participants were sent publicly available information educational booklets. All patients received behavioural prompts and monitoring from their clinical care team at routine clinic visits. Following referral, a safe-to-exercise check was carried out by a trial researcher remotely.

In addition to receiving Optimised Usual Care, men assigned to STAMINA lifestyle intervention received a supervised exercise programme including both aerobic and resistance components^12^ with dietary advice in Nuffield Health fitness and wellbeing centres.^11^ All clinical exercise specialists were upskilled experienced community personal trainers. Supervised exercise sessions were tailored to individual capability delivered 1:1 before transition to group delivery (maximum group size of five participants). Supervision was carried out twice weekly for 13 weeks, then negotiated between the participant and clinical exercise specialists (maximum once a month, minimum once every 3 months) over the subsequent 9 months (12 months in total). Complimentary 12-month gym membership was provided. Further details on both interventions are provided in the appendix (pp 5–6).

Participants could withdraw from any trial procedure at any time. Cessation of STAMINA lifestyle intervention delivery was at participant or clinical team discretion. Data collection continued for all participants unless consent for further follow-up data collection was withdrawn. Adverse events including related and unexpected serious adverse events and the date and cause of all deaths occurring after randomisation were gathered by researcher, NHS staff, or Nuffield Health staff via contact during intervention and at follow-up timepoints.

### Outcomes

Primary outcomes were cancer-specific quality of life and fatigue at 12-month post-randomisation as measured by FACT-P^13^ and FACIT-F fatigue subscale^14^ questionnaires, respectively. Outcome data were collected at 3, 6, and 12 months. Analyses includes a parallel mixed-methods process evaluation (reported separately). Key secondary endpoints measured at months 3, 6, and 12 post-randomisation were FACT-P subscales (prostate cancer, physical, social and family, emotional, and function wellbeing), FACT–general (FACT-G) total score, independent physical activity via Godin questionnaire,^15^ fear of recurrence (FCR4) and psychological distress (FCR7).^16^ General health-related quality of life was assessed using the EuroQol five dimensions, five levels (EQ-5D-5L) instrument, capturing health status across mobility, self-care, usual activities, pain or discomfort, and anxiety or depression.^17^ Responses were mapped to the EQ-5D-3L index by recommended algorithm.^18^

Physical outcomes included functional capacity (chair sit-to-stand), body composition, (waist and hip circumference and weight) and systolic and diastolic blood pressure (measured by nursing team at site). COM-B model questionnaires and a purpose-designed questionnaire assessing the adverse effects of ADT will be reported elsewhere as secondary analyses.

Pre-randomisation baseline data (FACT-P, FACIT-F, and functional secondary subscores) were collected by study researchers. Other baseline assessments were collected by participant self-report via postal or online questionnaires. Post-randomisation outcome questionnaires were collected by participant self-report via post or online. Non-responders were sent up to three reminders by post, phone, email, or text according to preference. Supported completion of questionnaires by site staff was available on request.

### Statistical analysis

In the absence of available trials evaluating two active interventions similar to STAMINA lifestyle intervention and Optimised Usual Care, for sample size calculation, we used data reported in our phase 3, single-centre study comparing a lifestyle intervention with a no-intervention control group.^12^ Allowing for a correlation between the two primary endpoints^19^ FACT-P and FACIT-F of 0·58, a maximum SD of 24 (FACT-P) and 8·6 (FACIT-F) and a priori assumed 30% loss to follow-up at 12 months, a total sample size of 697 men (313 in Optimised Usual Care and 384 in STAMINA lifestyle intervention) was deemed necessary to provide 90% power to detect an effect size of either 0·33 in FACT-P (8-point difference) or 0·35 in FACIT-F (3-point difference) at the 5% significance level. The adjusted α level was set at 0·02768 to account for multiple testing.^19^

In men with prostate cancer on ADT, or likely to start ADT within 3 months, the estimand is the difference in mean FACT-P total score or FACIT-F subscale score between STAMINA lifestyle intervention and Optimised Usual Care at 12 months from randomisation or death (whichever occurs first), regardless of withdrawal, deviations from the protocol or adherence to the intervention.

A single final analysis of outcomes, including internal pilot data, was conducted according to a prespecified analysis plan. Primary and secondary analyses were done in the intention-to-treat population with participants grouped and analysed according to randomised treatment, regardless of compliance with or withdrawal from the trial. Missing data were imputed via a multiple imputation strategy using a pattern mixture modelling framework to allow for departures from the missing at random assumption, allowing for anticipated differential dropout between trial groups (appendix pp 7–9). Participants were only excluded from analyses if they died before contributing the relevant outcome. We compared outcomes between groups using a generalised linear mixed-effects multivariable regression model adjusting for minimisation strata (with age treated as continuous), baseline score of the dependent variable and site as fixed effects. We originally planned to adjust for clinical exercise specialists’ level clustering as a random effect in the primary analyses using an intracluster correlation coefficient (ICC) of 0·05 and a coefficient of variation of 0·6, but average cluster sizes were too small (<5) for this to be practical. Results were expressed as adjusted mean differences (STAMINA lifestyle intervention – Optimised Usual Care) with 97·232% CI and p values. A exploratory complier average causal effect analysis explored the impact of compliance with STAMINA lifestyle intervention on the primary outcomes. Exploratory moderator analyses investigated whether the treatment effect varied by participant baseline characteristics using a treatment-moderator interaction in separate analyses at 12-months post-randomisation. Causal mediation analysis explored the mechanism of action of STAMINA lifestyle intervention on the primary outcomes. Prespecified analyses were performed in SAS version 9.4, Stata version 18.0 or 19.0, or R version 4.3.1. Further details of the statistical methods are presented in the appendix (pp 7–9).

The economic evaluation was a cost-utility analysis conducted alongside the trial, over a 12-month time horizon, using a modified NHS and Personal Social Services perspective. Cost data were collected at baseline and months 3, 6, and 12 using a resource use questionnaire covering primary and secondary health-care contacts, patient and carer expenses, and intervention-related costs. Resource use was valued using national unit costs, including the NHS Reference Costs and the Personal Social Services Research Unit. Intervention delivery costs included facilitator time for training delivery, NHS, and Nuffield Health staff time for training receipt, ongoing support, safety-to-exercise assessments and supervised training sessions (appendix pp 10–12). We applied gym membership cost scenarios (£50 in scenario 1 and £35 a month in scenario 2) although gym memberships were complementary in the trial. We also included other gym and exercise-related costs incurred by men. We estimated quality-adjusted life years (QALYs) over 12 months using an area under the curve approach, with utility based on the EQ-5D-5L. We estimated incremental cost-effectiveness ratios (ICERs) as cost per QALY gained, using seemingly unrelated regression models to jointly estimate costs and outcomes, adjusting for baseline covariates, stratification factors, and site. We used a cost per QALY threshold of £25 000 to interpret the ICERs. Our economic analysis used pattern mixture modelling multiple imputation, with sensitivity analyses considering a complete case analytical sample and target exercise session group size (n=5). We used resampling techniques to understand the uncertainty in the analysis and to generate the probability of cost-effectiveness, represented in cost-effectiveness acceptability curves (appendix p 23). We conducted a post-hoc supplementary analysis using a decision model to extrapolate the cost-effectiveness outcomes over a 10-year time horizon (appendix pp 24–26). All analyses were conducted in Stata (version 18.0).

### Role of the funding source

The funder of the study had no role in study design, data collection, data analyses, data interpretation, or writing of the report.

## Results

Of 2094 patients screened at 15 hospitals between Jan 20, 2022, and June 12, 2023, 1656 were referred and deemed eligible for the trial, of whom 752 were registered and provided consent. 52 participants were excluded after registration, mostly due to loss of interest and changes in eligibility, and 700 were randomly assigned: 389 (56%) to STAMINA lifestyle intervention and 311 (44%) to Optimised Usual Care (figure). Median age was 71·6 years (IQR 66·3–76·0), and 680 (97%) of 700 participants were White (table 1). Baseline characteristics were similar between groups (table 1); almost three-quarters reported two or more comorbidities (513 [73%] of 700), most commonly hypertension, musculoskeletal complaints, and arthritis, (appendix pp 13–14). More than half of participants had been on ADT for 12 weeks or less (397 [57%] of 700), 239 (34%) had metastatic disease and 136 (19%) were receiving androgen receptor pathway inhibitors or chemotherapy. Follow-up ended in June, 2024.

**Figure:**
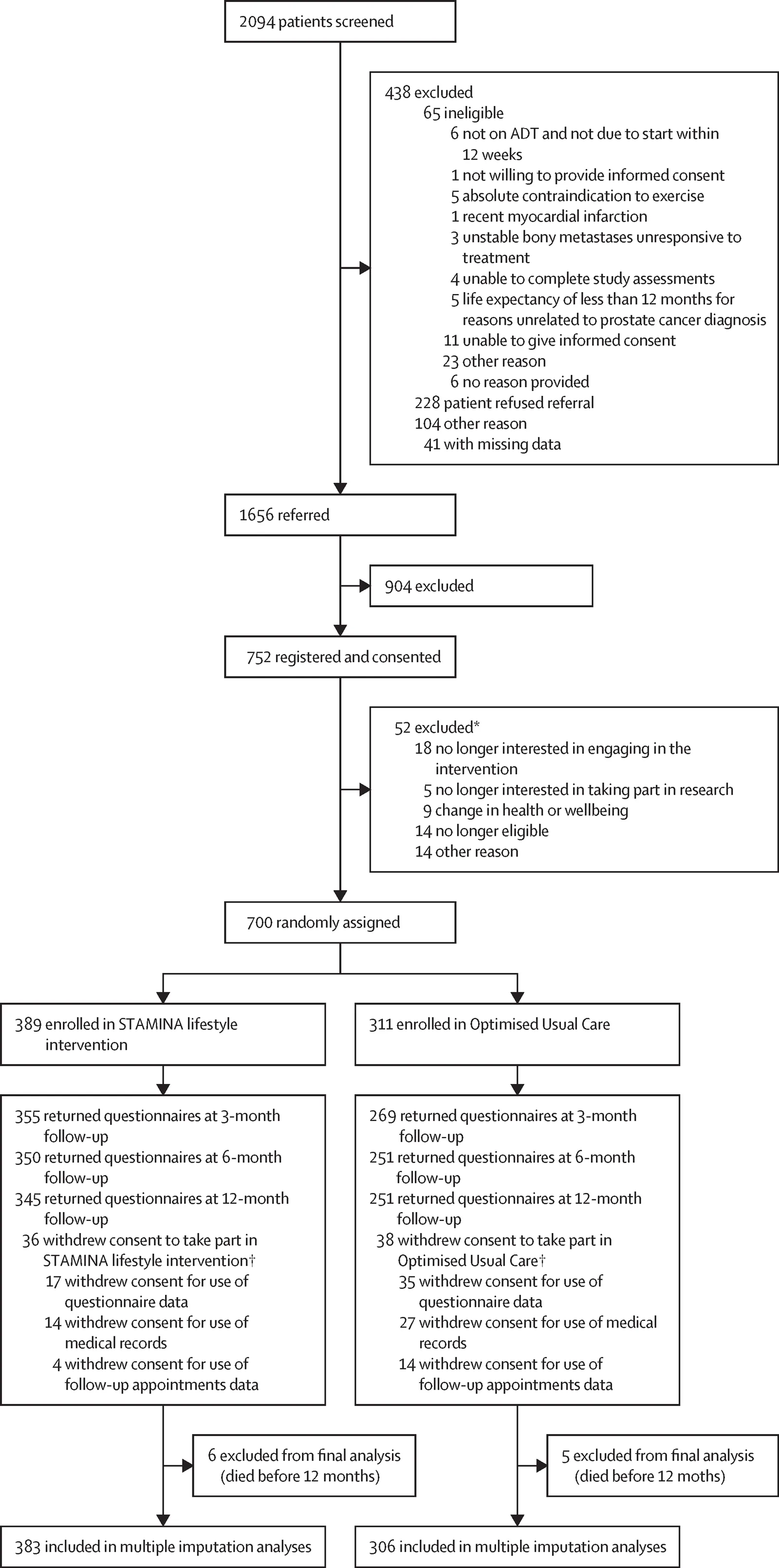
CONSORT flow diagram of trial recruitment, follow-up, and analysis ADT=androgen deprivation therapy. *Patients could be excluded for more than one reason. †Patients could withdraw consent for more than one reason.

**Table 1:** Baseline characteristics

		**STAMINA lifestyle intervention group (n=389)**	**Optimised usual care group(n=311)**
**Participant characteristics**			
Age, years		71·4 (50·0–86·5)	71·9 (48·5–88·9)
Ethnicity			
	White	377 (97%)	303 (97%)
	Black	3 (1%)	6 (2%)
	Asian	5 (1%)	2 (1%)
	Mixed	3 (1%)	0
	Other	1 (<1%)	0
IMD decile			
	1 (most deprived)	13 (3%)	13 (4%)
	2	18 (5%)	10 (3%)
	3	23 (6%)	18 (6%)
	4	31 (8%)	25 (8%)
	5	34 (9%)	25 (8%)
	6	35 (9%)	35 (11%)
	7	45 (12%)	34 (11%)
	8	63 (16%)	56 (18%)
	9	56 (14%)	49 (16%)
	10 (least deprived)	69 (18%)	46 (15%)
	Missing	2	0
Eastern Cooperative Oncology Group performance status			
	0	191 (49%)	145 (47%)
	1	38 (10%)	43 (14%)
	2	3 (1%)	2 (1%)
	Missing	157 (40%)	121 (39%)
Current health (self-rated)			
	Very poor health	1 (<1%)	1 (<1%)
	Unhealthy	14 (4%)	19 (6%)
	Moderately healthy	131 (34%)	93 (30%)
	Relatively good health	243 (62%)	198 (64%)
Current level of self-rated fitness			
	Unfit	70 (18%)	68 (22%)
	Moderately fit	278 (71%)	215 (69%)
	Very fit (already in structured exercise training)	41 (11%)	28 (9%)
Regularly doing more than 90 min of moderate intensive exercise per week: yes		170 (44%)	132 (42%)
Number of comorbidities			
	0	25 (6%)	19 (6%)
	1	70 (18%)	73 (23%)
	≥2	294 (76%)	219 (70%)
Disease status			
	Localised disease (T1–2 N0 M0)	92 (24%)	74 (24%)
	Locally advanced (T3–4 N0 M0, Tx N1 M0)	157 (40%)	119 (38%)
	Hormone sensitive	110 (28%)	91 (29%)
	Castrate resistant	21 (5%)	17 (5%)
	Missing	9 (2%)	10 (3%)
Type of androgen deprivation therapy received			
	Agonist	240 (62%)	184 (59%)
	Maximum androgen blockade	109 (28%)	90 (29%)
	Antagonist	35 (9%)	33 (11%)
	Unknown	5 (1%)	4 (1%)
Planned radiotherapy to treat prostate cancer in next 3 months			
	Yes	161 (41%)	121 (39%)
	No	227 (58%)	190 (61%)
	Missing	1 (<1%)	0
**Stratification factors**			
Age			
	<70 years	161 (41%)	128 (41%)
	≥70 years	228 (59%)	183 (59%)
Duration on androgen deprivation therapy			
	≤12 weeks	221 (57%)	176 (57%)
	>12 weeks	168 (43%)	135 (43%)
Receiving chemotherapy or novel androgen receptor pathway inhibitors, or both			
	Yes	74 (19%)	62 (20%)
	No	315 (81%)	249 (80%)
Receiving radiotherapy			
	Yes	10 (3%)	7 (2%)
	No	379 (97%)	304 (98%)

Data are n (%) or median (IQR). Data on ethnicity, postcode (used to determine IMD); current health; current level of fitness; current exercise level; comorbidities; future radiotherapy treatment; receiving chemotherapy or novel androgen receptor pathway inhibitor, or both; and receiving radiotherapy were self-reported by the participant to the trial researcher. Data on age, disease status, and type of androgen deprivation therapy received were collected from the clinical record. Data on Eastern Cooperative Oncology Group performance status was clinician assessed. Data on duration on androgen deprivation therapy were self-reported by the participant to the trial researcher and verified where possible using the clinical record. IMD=Index of Multiple Deprivation: 1=neighbourhood in the 10% most deprived neighbourhoods in England, 2=20%, 3=30%, 4=40%, 5=50%, 6=60%, 7=70%, 8=80%, 9=90%, 10=neighbourhood in the 10% least deprived neighbourhoods in England.

A total of 74 participants (11%) withdrew consent from at least one trial intervention (figure). The most common reasons for withdrawal were participants changing their mind about being involved in the trial (22 [23%]), the participant was too unwell to continue in the trial (22 [23%]), and participants withdrawing as they had a preference for the STAMINA lifestyle intervention and were randomised to Optimised Usual Care (ten [10%]).

Training in Optimised Usual Care was provided to 183 hospital-based staff (136 core clinical staff from urology and oncology, 44 research staff, and three clinical support staff) from 16 NHS hospitals. STAMINA lifestyle intervention training was delivered to 157 gym-based staff (one general manager, 15 fitness managers, and 141 exercise professionals) at 18 Nuffield Health fitness and wellbeing centres.

At least one supervised exercise session was delivered to 369 (95%) of 389 STAMINA lifestyle intervention participants, with the first session occurring within 1 month of randomisation (median 26 days, IQR 20–36). The median number of sessions attended was 28 (IQR 24–31; appendix p 15). Aerobic exercise was performed in 9522 (99%) of 9580 attended sessions and resistance exercise was performed in 9287 (97%) of 9580 sessions.

The primary analysis included 383 (98%) of 389 patients in the STAMINA lifestyle intervention group and 306 (98%) of 311 patients in the Optimised Usual Care group, respectively; 11 participants (six in the STAMINA lifestyle intervention group and five in the Optimised Usual Care group) died before the 12-month timepoint and were excluded from the primary outcome analysis (figure). 345 (89%) of 389 participants in the STAMINA lifestyle intervention group and 251 (81%) of 311 participants in the Optimised Usual Care group returned questionnaires at 12 months; primary outcome data for other participants were handled by multiple imputation. STAMINA lifestyle intervention was superior to Optimised Usual Care in offsetting deterioration in FACT-P (adjusted mean difference 4·5, 97·232% CI 1·7 to 7·2, p=0·0004) and FACIT-F (adjusted mean difference 1·9, 0·4 to 3·4, p=0·0068) over the 12 months of intervention (table 2). These findings were consistent across earlier follow-up timepoints. Treatment effects were similar to the main analysis when assessed by complete-case analysis, after applying clustering according to the Nuffield Health centre attended, and in a exploratory complier average causal effect analysis at 12 months (appendix pp 16–17). In the causal effect analysis, a non-statistically significant between-group difference of 4·5 points in was found for exercise compliers (p=0·039, appendix p 17).

**Table 2:** Primary and secondary endpoint data

			**Baseline**		**3 months**			**6 months**			12 months*		
			STAMINA lifestyle intervention group	Optimised usual care group	STAMINA lifestyle intervention group	Optimised usual care group	Treatment effect (97·232% CI), p value	STAMINA lifestyle intervention group	Optimised usual care group	Treatment effect (97·232% CI), p value	STAMINA lifestyle intervention group	Optimised usual care group	Treatment effect (97·232% CI), p value
Questionnaire pack completed†			389/389 (100%)	311/311 (100%)	355/389 (91%)	269/311 (86%)	..	350/389 (90%)	251/311 (81%)	..	345/389 (89%)	251/311 (81%)	..
Primary endpoints													
	FACT-P		124·9 (15·8)	123·9 (17·0)	118·9 (17·0)	114·6 (20·1)	3·7 (1·4 to 6.0); p=0·0004	117·9 (18·1)	115·2 (20·1)	3·2 (0·5 to 5·8); p=0·0080	118·3 (19·1)	115·0 (20·0)	4·5 (1·7 to 7·2); p=0·0004
	FACIT-F		42·6 (8·3)	42·2 (8·9)	39·8 (9·5)	37·5 (10·6)	2·1 (0·8 to 3·4); p=0·0004	38·7 (9·9)	37·3 (10·7)	1·5 (0·0 to 3·0); p=0·024	39·1 (10·2)	37·2 (10·5)	1·9 (0·4 to 3·4); p=0·0068
Secondary endpoints													
	FACT-P subscales												
		Physical wellbeing	24·4 (3·1)	24·1 (3·5)	23·9 (3·7)	22·4 (4·9)	1·2 (0·6 to 1·8); p<0·0001	23·5 (3·7)	22·7 (4·5)	0·8 (0·1 to 1·4); p=0·0096	23·7 (3·9)	22·6 (4·3)	1·2 (0·5 to 1·9); p<0·0001
		Social and family wellbeing	22·7 (3·9)	22·5 (4·2)	21·6 (5·1)	21·4 (4·9)	0·1 (−0·6 to 0·8); p=0·67	21·7 (4·7)	21·1 (5·2)	0·7 (0·0 to 1·5); p=0·033	21·4 (5·2)	20·6 (5·3)	0·8 (0·0 to 1·7); p=0·024
		Emotional wellbeing	19·4 (3·6)	19·5 (3·7)	19·3 (3·5)	19·2 (3·9)	0·3 (−0·2 to 0·8); p=0·22	19·2 (3·7)	19·2 (4·0)	0·3 (−0·3 to 0·9); p=0·24	19·3 (3·7)	19·0 (4·2)	0·7 (0·1 to 1·3); p=0·014
		Functional wellbeing	22·7 (4·6)	22·5 (4·8)	20·6 (5·1)	19·4 (5·8)	1·2 (0·5 to 1·9); p=0·0002	20·3 (5·3)	19·8 (5·7)	0·8 (0·0 to 1·6); p=0·023	20·6 (5·5)	20·0 (5·9)	1·0 (0·1 to 1·8); p=0·012
		Prostate cancer	35·8 (5·9)	35·2 (6·4)	33·2 (6·6)	32·0 (6·8)	1·1 (0·1 to 2·0); p=0·012	32·7 (6·8)	32·2 (6·8)	0·4 (−0·6 to 1·5); p=0·38	33·2 (6·9)	32·3 (6·9)	0·8 (−0·2 to 1·9); p=0·084
	FACT-G		89·1 (11·5)	88·7 (12·2)	85·6 (12·5)	82·5 (15·1)	2·8 (1·1 to 4·6); p=0·0003	85·0 (13·1)	82·9 (15·2)	2·6 (0·6 to 4·5); p=0·0045	85·1 (13·8)	82·4 (14·8)	3·7 (1·8 to 5·7); p<0·0001
	Leisure time exercise (Godin)		30·0 (35·7)	31·9 (27·5)	40·6 (29·4)	34·4 (31·0)	8·6 (4·3 to 12·8); p<0·0001	40·2 (38·8)	34·0 (30·0)	6·9 (2·5 to 11·4); p=0·0006	43·7 (49·3)	38·5 (55·6)	7·9 (1·9 to 13·9); p=0·0036
	Fear of cancer recurrence (FCR-4)		9·1 (3·8)	8·6 (3·8)	8·7 (3·7)	8·3 (3·9)	−0·0 (−0·6 to 0·5); p=0·84	8·7 (3·7)	8·1 (4·0)	−0·1 (−0·6 to 0·4); p=0·66	8·8 (3·7)	8·4 (4·1)	−0·2 (−0·8 to 0·3); p=0·36
	Fear of cancer recurrence (FCR-7)		14·9 (7·5)	14·0 (7·2)	14·3 (7·2)	13·8 (7·3)	−0·2 (−1·1 to 0·7); p=0·63	14·4 (7·2)	13·6 (7·5)	−0·4 (−1·4 to 0·7); p=0·43	14·7 (7·1)	14·2 (7·7)	−0·6 (−1·7 to 0·5); p=0·23

Data are n/N (%), or mean (SD), unless otherwise indicated. Missing data imputed via multiple imputation. Higher scores represent better outcomes for all endpoints with the exception of fear of cancer recurrence. FACIT-F=Functional Assessment of Cancer Therapy–fatigue, fatigue subscale scores range 0–52; higher FACIT-F scores indicated lower levels of fatigue. FACT-G=Functional Assessment of Cancer Therapy–general, scores range 0–108, higher scores indicate better quality of life. FACT-P=Functional Assessment of Cancer Therapy–prostate, total scores range 0–156; higher total scores indicate better quality of life. FACT-P physical wellbeing, FACT-P social and family wellbeing, and FACT-P functional wellbeing subscale scores range 0–28; FACT-P emotional wellbeing subscale scores range 0–24; FACT-P prostate cancer subscale scores range 0–48; higher FACT-P subscale scores indicate better quality of life. FCR4=Fear of Cancer Recurrence 4-item questionnaire, scores range 4–20. FCR7=Fear of Cancer Recurrence 7-item questionnaire, scores range 6–40. Godin=Godin Leisure-Time Exercise Questionnaire, scores range more than 0; scores were truncated at the 99th percentile to reduce the influence of extreme outliers on model fit.

* Primary endpoint timepoint.

† Represents the number of questionnaire packs completed and returned.

STAMINA lifestyle intervention was superior for physical wellbeing, social and family wellbeing, emotional wellbeing, and functional wellbeing subscales of FACT-G at 12 months, but not for the prostate cancer subscale (table 2).

Adjusted mean EQ-5D scores were better for STAMINA lifestyle intervention at all follow-up timepoints (3 months 0·81 [SD 0·12] *vs* 0·76 [0·12]; p<0·0001, 6 months 0·79 [0·11] *vs* 0·76 [0·10]; p=0·0022, and 12 months 0·79 [0·10] *vs* 0·76 [0·09]; p=0·0001).

Godin leisure time activity index scores were higher in STAMINA lifestyle intervention at all follow-up timepoints with differences between the arms sustained at 12 months (table 2). No evidence of a difference on fear of cancer recurrence outcomes was demonstrated. Physical outcomes between groups across all timepoints were similar (appendix pp 18–19). Exploratory moderator analyses showed no subgroup differences, and mediation analyses indicated that most effects were direct (appendix pp 20–22).

There were 13 deaths during the trial (six in the STAMINA lifestyle intervention group and seven in the Optimised Usual Care group). Deaths were due to prostate cancer (one in the STAMINA lifestyle intervention group *vs* three in the Optimised Usual Care group), caecal cancer (one *vs* none), sepsis (one *vs* none) and cardiovascular disease (one *vs* two); cause of death was missing in four patients (two *vs* two; appendix p 14). Three intervention-related serious adverse events occurred in the STAMINA lifestyle intervention group (transient loss of consciousness, leg pain or weakness, and back pain); all participants recovered (appendix p 14). No protocol violations were reported (appendix p 15).

The breakdown of health care costs by trial group are shown in table 3. Based on the average observed group size (n=1·92 per supervised session), the mean total cost of sessions was UK£305 (SD 102) whereas the 12-month cost of the STAMINA lifestyle intervention programme was £978 under scenario 1 (ie, £50 per month for the gym membership) and £798 under scenario 2 (ie, £35 per month). In scenario 1, mean incremental costs were £607 and QALYs were 0·036 for STAMINA lifestyle intervention compared with ICER £19 077 per QALY for Optimised Usual Care. In scenario 2, mean incremental costs were £443 and QALYs were 0·036 for STAMINA lifestyle intervention compared with ICER £13 920 per QALY for Optimised Usual Care. Assuming an average session group size of five, the ICERs fall to £11 820 (scenario 1) and £7245 (scenario 2). For the complete-case analysis, incremental costs, QALYs and associated ICERs were: £661, 0·032 and £20 991 (scenario 1) and £500, 0·031 and £15 885 (scenario 2) respectively. Resampling analysis indicated that STAMINA lifestyle intervention had a high chance of being cost-effective (>70% for scenario 1 and >80% for scenario 2) over 12 months (cost-effectiveness acceptability curves in appendix p 23). In a exploratory analysis, over a 10-year horizon, incremental costs, QALYs and associated ICER was £571, 0·06 and £9153 for scenario 1 where STAMINA lifestyle intervention had a 90% chance of being cost-effective (appendix p 24).

**Table 3:** Health-care resource cost, by group

		**STAMINA lifestyle intervention group**	**Optimised usual care group**
Inpatient stays		166·98 (834·35)	116·98 (690·22)
A&E attendance		25·65 (90·41)	32·92 (130·85)
Hospital day centre attendance		105·00 (554·97)	140·95 (612·07)
Outpatient			
	Doctor attendance	349·45 (861·15)	367·66 (888·68)
	Doctor telephone	14·52 (29·18)	15·55 (30·15)
	Nurse or other attendance	229·99 (495·94)	277·77 (582·69)
	Nurse or other telephone	9·86 (16·32)	10·77 (21·60)
Nursing or residential home			
	Nursing or residential home stays	5·40 (121·95)	3·73 (101·45)
	Nursing or residential home visit	0·55 (17·64)	0·11 (2·09)
Hospice			
	Stay	2·38 (48·54)	1·66 (45·06)
	Visit	25·46 (257·23)	50·58 (433·17)
GP			
	Attendance	63·36 (82·48)	69·43 (110·21)
	Home visit	12·80 (81·19)	20·27 (130·86)
	Telephone	16·99 (28·39)	15·60 (27·74)
Practice nurse			
	Attendance	20·63 (26·06)	21·96 (28·67)
	Home visit	3·60 (22·29)	3·80 (22·28)
	Telephone	3·66 (0·69)	3·93 (9·38)
NHS 24 or NHS 111 telephone calls		0·86 (4·20)	1·09 (5·19)
Out of hours GP			
	Attendance	0·65 (7·82)	0·93 (9·48)
	Home visit	..	..
	Telephone	0·06 (1·10)	0·30 (2·99)
District nurse			
	Attendance	1·55 (14·01)	0·60 (7·65)
	Home visit	1·09 (14·85)	1·15 (11·63)
	Telephone	0·13 (1·72)	0·07 (1·41)
Physiotherapist			
	Attendance	18·37 (88·82)	16·86 (100·03)
	Home visit	0·91 (13·21)	0·87 (20·12)
	Telephone	0·17 (1·78)	0·25 (2·13)
Psychologist or counsellor			
	Attendance	7·20 (51·96)	7·46 (49·59)
	Home visit	..	..
	Telephone	0·78 (6·96)	0·71 (5·99)
Home care worker			
	Home visit	0·05 (1·70)	1·07 (21·80)
	Telephone	..	0·02 (0·40)

Data are mean (SD). Costs are in pound sterling. GP=general practitioner. NHS=National Health Service. A&E=accident and emergency.

## Discussion

STAMINA lifestyle intervention provided superior mitigation effects on FACT-P, FACIT-F, FACT-G, and EQ-5D outcomes over Optimised Usual Care, which was probably due to better exercise behaviour, and ICERs supported cost-effectiveness. Cancer-specific quality-of-life benefits exceeded the minimum clinically important difference (MCID) threshold (ie, >3 points on FACT-G)^20^ and provided positive effects across all subdomains. A core part of the trial design that enhances generalisability of the STAMINA lifestyle intervention effects is the involvement of the usual clinical care team liaising with upskilled community-based personal trainers to provide behavioural support and supervised exercise in a national network of hospitals and partnered community fitness centres in rural, smaller inner-city, and larger city locales. To our knowledge, successful integration of lifestyle interventions into core cancer care on this scale has not previously been reported.

Previous studies describe profound deterioration in cancer-specific quality of life and fatigue over 12 months^7^ in men prevented from exercising after ADT initiation. A no-intervention control was precluded during design by (1) previously described deterioration in these patient-reported outcomes, (2) national ethics committee stipulations, (3) extant clinical guidelines, and (4) strong patient and public objections. As such, Optimised Usual Care was developed as a standardised active comparator to provide a consistent and ethically sound basis for comparison in the RCT, although such optimised care is not available in standard practice for patients with prostate cancer. When compared with baseline values, improved exercise behaviour over 12 months was observed in both groups. We deduce that optimisation of usual care involving behaviourally informed clinical staff training, bespoke educational materials, regular behavioural prompting, and safety to exercise checks are a necessary, but insufficient, approach to mitigating adverse effects of ADT. By showing clear benefit of the STAMINA lifestyle intervention over an already enhanced (but not clinically available) comparator, the beneficial treatment effects reported herein probably represent a conservative estimate of the true benefit relative to real-world usual care.

The clinical relevance of our results rests on two main findings. Firstly, men on ADT regularly present to primary and secondary care, in oncology and urology clinics, with symptoms related to their essential cancer treatment but with no evidence-based mitigation provided. STAMINA lifestyle intervention provides a way to substantially offset these distressing problems with an evidence-based intervention delivery pathway that can be integrated into core cancer care across the UK. Recent data from the INTERVAL-GAP-4 trial^10^ show how difficult it is to successfully integrate these core intervention design components into standard care in complex modern health services. Nevertheless, the STAMINA trial has achieved this at scale and within a time frame of 18 months (compared with 7 years of attempted recruitment in INTERVAL-GAP-4). This is particularly noteworthy given that STAMINA recruited participants in the period immediately after the COVID global pandemic, a uniquely hostile environment to the supervised exercise industry and clinical research because of gym lockdowns, social distancing, and revised clinical research priorities. STAMINA offers the most scalable and deliverable model for lifestyle interventions involving supervised structured clinical exercise training published to date and could be viewed as a model template for best practice.

Secondly, from a MCID perspective, we have shown a clinically meaningful change in FACT-G cancer-specific quality of life (>3 points)^20^ and improvements across all four subdomains (physical, functional, emotional, and family and social) that were sustained over 12 months. To date, reliable MCID scores for FACT-P are not available in this patient population; a threshold of 6 points was reported in an end-of-life palliative care population from a post-hoc analysis of a trial (M00–211) that was halted early and unfortunately never formally published.^21^, ^22^ As such, scrutiny of any systematic bias in these data is not possible: MCID thresholds derived from anchor-based statistics in this context are problematic.^23^ With regards to fatigue, the FACIT group's guidance indicates that important differences typically cluster around 5% of possible scale scores; on the 0–52 FACIT-F scale, this translates to a threshold of 2–3 points. Our reported effect of 1·9 points is similar to such differences, and this score are represented in the between-group 97·232% CIs at 12 months (0·4–3·4).

Cost-effectiveness data from lifestyle interventions in cancer populations are scant in the evidence base, and the data from the STAMINA trial directly facilitate implementation. A recent comprehensive analysis found that the median ICER for NICE-approved oncology drugs was £30 000 (£22 395–£45 870).^24^ STAMINA's ICER of £13 920–£19 077 per QALY falls substantially below these medians. Thus, the STAMINA lifestyle intervention represents better value for money than most cancer drugs currently approved and funded by the NHS.

While leading to a modest additional cost, driven mostly by gym membership costs, the economic evaluation indicated that at 12 months, the STAMINA lifestyle intervention generates sufficient QALYs to be cost-effective. Using the most conservative inputs (ie, mean of 1·92 participants per exercise session and higher cost gym membership fees), it remains highly likely (>70%) to be cost-effective. Furthermore, post-hoc 10-year modelling suggests that the STAMINA lifestyle intervention is even more likely to be cost-effective over the longer term.

We can hypothesise that the STAMINA intervention generates significantly better effects than Optimised Usual Care due to higher volumes of physical activity. However, STAMINA is a clinical effectiveness RCT, not a dedicated mechanistic study. The exercise prescription in STAMINA was calibrated for integrated delivery, long-term sustainability and adherence in a real-world clinical setting, rather than for maximal physiological adaptation, which is a different research question requiring different primary outcomes. No difference was recorded in physical outcomes; however, both groups received active interventions, which might have attenuated between-group differences. The exploratory complier average causal effect analysis did not rule out a dose–response relationship between intervention receipt and benefit. The wide CIs in this analysis reflect the expected reduction in statistical power inherent in non-powered secondary analyses. Quality-of-life benefits might operate through psychosocial mechanisms alongside fatigue, which has been discussed in detail in other reviews of the topic.^25^

Strengths of the STAMINA trial include the embedding into core cancer services, the bespoke referral and triage system developed, the national network of delivery centres created, the number of staff trained, and the number and rate of participant recruitment and randomisation. The trial also had good long-term follow-up retention (85% completing 12-month primary outcomes), good adherence to the STAMINA lifestyle intervention, included a variety of study recruiting centres (major treatment centres and district general hospitals in both rural and urban communities), a strong selection of clinically relevant outcomes, a comorbidity profile of participants reflective of the general population presenting with prostate cancer, and long follow-up duration. Long-term change in exercise behaviour is essential for meaningful benefits, and adherence over 12 months was well maintained in the STAMINA trial.

Limitations include the small absolute numbers of minority ethnic participants, as well as the relatively high proportion of men self-rating themselves as fit. The ethnic composition of the study population is similar to the general population aged 70 years or older in the UK. Whether the intervention would have similar efficacy in minority groups remains open to question, but this can be addressed firstly through acceptability testing during implementation and, if necessary, phase 3 trials, if there is plausible scientific reason to indicate the possibility of differential effects. The treatment landscape in prostate cancer continued to evolve during the trial period: although only one in five men recruited were receiving androgen receptor pathway inhibitors, the nearly 30% of recruits with documented metastatic disease represents the largest such population taken directly from clinical practice in such a trial to date. There is no biological rationale to expect that the intervention would be less effective in men receiving escalation therapy; indeed, such men experience greater treatment-related toxicity and could potentially derive superior benefit from the intervention. With regard to the self-rating of physical fitness, although there is reasonable correlation between self-rating and objective testing of fitness, usual experience in surgical practice suggests men often overestimate their level of fitness; this is supported by the high level of comorbidity present and was often evident during sub-maximal testing. The differences in our primary outcomes were not as large as those detected in our previous, much smaller, single-centre study (included in the present sample size estimate) and dilution of treatment effects is common in pragmatic versus efficacy studies. Furthermore, the current trial employed an active intervention as comparator (rather than a traditional control used in the phase 3 RCTs) due to ethical considerations. Despite this, the STAMINA lifestyle intervention showed clinically important superiority in cancer-specific quality of life. It is also important to reiterate that Optimised Usual Care is not generally available in clinical practice. In addition, although dietary advice was given as part of STAMINA lifestyle intervention, we did not measure to what extent this was followed (eg, analysis of food diaries) or what impact it had on trial outcomes. The use of multiple imputation for missing data requires some mention. Our primary analysis used a pattern mixture modelling framework, which explicitly allows the distribution of outcomes for patients with missing data to differ from those observed, rather than assuming they are the same, thus enabling analysis under plausible missing not at random assumptions. However, we acknowledge that this might not fully address the missing not at random assumption, and mixed-effects modelling might further illuminate this possibility. However, the consistency of results across sensitivity analyses suggests these factors are unlikely to have affected our conclusions.

Null effects of a 12 week community exercise intervention^26^ and 12 months of supervised exercise and diet advice^27^ have been reported by trials using other cancer-specific quality-of-life outcomes (the European Organisation for Research and Treatment of Cancer [EORTC] quality-of-life core 30 items, EORTC quality-of-life patient-reported 25 items, and Expanded Prostate Cancer Index Composite [EPIC] 26 items questionnaires), but these studies ultimately failed to recruit to target and, as such, were underpowered to detect differences.^26^, ^27^ Similarly, Taaffe and colleagues^28^ reported no difference at 12 months in cancer-specific fatigue across three active intervention groups, all of which included at least 6 months of supervised exercise at the 12 months assessment. The inclusion of supervised exercise in all groups might have diluted an observable effect. Null results have also been reported after 6 months in a large trial (n=463) of telephone-based peer support and self-management materials to promote physical activity in men on ADT.^29^ No benefit was seen in cancer-specific quality-of-life data at 12 months evaluated using symptom subscale of the Expanded UCLA Prostate Cancer Index Composite questionnaire (EPIC) questionnaire. This might reflect inadequate strategies for behaviour modification, for instance, ongoing supervision.

Data from the 2025 phase 3, multicentre CHALLENGE trial showed that supported exercise can improve overall survival.^30^ Two important distinctions exist between the STAMINA trial and the CHALLENGE study. The first is the duration of recruitment; all 700 men in STAMINA were recruited within an 18-month window, whereas the CHALLENGE study recruited at a rate equivalent to approximately one individual per centre, per year, over a total of 15 years, limiting generalisability of the data. Secondly, CHALLENGE examined survival, where STAMINA focuses on quality of life. The importance of quality of life in cancer survivors cannot be overstated. A 2025 meta-analysis of RCTs reported that improvements in quality of life and lower fatigue translate to better overall survival.^31^

In conclusion, the STAMINA lifestyle intervention offers clinically important benefits and is cost effective compared with Optimised Usual Care, which itself is likely to be superior to real-world usual clinical practice. Given the absence of strong, evidence-based interventions to mitigate the adverse effects of ADT, this study offers clinicians a clear basis for identification, referral, and prescription of supervised exercise and diet advice interventions to minimise adverse effects associated with treatment, and critically, meet best practice guidelines for patients. Although the STAMINA intervention can be implemented within NHS England, to our knowledge, the STAMINA design serves as the most widely generalisable basis for delivery of a supervised exercise and dietary advice intervention to mitigate negative effects associated with ADT.

### Contributors

### Data sharing

Sheffield Teaching Hospitals NHS Foundation Trust will have the final decision on whether the access can be granted. Access to data and related documents shall be subject to the exchange of a data sharing agreement (DSA) with the sender of the data. The Coordinating Centre and Clinical Trials Unit (CTRU) reserve the right to recover applicable fees to be agreed with the requestor for preparation of the data for this purpose. A STAMINA Legacy Data Access Committee consisting of the Sponsor (Sheffield Teaching Hospitals NHS Foundation Trust), Coordinating Centre (Sheffield Hallam University) and the CTRU (University of Leeds) has been formed to govern the use of the STAMINA data now that the STAMINA programme has closed. All data shared with third parties will be fully anonymised and the terms of the DSA will forbid the receiver to attempt to re-identify participants. Contact lead author at derek.rosario@nhs.net or CTRU-DataAccess@leeds.ac.uk in the first instance.

## Declaration of interests

All grant co-applicants had a proportion of their salary covered as direct costs of carrying out the research. Co-applications were also eligible to claim travel expenses to attend all scheduled programme meetings. LB, SR, DJR and SI have received a grant (Nov, 2024) to train Nuffield Health staff involved in their charitable social return on investment programme. SI and SR received a follow-up NIHR grant from the STAMINA PGfAR (PDG-B). SR received payment from Nuffield Health and Different Drum for private Behavioural Science consultancy work (projects related to community-based physical activity programmes, workforce restructuring and cancer screening). AI and DD are employees of Nuffield Health. JB received charity grants from UK Breast Cancer Now, UK Yorkshire Cancer Research, and Sheffield Hospitals charity, consulting fees from Ipsen, Eisai, Fresenius Kabi, Merck, payment or honoraria from Ipsen, Eisai, support for meetings from Ipsen, and is the president of the Cancer and Bone Society. AF is a member of the NIHR international funding committee, clinical trials unit standing advisory committee and the NIHR COVID prophylaxis platform study in care homes funding committee, and is an NIHR senior investigator. MC participates in NIHR advisory boards for: STRIVE, iPREHAB, PERFORM and is a member of the NIHR HTA funding committee and the Cross NIHR collaboration for multiple long-term conditions. All other authors declare no competing interests.
