# Supplementary material for: An integrated lifestyle intervention for mitigation of adverse effects associated with androgen deprivation therapy for prostate cancer (STAMINA): a multicentre, randomised trial
Source: Lancet Oncol. 2026 Oct;27(10):1283–94. doi: 10.1016/S1470-2045(26)00304-9 (PMC13619545; doi:10.1016/S1470-2045(26)00304-9)
Supplement: Supplementary file 1 [file mmc1.pdf]

THE LANCET
Oncology

Supplementary appendix

This appendix formed part of the original submission and has been peer reviewed. We post it as supplied by the authors.

Supplement to: Bourke L, Rosario DJ, Reale S, et al. An integrated lifestyle intervention for mitigation of adverse effects associated with androgen deprivation therapy for prostate cancer (STAMINA): a multicentre, randomised trial. *Lancet Oncol* 2026; published online Sept 4. https://doi.org/10.1016/S1470-2045(26)00304-9.

Appendix 1: Substantial Amendments

Amendment SA01 Submitted 06/08/2021

**Protocol version: 5.0 05/07/2021 (Implemented prior to start of trial recruitment)**	
**REC approval**	**23/08/2021**
**HRA approval**	**01/09/2021**
**Details**	Due to the COVID19 pandemic the STAMINA programme has been amended. The intervention has been changed to enable a blended approach between face to face and remote delivery. This approach has been piloted in a separate, additional pilot study (STAMINA WP3 (IRAS Ref: 253778)). The protocol has been amended to reflect the changes in trial design, from a cluster randomised controlled trial to an individually randomised controlled trial, which in turn has meant a change in sample size. Two further secondary outcome measures have been added. In addition, the trial team has made a number of revisions to the protocol and information sheets in the interests of clarity.

Amendment SA04 Submitted 27/06/2022

**Protocol version: 7.0 18/05/2022**	
**REC approval**	**20/07/2022**
**HRA approval**	**22/07/2022**
**Details**	This amendment consists of 5 areas of change:<br>• Patients will be asked during informed consent if they would be willing to wear heart rate monitors during their exercise sessions, should they be allocated to the STAMINA Lifestyle Intervention. Heart rate monitors will be given to all participants at a sample of Nuffield Health gyms by the Clinical Exercise Specialists.<br>• The number of participating sites has been reduced from approximately 22 to a minimum of 12. On the basis of our experience so far, and communication with participating organisations, it is envisaged that fewer participating sites will be able to recruit sufficient participants to reach the required sample size. This will save time and resources spent on site set-up for both the research team and the NHS.<br>• We have amended the Study Protocol to reflect the above 2 substantial changes. We have also made some minor clarifications as outlined below (change 1).<br>• We have made minor changes to a number of study documents, and created 3 new documents as well as 2 videos to support participants with the heart rate monitor usage, as outlined below (change 3).<br>• We are adding the possibility to use Participant Identification Centres, see list of PICs (change 5) and we are submitting a template mNC_PIC agreement.

Amendment SA06 Submitted 16/12/2022

**Protocol version: 8.0 07/12/2022**	
**REC approval**	**16/01/2023**

**HRA approval**	**20/01/2023**
**Details**	There is a new unvalidated participant questionnaire (Discrete Choice Experiment) to be given to participants to complete at the 12-month time point. We have also created a poster to be put up in waiting rooms to alert patients to the trial, and amended/clarified the progression criteria for the internal pilot in the protocol.

Amendment SA09 Submitted 18/07/2023

**Protocol version: 10.0 05/07/2023**	
**REC approval**	**04/08/2023**
**HRA approval**	**18/08/2023**
**Details**	We have clarified the follow-up process, amended current follow-up documents to encourage completion and created new documents including a postcard and poster from the PPI group. 3 new questions will be included in the ADT Symptom Index questionnaire at 12 months only for participants to complete. We have also amended the topic guide for the Process Evaluation interviews, made minor administrative protocol changes, made minor amendments to the Discrete Choice Experiment (DCE) and included a new letter, included the interviewer version of the EQ-5D-5L, and included a site who has changed Trust name.

Amendment SA10 Submitted 08/02/2024

**Protocol version: 11.0 01/02/2024**	
**REC approval**	**23/02/2024**
**HRA approval**	**28/02/2024**
**Details**	Participants will receive an unconditional £10 gift voucher to support all methods of follow-up at 12 months post-randomisation. Participants who opt for postal questionnaires will receive this alongside their postal questionnaire; participants who opt for online questionnaires will receive this in the post. All participants continuing to take part in the study who have already passed their 12 month questionnaire due date will be sent the voucher retrospectively in the post. Participants within 3 months of their 12 month due date will be resent the questionnaire to complete alongside the voucher. Letters have been created/amended to support these processes.

Non-Substantial Amendments:

Amendment NSA02 Submitted 14/12/2021

**Protocol version: 6.0 18/11/2021**	
**REC approval**	N/A - acknowledgement from NHS Research Ethics is not required for non-substantial amendments
**HRA approval**	14/12/2021

**Details**	We have amended the Participant Information Sheet and Consent form to include using participant data for future research. We have also made administrative changes throughout the study documents to ensure it accurately reflects the data collection and processes

Amendment NSA03 Submitted 29/04/2022

**Protocol version: 6.0 18/11/2021**	
REC approval	N/A - no regulatory review required
HRA approval	N/A - no regulatory review required
**Details**	Addition of participating NHS sites that were not previously listed in part C of the IRAS application.

Amendment NSA05 Submitted 01/12/2022

**Protocol version: 7.0 18/05/2022**	
REC approval	N/A - no regulatory review required
HRA approval	N/A - no regulatory review required
**Details**	Addition of participating NHS sites that were not previously listed in part C of the IRAS application.

Amendment NSA07 Submitted 19/01/2023

**Protocol version: 9.0 16/01/2023**	
REC approval	N/A - no regulatory review required
HRA approval	N/A - no regulatory review required
**Details**	Participants will be randomised to receive difference choices within the Discrete Choice Experiment questionnaire; this process has been clarified in the protocol. We have updated the topic guide for the Process Evaluation to include questions regarding the use of heart rate monitors.

Amendment NSA08 Submitted 09/05/2023

**Protocol version: 9.0 16/01/2023**	
REC approval	N/A - no regulatory review required
HRA approval	N/A - no regulatory review required
**Details**	We are extending the recruitment period from its original end date to 31 August 2023, and the follow-up period to 31 August 2024. The overall trial end date has been

	extended to 31 March 2025. The overall recruitment target of 697 participants remains unchanged. This extension is done with the knowledge and support of the funder. Sites will continue to be paid as per the mNCA schedule.

Amendment NSA11 Submitted 17/06/2024

**Protocol version: 11.0 01/02/2024**	
REC approval	N/A - no regulatory review required
HRA approval	N/A - no regulatory review required
**Details**	Change in PI at one site

APPENDIX 2: STAMINA lifestyle intervention and optimised usual care further details

Optimised usual care (all patients): clinical staff treating men with prostate cancer in participating Urology and Oncology departments received bespoke training to recognise men on ADT who were eligible for STAMINA, recommend exercise training as part NICE guidelines, refer men to the research team and record their participation in the medical notes (The 4 'Rs'). Training included education on National Institute for Health and Care Excellence (NICE) and European Association of Urology (EAU) best prostate cancer ADT practice clinical guidance (e.g. weight control, calorie management, exercise guidelines and behavioural change techniques) to support men in making and maintaining these changes during clinical follow-up. Participants were sent publicly available information booklets for signposting: 1. 'Prostate Cancer: a guide for men who've just been diagnosed' (Prostate Cancer UK, 2019), 2. 'Living with hormone therapy: a guide for men with prostate cancer' (Prostate Cancer UK, 2022) 3. 'Healthy eating with cancer' (Macmillan, 2020) and 4. 'Physical activity and cancer' (Macmillan, 2019). All patients received behavioural prompts and monitoring from their clinical care team at routine clinic visits. Following referral, a 'safe to exercise' check, based on extant guidelines from the Association of Chartered Physiotherapists in Cardiovascular Rehabilitation[1] (https://www.acpicr.com/publications/healthcare-professionals) and the American College of Sports Medicine[2], was carried out by a trial researcher remotely, prior to randomisation.

STAMINA lifestyle intervention (SLI): in addition to receiving optimised usual care, those men randomised to STAMINA lifestyle intervention received a supervised exercise programme including both aerobic and resistance components with dietary advice in Nuffield Health (NH) fitness and wellbeing centres. SLI is a behaviourally informed, lifestyle intervention embedded into prostate cancer care to support delivery of NICE (NG131 1.4.19) guidance for people with prostate cancer on ADT. SLI combines resistance and aerobic exercise with application of the behavioural skills learnt to other patient-negotiated lifestyle goals, including dietary modification. All clinical exercise specialists (CES) delivering SLI were experienced community personal trainers upskilled through focused tuition, including training in an exercise prescription previously trialled in men on ADT,[3] education on prostate cancer treatments and adverse effects, provision of behavioural support and basic research methodology (e.g. principles of GCP). SLI participants were invited to an induction with a (CES) to explore their capability, opportunity, and motivation to exercise twice a week for 12 months. Following a 1:1 sub-maximal exercise test, supervised exercise sessions were tailored to individual capability initially delivered 1:1 before swift transition to small group delivery (maximum group size ≤5). The supervised exercise sessions included both moderate intensity aerobic (30–45 min) and resistance (up to 4 sets, 8–12 reps of major muscle groups) components as recommended by NICE (NG131 1.4.19) and in accordance with exercise programmes previously shown to be beneficial and safe to men on ADT.[3]

Supervision was carried out twice weekly for 12 weeks, with supervision frequency negotiated between the participant and CES (maximum once a month, minimum once every 3 months) over the subsequent 9 months (12 months in total). During the 12-month intervention period, SLI participants were provided with a complimentary NH gym membership to support additional, independent exercise behaviour. Additional behaviour change support was provided during scheduled programme reviews and catch-up calls and by completion of a behaviourally informed diary. Exercise staff sent progress reports to the clinical team at 3, 6 and 12 months. SLI was developed in line with two overarching principles: (1) SLI will be offered as an

intrinsic part of a man's cancer care as opposed to an 'add-on' and (2) the intervention will be designed in a way to maximise both efficacy (likelihood of change) and effectiveness (accessibility and generalisability beyond urban areas). Programme reviews were scheduled at 12 weeks, 6 months and 12 months following the induction session and included a review of participant progress and a written summary report. Summary reports were sent to the referring clinical team via email as part of the feedback loop and for discussion with the participant at their next routine clinic appointment.

Catch-ups (face-to-face or over the phone) were organised with the CES and participant every 6 weeks to explore participant progress and address any barriers to intervention adherence (where applicable). SLI participants were be offered one reduced price membership (~ 50%) for a family member to further support their exercise behaviour by addressing social support.

Appendix 2 references

1 Association of Chartered Physiotherapists In Cardiovascular Rehabilitation. Healthcare professional guidance https://www.acpicr.com/publications/healthcare-professionals/. Accessed 10/10/22.

2. Riebe, D., Ehrman, J.K., Liguori, G. and Magal, M. Wolters Kluwer, Philadelphia, PA, 2018 ACSM's guidelines for exercise testing and prescription: American College of Sports Medicine; 10th Edition; 2018.

3. Bourke L, Gilbert S, Hooper R et al. Lifestyle changes for improving disease-specific quality of life in sedentary men on long-term androgen-deprivation therapy for advanced prostate cancer: a randomised controlled trial. Eur Urol. 2014; 65:865–872.

Appendix 3: Statistical methods further details

The STAMINA Statistical Analysis Plan (SAP) provides full details of the statistical methods used within the trial.

Potential clustering at the level of the CES in the SLI arm was accounted for using an intracluster correlation coefficient (ICC) of 0.05 [1] and a coefficient of variation of 0.6 accounts for the variation in the number of men seen by each CES. This resulted in a total sample size of 697 men; 384 in the SLI arm and 313 in the OUC arm (5:4 ratio). An average cluster size of 6 participants per CES (range 1-14) required 64 CESs. We compared outcomes between arms using a generalized linear mixed-effects regression model adjusting for minimisation strata (with age treated as continuous), baseline score of the dependent variable and site. Results were expressed as adjusted mean differences (MD, SLI-OUC) with 97.232% confidence intervals (CI), p-values and ICCs (where appropriate). We planned a priori to compare outcomes using a partially nested model accounting for clustering in SLI due to CES delivery [2] however this was not possible as the average cluster size was too small. SLI was deemed clinically different to OUC if a statistically significant result was observed in either primary outcome at 12-months post-randomisation. Statistical testing used two-sided 2.768% significance levels.

We adopted a while-on-treatment estimand strategy, treating death as precluding observation of the outcome. Accordingly, we did not impute data after death, and the treatment effect was evaluated only among participants who survived to the primary outcome timepoint.[3] Sensitivity analyses assessing robustness of primary outcome results included complete case analysis and clustering of SLI participants according to NH gym attended. Assumption checking for all models used raw residual and influence plots. Intervention delivery, adverse effects of ADT, physical outcomes, deaths and RUSAEs were summarised descriptively.

Missing Data

To identify participant baseline characteristics associated with missingness and to assess for differences between arms we explored missing participant outcome data overall, by treatment arm, by baseline characteristics and by baseline characteristics between arms and by site. Data were examined to assess whether data was missing at random (MAR), missing completely at random (MCAR) or missing not at random (MNAR). The missing data pattern of the following variables were investigated:

- Missing outcome data (including corresponding baseline scores)
- Missing participant-level covariate data measured at baseline
    - Age
    - Duration on ADT
    - Receiving chemotherapy and/or novel ARI
    - Receiving radiotherapy
    - Preferred method of outcome data completion
- Missing CES-level covariate data
    - Missing CES ID

For all outcomes, if the data was not considered to be MCAR and >5% of observations were

missing/invalid and would therefore not contribute to the final analysis model, imputation was used. Missing outcome data due to participant death was not imputed in the analysis models.

Multiple imputation used pattern-mixture modelling (PMM) to impute missing values which allows the imputation model to incorporate and assess departures from the MAR assumption. PMM is a recognised approach for handling missing data in the presence of differential patterns of missingness for example if there is higher dropout in the control arm compared to the intervention arm, indicating differential missingness by treatment allocation and raising the possibility that the missingness mechanism may differ between arms. While we acknowledge that MNAR mechanisms cannot be definitively verified using MI-based approaches alone, the use of PMM enables an appropriate assessment of the potential impact of such departures on the estimated treatment effects.

All pre-specified primary analysis covariates were included in the imputation models, alongside the preferred method of questionnaire completion (electronic or postal) and the corresponding outcome variables from earlier timepoints. We additionally conducted a stepwise regression analysis to determine if either the participants baseline level of fitness or whether the recruiting site was a specialist cancer centre should be included in the imputation model for the primary outcomes. Including auxiliary variables that are associated with the outcome or the missingness mechanism is generally recommended when specifying imputation models. However, in this case, the excluded variables were not associated with either the outcome nor the probability of missingness, and therefore were unlikely to contribute meaningfully to the imputation. Their omission is thus unlikely to have affected the validity or efficiency of the estimates. The final imputation models therefore included only the pre-specified primary analysis covariates and corresponding outcome variables from previous timepoints, with the addition of method of questionnaire completion. 20 imputation analyses were performed with the estimates combined using Rubin's rules. The same strategy was followed for the secondary outcomes.

CACE Analysis

Complier Average Causal Effect (CACE) analysis[4] was used to estimate the effect of an intervention amongst compliant participants. The CACE is defined as the average effect of treatment in the compliers. Compliers are defined as participants who would have received an effective dose of the intervention had it been offered. Analysis takes non-adherence to the intervention into account, comparing treatment received rather than treatment allocated. The CACE approach assumes four types of compliance status: compliers, never takers, defiers and always takers:

1.Always-takers will always receive the intervention irrespective of their allocation
2.Never-takers will never receive the intervention irrespective of their allocation
3.Compliers receive the intervention if and only if they are allocated to the SLI arm
4.Defiers receive the intervention if and only if they are allocated to the OUC arm

The CACE population consisted of all randomised participants. Compliance with the SLI intervention was defined as the proportion of prescribed exercise sessions completed out of all prescribed exercise sessions. Each participant allocated to SLI was expected to complete 2 sessions per week during the first 12 weeks of the intervention (24 sessions in total). Over the remainder of the 12-month intervention period, the prescribed number of sessions was agreed between each participant and their CES at progress reviews which took place at regular

intervals. The number of prescribed exercises sessions varied by participant. Participants who completed 21 or more prescribed exercise sessions, including at least 18 during the first 12 weeks of intervention delivery, were classed as compliers.

The PROC QLIM procedure in SAS version 9.4 was used to conduct the CACE analysis. Compliance and each outcome are modelled jointly, with each primary outcome model including compliance as the exposure of interest. Randomised allocation acted as an instrumental variable for compliance, allowing estimation of the treatment effect among participants who complied with the intervention.

Mediation Analysis

Causal mediation analysis was conducted for the primary endpoints considering the following as potential mediators:

- Godin activity level
- A Body Shape Index (ABSI)
- FACIT-F at 6 months (as a mediator for FACT-P only)

As STAMINA participants are randomised to their treatment arms, the mediation analysis did not need to account for confounding between treatment and either the mediator or outcome. Mediation models were adjusted for the same covariates as the primary analysis including baseline outcome. Additionally, baseline levels of the mediators were included in the mediator equation to account for pre-existing differences. By adjusting for observed confounders, we assumed that any residual unmeasured confounding is negligible. An interaction between the mediator and treatment was included in the mediation model to allow the mediator effect to vary across treatment arms.

As the potential mediators are continuous, the following models were fitted in Stata version 18.0, where m1 denotes the continuous mediator variable, m1baseline represents the corresponding baseline mediator level, and x1,…,xn denotes the additional covariates:

Mediate (FACT-P total score x1,…,xn) (m1 m1baseline) (treatment)
Mediate (FACIT-F score x1,…,xn) (m1 m1baseline) (treatment).

The total effect of treatment on each primary outcome was estimated, along with the direct and indirect effects (i.e. the component of the total effect mediated through m1). The proportion of the total effect attributable to the mediator was also calculated.

Appendix 3 References

1.Iliffe S, Kendrick D, Morris R, et al. Multicentre cluster randomised trial comparing a community group exercise programme and home-based exercise with usual care for people aged 65 years and over in primary care. *Health Technol Assess*. 2014;18(49):1–105.

2. Flight L, Allison A, Dimairo M et al. Recommendations for the analysis of individually randomised controlled trials with clustering in one arm - a case of continuous outcomes. *BMC Med Res Methodol*. 2016;16(1):165.

3.Ratitch B, Bell J, Mallinckrodt C et al. Choosing Estimands in Clinical Trials: Putting the ICH E9(R1) Into Practice. *Ther Innov Regul Sci* 2020; 54:324-341.

4. Connell AM. Employing complier average causal effect analytic methods to examine effects of randomized encouragement trials. Am J Drug Alcohol Abuse. 2009;35(4):253-9.

Appendix 4: Economic evaluation

Resource use was valued using national unit costs, including the NHS Reference Costs [1] and Personal Social Services Research Unit guidance (PSSRU). [2]

Table 1: Resource use - Unit costs and assumptions

Item	Type	Cost (£ per unit)	Source
Inpatient	Short stay	1,395.19	NHS Ref Costs 21/22: NES, non-elect short stay, malignant- Acute. Inflated from Jan 2022 to June 2024
	Long stay	376.78	NHS Ref Costs 21/22: RP, non-elect long stay, malignant - Acute. Inflated from Jan 2022 to June 2024
A&E	Attendance	210.39	NHS Ref Costs 21/22: Emergency Medicine, VB09Z (Type 01-04). Inflated from Jan 2022 to June 2024
Hospital day centre	Attendance	1,111.00	PSSRU 2023: Pg 36 - Elective/non-elective day cases (HRG)
Outpatient:			
Doctor	Attendance	243.00	PSSRU 2023: Pg 36 - Medical specialist palliative care (19+)
	Telephone	20.27	PSSRU 2023: Pg 67 Table 9.6.1 GP-led triage
Nurse	Attendance	92.00	PSSRU 2023: Pg 36 - Non-medical palliative care (19+)
	Telephone	9.04	PSSRU 2023: Pg 67 Table 9.6.1 Nurse-led triage
Nursing/ residential home	Stay (per night)	197.00	PSSRU 2023: Pg 7 Table 1.1.1 Establishment + living costs
	Home visit	40.25	Leeds City Council: Adult Social Care Costs. https://www.leeds.gov.uk/adult-social-care/how-much-it-costs
Hospice	Stay	305.00	PSSRU 2023:: Pg 36 Table 6.1.1 Inpat. specialist palliative care
	Visit	243.00	PSSRU 2023: Pg 36 Table 6.1.1 Outpat. specialist palliative care
GP	Attendance	49.00	PSSRU 2023: Pg 64 Table 9.4.2 - 10-minute consultation
	Home Visit	245.00	PSSRU 2023: Pg 64 Table 9.4.2 Based on 50-min. (NICE NG94)
	Telephone	20.27	PSSRU 2023: Pg 67 Table 9.6.1 GP-led triage
Practice nurse	Attendance	13.69	PSSRU 2023: Pg 62 Table 9.3 - 15.5 min. appointment
	Home visit	57.42	PSSRU 2023: Pg 62 Table 9.3 Based on 65.5 min.(NICE NG94)
	Telephone	9.04	PSSRU 2023: Pg 67 Table 9.6.1 Nurse-led triage
NHS 24 or NHS 111	Telephone	13.33	Turner et al. (2021). Health Serv Deliv Res 2021;9(21). Inflated from £11.40 in 2021 to June 2024
Out of hours GP	Attendance	94.38	National Audit Office (2014). OOH GP services in England. Inflated to June 2024
	Home Visit	245.00	PSSRU 2023: Pg 64 Table 9.4.2 Based on 50-min (NICE NG94)
	Telephone	20.27	PSSRU 2023: Pg 67 Table 9.6.1 GP-led triage
District nurse	Attendance	62.85	Ball et al. (2014) NHS Ref Costs 21/22: £53.74: N02AF. Inflated to June 2024
	Home Visit	84.93	Ball et al. (2014) NHS Ref Costs 21/22: £53.74: N02AF. Inflated to June 2024
	Telephone	9.04	PSSRU 2023: Pg 67 Table 9.6.1 -14.4% of in-person contact
Physiotherapist	Attendance	78.00	PSSRU 2023: Pg 36 Table 6.1.1 One-to-one session
	Home Visit	88.14	Ball et al. (2014), King's College London:+ 13% for travel time
	Telephone	11.23	PSSRU 2023: Pg 67 Table 9.6.1 - 14.4% of in-person contact
Psychologist/ Counsellor	Attendance	63.00	PSSRU 2023: Pg 56 - Band 7, 1 hour
	Home Visit	71.19	PSSRU 2023:: Pg 56 - Band 7 + 13% for travel (Ball et al. 2014)
	Telephone	9.07	PSSRU 2023:: Pg 56 - 14.4% of attendance cost
Home care worker	Home Visit	27.00	PSSRU 2023: Pg 78 - One-hour visit
	Telephone	3.89	PSSRU 2023: Pg 78 - 14.4% of visit cost

Table 2: Intervention development and delivery costs (£)

Category		Facilitator (SH)	NHS	Nuffield	Total
**1. Set-up Costs**					
Primer meetings		940.52	569.31	286.67	1,796.50
Training		7,016.65	8,149.99	3,802.44	18,969.08
Travel costs		3,893.80	-	1,678.23	5,572.03
Accommodation		2,761.95	-	-	2,761.95
Refreshments & room hire		79.30	-	-	79.30
Materials		6,020.11	-	-	6,020.11
Website		1,795.00	-	-	1,795.00
**Total setup costs**		**22,507.33**	**8,719.30**	**5,767.34**	**36,993.97**
Denominator		[a]	[a]	[a]	[a]
**Per-participant setup cost:**		**4.92**	**1.91**	**1.26**	**8.09**
**2. Ongoing support time costs**					
a. NHS-based support		1,202.01	1,000.51	-	2,202.52
Denominator		[b]	[b]		[b]
**Per-participant cost:**		**0.79**	**0.66**	**-**	**1.44**
b. Nuffield-based support		2,394.30	-	1,395.10	3,789.40
Denominator		[c]		[c]	[c]
**Per-participant cost:**		**6.30**	**-**	**3.67**	**9.97**
**Totals:**	**SLI (1 +2.a +2.b)**	**12.01**	**2.56**	**4.93**	**19.50**
	**OUC (2.a)**	**0.79**	**0.66**	**-**	**1.44**

Denominators: [a] Referred x 3 years = 4575; [b] Referred = 1575 =; [c] nSLI = 380

Table 3: Unit costs of STAMINA Lifestyle Intervention for SLI participants

Item		Unit Cost	Additional information
Supervision		£16.51	Clinical Exercise Specialist: Hourly rate as per Nuffield Health
Gym Induction		£8.26	30 minutes of CES time
Programme Reviews		£16.51	1 hour of CES time for longer reviews at 12, 26 and 52 weeks.
Safety to Exercise Check		£28.50	30 minutes Clinical Exercise Physiologist time. PSSRU 2024 pg. 64: £57 per hour.
Gym Membership:	Scenario 1	£50.00	Off-peak rate for Nuffield Health (not including London sites)
	Scenario 2	£35.00	Typical monthly cost for mid- to lower-range gyms
Implementation Costs		£19.50	See Table 2

Appendix 4 References

1. England NHS, Improvement NHS. National Cost Collection 2023-24 Report [Internet]. https://www.england.nhs.uk/costing-in-the-nhs/national-cost-collection/ (Accessed 01/08/25)
2. Curtis, L. and Burns, A. Unit Costs of Health and Social Care 2016. Canterbury, UK: Personal Social Services Research Unit, The University of Kent, 2016.

Appendix 5: Comorbidities by Arm

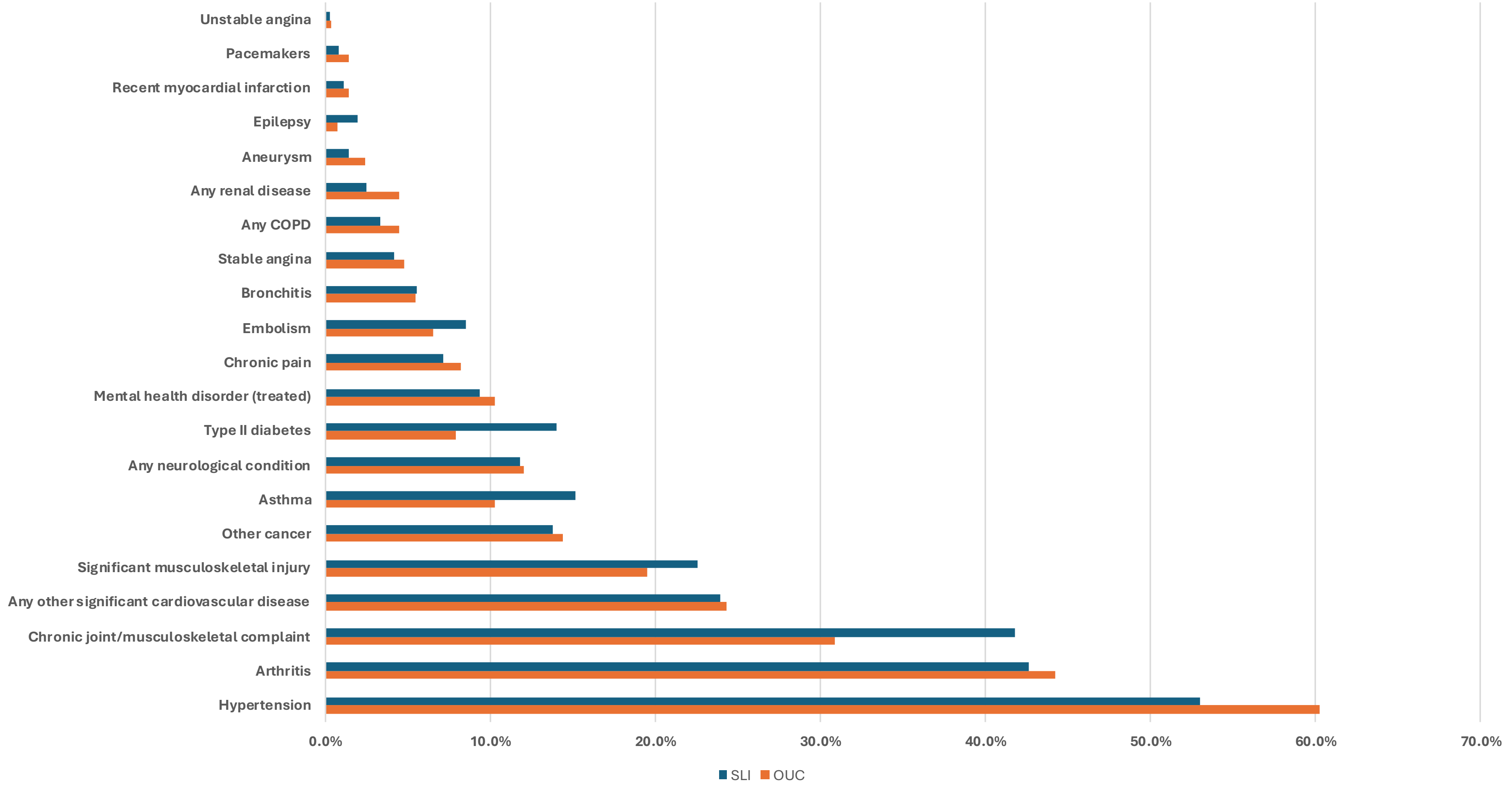

Appendix 6: Presence of Cardiorespiratory and Musculoskeletal comorbidities

	STAMINA Lifestyle Intervention (n=389)	Optimised Usual Care (n=311)	Total (n=700)
**Cardiorespiratory comorbidities**[1]			
No cardiorespiratory comorbidities	118 (30.3%)	88 (28.3%)	206 (29.4%)
Hypertension and/or DM with no other cardiorespiratory comorbidities	108 (27.8%)	94 (30.2%)	202 (28.9%)
At least one cardiorespiratory comorbidity (± Hypertension and/or Diabetes)	163 (41.9%)	129 (41.5%)	292 (41.7%)
**Musculoskeletal comorbidities**[2]			
No musculoskeletal comorbidities	112 (28.8%)	108 (34.7%)	220 (31.4%)
Musculoskeletal comorbidities	277 (71.2%)	203 (65.3%)	480 (68.6%)
Total	389 (100%)	311 (100%)	700 (100%)

Appendix 7: Information About Participant Death

	STAMINA Lifestyle Intervention (n=389)	Optimised Usual Care (n=311)	Total (n=700)
**Death reported (%)**[3]	6 (1.5%)	7 (2.3%)	13 (1.9%)
Of those who died:			
**Primary cause of death**			
Prostate cancer related	1 (16.7%)	3 (42.9%)	4 (30.8%)
Recurrent caecal cancer	1 (16.7%)	0 (0.0%)	1 (7.7%)
Sepsis of unknown source	1 (16.7%)	0 (0.0%)	1 (7.7%)
Sudden cardiac arrest	0 (0.0%)	1 (14.3%)	1 (7.7%)
Heart failure	0 (0.0%)	1 (14.3%)	1 (7.7%)
Acute myocardial infarction, Sepsis of unknown origin	1 (16.7%)	0 (0.0%)	1 (7.7%)
Missing	2 (33.3%)	2 (28.6%)	4 (30.8%)
**Time between Randomisation and Death (months)**			
Mean (SD)	7.4 (1.23)	9.3 (3.87)	8.5 (3.01)
Median (IQR)	7.6 (6.5, 8.3)	9.4 (6.8, 12.2)	8.3 (6.8, 9.4)
Range	5.7, 9.1	2.6, 14.2	2.6, 14.2
Missing	0	0	0

Appendix 8: Information About Participant Safety

	STAMINA Lifestyle Intervention (n=389)	Optimised Usual Care (n=311)	Total (n=700)
**At least one Related and Unexpected Serious Adverse Event (RUSAE) reported (%)**	3 (0.8%)	0 (0.0%)	3 (0.4%)
**Number of RUSAEs reported**[4]	3	N/A	3

[1] Reported as: transient loss of consciousness; leg pain and weakness; and back pain

[1] Cardiorespiratory comorbidities may include any of the following: Asthma, Bronchitis, Any COPD, Aneurysm, Embolism, Atrial fibrillation, Stable angina, Unstable angina, Recent myocardial infarction, Pacemakers, Any other significant cardiovascular disease

[2] Musculoskeletal comorbidities may include any of the following: Arthritis (including rheumatoid), Significant musculoskeletal injury, Chronic joint/musculoskeletal complaint

[3] 11 participants were reported to have died within the 12-month trial follow-up period, with 2 occurring after 12-months.

Appendix 9: Protocol violations/deviations, researcher unblindings and CACE population

	STAMINA Lifestyle Intervention (n=389)	Optimised Usual Care (n=311)	Total (n=700)
**Protocol violation reported (%)**	0 (0.0.%)	0 (0.0%)	0 (0.0%)
**Protocol deviation reported (%)**	2 (0.5%)	0 (0.0%)	2 (0.3%)
**Reason for protocol deviation:**			
24-month visit went ahead*	1 (0.3%)	0 (0.0%)	1 (0.1%)
Referral to Nuffield Health not made†	1 (0.3%)	N/A	1 (0.1%)
**Researcher unblinding reported (%)**	12 (3.1%)	1 (0.3%)	13 (1.9%)
**Number of researcher unblindings reported**	13	1	14
Of researcher unblindings:			
**Time between Randomisation and Unblinding (months)**			
Mean (SD)	5.5 (3.46)	6.4 (.)	5.6 (3.33)
Median (IQR)	3.7 (2.2,13.2)	6.4 (6.4,6.4)	4.8 (2.2,13.2)
Range	3.1, 6.0	6.4, 6.4	3.1, 6.3
Missing	0	0	0
**Timing of unblinding relative to data collection (%)**			
Before the data collection	1 (7.7%)	1 (100.0%)	2 (14.3%)
During the data collection	7 (53.8%)	0 (0.0%)	7 (50.0%)
After the data collection	5 (38.5%)	0 (0.0%)	5 (35.7%)
**CACE population:**			
Complier‡			
Yes	276 (71.0%)	0 (0.0%)	276 (39.4%)
No	113 (29.0%)	311 (100.0%)	424 (60.6%)

Appendix 10: STAMINA Lifestyle Intervention session attendance

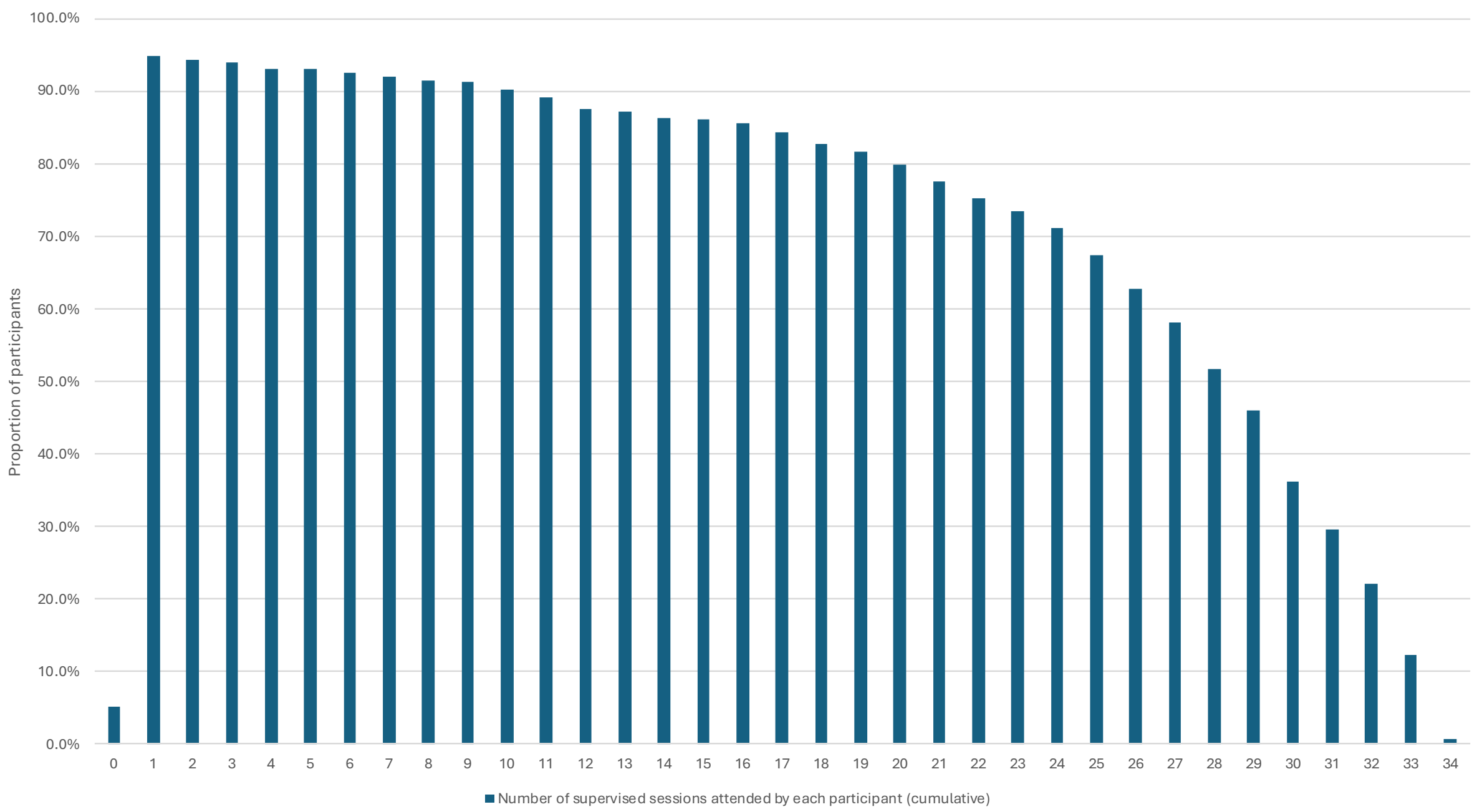

* 24-month follow-up was originally planned, however follow-up was curtailed to 12-months for all participants as part of the COVID trial extension at the request of the funder. One 24-month follow-up visit did take place for one participant.

† Participant was randomised to the STAMINA Lifestyle Intervention but was treated as Optimised Usual Care in error.

‡ Participants who completed ≥21 prescribed exercise sessions, including at least 18 during the first 12 weeks of intervention delivery.

Appendix 11: Treatment effect estimates from primary, sensitivity and exploratory analyses of the primary and secondary endpoints of FACT-P*

	N	Three Months			Six Months			Twelve Months†		
		STAMINA Lifestyle Intervention	Optimised Usual Care	Effect (97.232% CI), p-value	STAMINA Lifestyle Intervention	Optimised Usual Care	Effect (97.232% CI), p-value	STAMINA Lifestyle Intervention	Optimised Usual Care	Effect (97.232% CI), p-value
		N, Mean (97.232% CI)			N, Mean (97.232% CI)			N, Mean (97.232% CI)		
**Primary analysis**										
FACT-P	699 (3m) 698 (6m) 689 (12m)	389, 120.17 (115.96, 124.37)	310, 116.46 (112.10, 120.82)	3.71 (1.42, 5.99), p=0.00036	388, 119.00 (114.21, 123.79)	310, 115.85 (110.89, 120.80)	3.15 (0.53, 5.77), p=0.0080	383, 117.99 (112.68, 123.30)	306, 113.52 (108.10, 118.94)	4.46 (1.71, 7.22), p=0.00036
**Sensitivity analyses**										
Complete case	618 (3m) 588 (6m) 584 (12m)	351, 120.95 (116.79, 125.12)	267, 117.28 (112.97, 121.59)	3.68 (1.37, 5.99), p=0.00048	340, 120.26 (115.36, 125.15)	248, 117.22 (112.07, 122.36)	3.04 (0.36, 5.71), p=0.013	339, 119.95 (114.76, 125.14)	245, 115.51 (110.11, 120.91)	4.44 (1.63, 7.24), p=0.00052
Clustering assigned according to NH Gym‡	689 (12m)							383, 118.08 (113.35, 122.81)	306, 113.62 (108.78, 118.46)	4.46 (2.00, 6.92), p=0.00047
**Exploratory analyses**										
CACE analysis§	584									6.45 (-3.69, 16.60), p=0.16

* FACT-P = Functional Assessment of Cancer Therapy – Prostate, total scores range 0 to 156. Higher scores indicate better quality of life. Treatment effect estimate represents the adjusted mean difference between treatment groups estimated using linear mixed models, adjusted for stratification variables (with age treated as continuous), baseline score and site. A positive mean difference favours the STAMINA Lifestyle Intervention over Optimised Usual Care. Missing data imputed via multiple imputation.

† Primary endpoint timepoint

‡ Participants in the STAMINA Lifestyle Intervention arm were assigned a cluster according to the Nuffield Health gym they attended to explore the impact of a 'team effect' of intervention delivery. Analysed at 12-month timepoint only using partially nested mixed model. ICC estimate: 0.0000.

§ Estimates the effect of receiving the intervention, rather than allocation to receiving the intervention, adjusting for STAMINA Lifestyle (n=20) participants who did not receive the intervention.

Appendix 12: Treatment effect estimates from primary, sensitivity and exploratory analyses of the primary and secondary endpoints of the FACIT-F*

	N	Three Months			Six Months			Twelve Months†		
		STAMINA Lifestyle Intervention	Optimised Usual Care	Effect (97.232% CI), p-value	STAMINA Lifestyle Intervention	Optimised Usual Care	Effect (97.232% CI), p-value	STAMINA Lifestyle Intervention	Optimised Usual Care	Effect (97.232% CI), p-value
		N, Mean (97.232% CI)			N, Mean (97.232% CI)			N, Mean (97.232% CI)		
**Primary analysis**										
Primary endpoint	699 (3m) 698 (6m) 689 (12m)	389, 39.20 (36.72, 41.67)	310, 37.08 (34.53, 39.63)	2.12 (0.80, 3.44), p=0.00042	388, 37.68 (34.63, 40.73)	310, 36.17 (33.10, 39.24)	1.51 (0.04, 2.98), p=0.024	383, 38.47 (35.42, 41.51)	306, 36.58 (33.47, 39.68)	1.89 (0.35, 3.42), p=0.0068
**Sensitivity analyses**										
Complete case	624 (3m) 599 (6m) 596 (12m)	355, 39.57 (37.19, 41.96)	269, 37.54 (35.07, 40.01)	2.03 (0.71, 3.35), p=0.00072	348, 38.31 (35.54, 41.09)	251, 36.77 (33.86, 39.69)	1.54 (0.04, 3.04), p=0.024	345, 39.12 (36.22, 42.02)	251, 37.06 (34.05, 40.07)	2.06 (0.51, 3.61), p=0.0035
Clustering assigned according to NH Gym‡	689 (12m)							383, 38.49 (35.79, 41.18)	306, 36.60 (33.85, 39.36)	1.89 (0.52, 3.25), p=0.0069
**Exploratory analyses**										
CACE analysis§	596									4.52 (-0.30, 9.34), p=0.039

Additional covariate parameter estimates from primary analysis of the primary endpoints of the FACT-P and FACIT-F at 12-months

		FACT-P**		FACIT-F ††	
**Variable**	**Level**	**Mean Difference (97.232% CI)**	**p-value**	**Mean Difference (97.232% CI)**	**p-value**
Score at Baseline		0.82 (0.73, 0.91)	<.0001	0.75 (0.65, 0.85)	<.0001
Age (continuous) at Baseline		-0.11 (-0.32, 0.10)	0.26	-0.08 (-0.21, 0.05)	0.17
Duration on ADT	> 12 weeks vs. ref: ≤12 weeks	0.91 (-2.32, 4.14)	0.53	1.08 (-0.64, 2.80)	0.17
Receiving chemotherapy and/or novel ARI	No vs. ref: Yes	-0.67 (-4.47, 3.12)	0.70	0.02 (-2.05, 2.09)	0.98
Receiving radiotherapy	No vs ref: Yes	0.00 (-9.02, 9.03)	1.0	0.54 (-4.81, 5.89)	0.82
Treatment Arm	STAMINA Lifestyle Intervention vs. ref: Optimised Usual Care	4.46 (1.71, 7.22)	0.00036	1.89 (0.35, 3.42)	0.0068

* FACIT-F = Functional Assessment of Cancer Therapy – Fatigue, fatigue subscale scores range 0 to 52. Higher scores indicate lower levels of fatigue.Treatment effect estimate represents the adjusted mean difference between treatment groups estimated using linear mixed models, adjusted for stratification variables (with age treated as continuous), baseline score and site. A positive mean difference favours the STAMINA Lifestyle Intervention over Optimised Usual Care. Missing data imputed via multiple imputation.

† Primary endpoint timepoint

‡ Participants in the STAMINA Lifestyle Intervention arm were assigned a cluster according to the Nuffield Health gym they attended to explore the impact of a 'team effect' of intervention delivery. Analysed at 12-month timepoint only using partially nested mixed model. ICC estimate: 0.000.

§ Estimates the effect of receiving the intervention, rather than allocation to receiving the intervention, adjusting for STAMINA Lifestyle (n=20) participants who did not receive the intervention.

** FACT-P = Functional Assessment of Cancer Therapy – Prostate, total scores range 0 to 156. Higher scores indicate better quality of life.

†† FACIT-F = Functional Assessment of Cancer Therapy – Fatigue, fatigue subscale scores range 0 to 52. Higher scores indicate lower levels of fatigue.

Appendix 13: Physical secondary outcomes

Outcome	Baseline		Three Months		Six Months		Twelve Months	
	STAMINA Lifestyle Intervention	Optimised Usual Care	STAMINA Lifestyle Intervention	Optimised Usual Care	STAMINA Lifestyle Intervention	Optimised Usual Care	STAMINA Lifestyle Intervention	Optimised Usual Care
**Weight (kg)**								
Mean (SD)	86.4 (14.23)	86.9 (14.47)	87.8 (13.70)	88.1 (14.10)	87.6 (14.22)	87.3 (13.75)	88.1 (14.36)	87.5 (13.72)
Median (IQR)	85.0 (76.0, 95.0)	85.0 (76.2, 95.0)	86.4 (78.0, 97.2)	86.5 (78.2, 96.0)	86.0 (77.0, 96.0)	86.0 (78.0, 95.1)	86.1 (78.3, 96.0)	86.6 (78.5, 95.0)
Range	57.2, 136	55.6, 140	56.2, 138.1	58.5, 140.0	57.1, 138.4	55.6, 142.8	51.4, 141.7	59.4, 141.0
Missing	0	0	4	10	3	5	1	3
N	389	311	329	250	331	241	313	231
**Height (m)**								
Mean (SD)	1.8 (0.07)	1.8 (0.07)	1.8 (0.07)	1.8 (0.06)	1.8 (0.07)	1.8 (0.07)	1.8 (0.07)	1.8 (0.06)
Median (IQR)	1.8 (1.7, 1.8)	1.8 (1.7, 1.8)	1.8 (1.7, 1.8)	1.8 (1.7, 1.8)	1.8 (1.7, 1.8)	1.8 (1.7, 1.8)	1.8 (1.7, 1.8)	1.8 (1.7, 1.8)
Range	1.5, 2.0	1.5, 2.0	1.4, 2.0	1.6, 2.0	1.4, 2.0	1.5, 2.0	1.6, 2.01.7, 1.8	1.6, 2.0
Missing	0	0	3	10	1	5	0	5
N	389	311	330	250	333	241	314	229
**BMI (kg/m^2)**								
Mean (SD)	27.8 (4.16)	28.1 (4.20)	28.4 (4.32)	28.4 (4.20)	28.4 (4.48)	28.2 (4.18)	28.5 (4.52)	28.4 (4.02)
Median (IQR)	27.2 (24.7, 30.2)	27.7 (25.2, 30.4)	27.7 (25.2, 30.8)	28.1 (25.7, 30.8)	27.4 (25.1, 31.0)	27.8 (25.5, 30.6)	27.7 (25.4, 30.9)	28.1 (25.8, 30.8)
Range	19.0, 41.8	17.5, 44.2	20.2, 45.8	19.7, 43.9	17.6, 44.8	19.6, 44.7	17.6, 48.0	19.5, 45.3
Missing	0	0	5	11	3	7	1	5
N	389	311	328	249	331	239	313	229
**Waist circumference (cm)**								
Mean (SD)	101.7 (10.27)	102.5 (11.01)	103.5 (10.84)	103.2 (11.03)	103.5 (11.15)	102.5 (11.03)	103.1 (11.21)	102.6 (11.33)
Median (IQR)	101.0 (95.0, 107.0)	102.0 (95.0, 108.0)	102.0 (96.0, 109.5)	102.0 (96.0, 109.0)	102.0 (96.0, 111.0)	101.0 (96.0, 109.0)	102.0 (95.0, 109.5)	102.0 (95.0, 109.0)
Range	74.0, 143.0	76.0, 147.0	81.0, 143.0	74.0, 143.0	79.0, 139.0	74.0, 153.0	76.0, 147.0	76.0, 149.0
Missing	5	5	17	21	8	11	6	9
N	384	306	316	239	326	235	308	225
**Hip circumference (cm)**								
Mean (SD)	103.6 (8.42)	104.0 (9.13)	104.9 (8.68)	104.7 (8.68)	105.1 (9.16)	104.4 (8.81)	104.7 (9.01)	103.9 (9.58)
Median (IQR)	103.0 (99.0, 108.0)	103.0 (98.0, 109.0)	104.0 (99.0, 111.0)	104.0 (99.0, 110.0)	104.0 (99.0, 109.0)	104.0 (98.0, 109.0)	104.0 (99.0, 109.0)	103.0 (99.0, 109.0)
Range	81.0, 140.0	79.0, 142.0	86.0, 133.0	89.0, 141.0	86.0, 141.0	86.0, 138.0	85.0, 147.0	42.0, 142.0
Missing	6	6	22	22	11	11	6	10
N	383	305	311	238	323	235	308	224
**Chair sit to stand (reps in 30 seconds)**								
Mean (SD)	13.9 (4.10)	13.9 (4.38)	15.4 (5.13)	15.8 (5.38)	16.7 (5.55)	16.7 (5.69)	17.7 (5.97)	16.7 (6.01)
Median (IQR)	14.0 (11.0, 16.0)	13.0 (11.0, 16.0)	15.0 (12.0, 18.0)	15.0 (12.0, 19.0)	16.0 (13.0, 19.0)	16.0 (13.0, 20.0)	16.0 (14.0, 20.0)	16.0 (12.0, 20.0)
Range	3.0, 31.0	4.0, 28.0	4.0, 37.0	3.0, 34.0	5.0, 37.0	5.0, 35.0	5.0, 36.0	0.0, 40.0
Missing	3	2	19	30	20	23	20	23
N	386	309	314	230	314	223	294	211
**Blood pressure (systolic)**								
Mean (SD)	134 (14.0)	135 (15.3)	136.0 (17.11)	137.9 (16.93)	136.1 (16.52)	136.0 (15.35)	135.5 (16.46)	134.0 (15.37)
Median (IQR)	134 (125, 142)	135 (124, 144)	135.0 (125.0, 146.0)	136.0 (128.0, 149.0)	135.0 (125.0, 145.0)	135.0 (126.0, 146.0)	133.5 (123.0, 144.0)	132.0 (123.0, 142.0)
Range	98.0, 192	101, 176	92.0, 192.0	91.0, 191.0	96.0, 187.0	103.0, 184.0	98.0, 186.0	104.0, 182.0
Missing	0	0	16	15	15	9	10	11
N	389	311	317	245	319	237	304	223
**Blood pressure (diastolic)**								
Mean (SD)	77.5 (9.74)	78.1 (9.51)	78.7 (10.31)	79.5 (10.0)	78.6 (9.71)	78.9 (10.19)	78.4 (10.285)	77.8 (9.86)
Median (IQR)	78.0 (71.0, 83.0)	78.0 (71.0, 85.0)	78.0 (72.0, 86.0)	79.0 (73.0, 86.0)	78.0 (72.0, 85.0)	78.0 (72.0, 86.0)	78.0 (71.0, 85.0)	77.0 (71.0, 83.0)
Range	53.0, 116	49.0, 109	53.0, 104.0	54.0, 109.0	51.0, 109.0	46.0, 110.0	53.0, 109.0	48.0, 114.0

Missing	0	0	16	15	15	9	10	11
N	389	311	317	245	319	237	304	223

Appendix 14: Forest Plot depicting the mean difference in FACT-P at 12-months in SLI versus OUC by moderator subgroup variables*

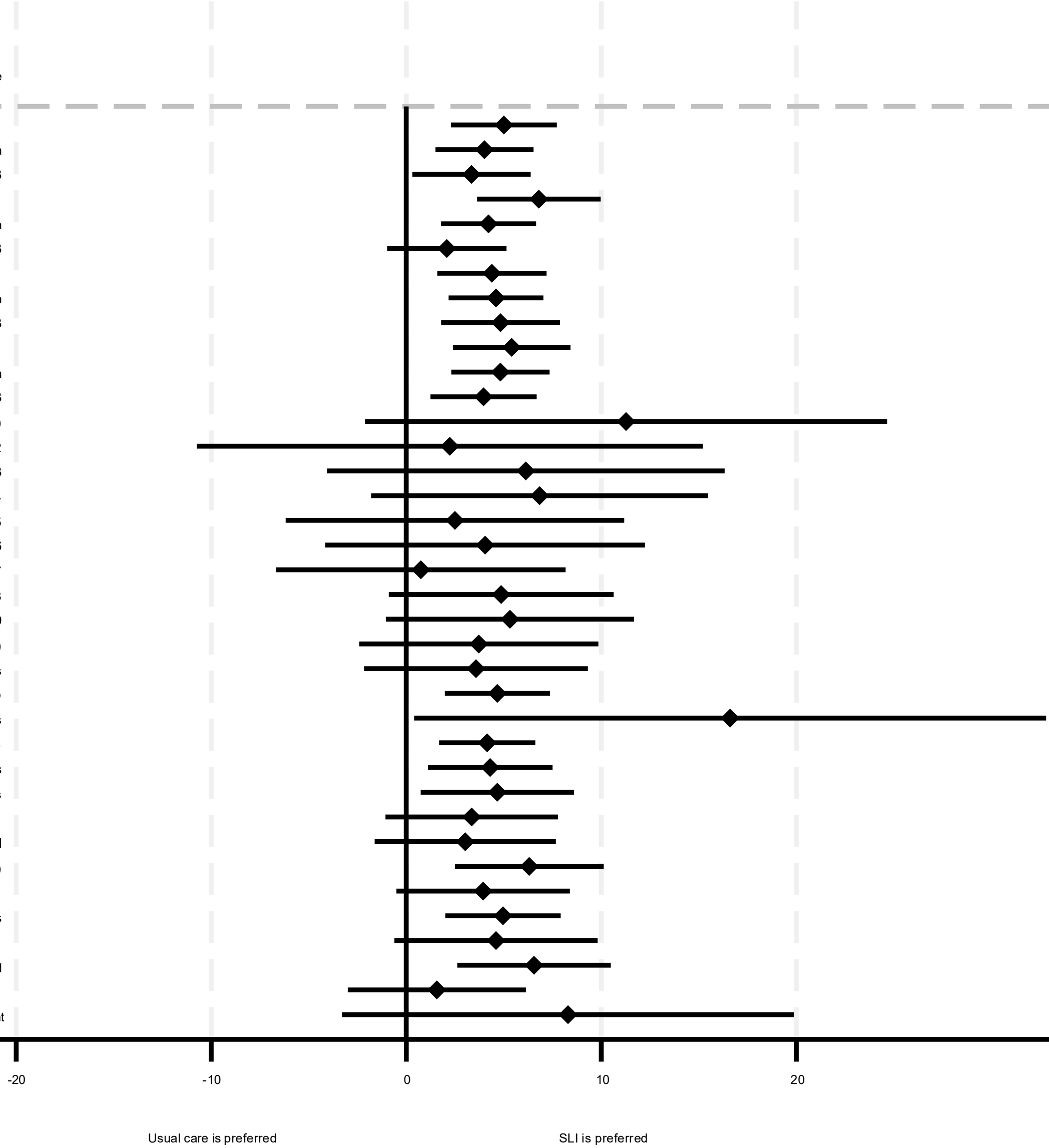

Moderator	Value	Mean difference (95% CI)		p-value for arm*moderator effect
Baseline FACT-F FS score	Q1	5.01 (2.29, 7.72)	0.00031	0.33
	Median	4.01 (1.50, 6.53)	0.0018	
	Q3	3.35 (0.32, 6.38)	0.030	
Age	Q1	6.80 (3.63, 9.97)	<0.0001	0.041
	Median	4.22 (1.78, 6.66)	0.00070	
	Q3	2.08 (-0.98, 5.14)	0.18	
ABSI Z-score	Q1	4.39 (1.59, 7.20)	0.0021	0.84
	Median	4.60 (2.17, 7.03)	0.00021	
	Q3	4.84 (1.79, 7.89)	0.0019	
Chair sit-to-stand	Q1	5.41 (2.39, 8.42)	0.00045	0.20
	Median	4.83 (2.31, 7.35)	0.00018	
	Q3	3.96 (1.24, 6.69)	0.0043	
IMD decile	1 (most deprived)	11.27 (-2.12, 24.66)	0.099	0.95
	2	2.23 (-10.74, 15.21)	0.74	
	3	6.13 (-4.07, 16.33)	0.24	
	4	6.84 (-1.80, 15.48)	0.12	
	5	2.50 (-6.18, 11.18)	0.57	
	6	4.05 (-4.15, 12.24)	0.33	
	7	0.75 (-6.67, 8.17)	0.84	
	8	4.87 (-0.90, 10.63)	0.098	
	9	5.32 (-1.05, 11.69)	0.10	
	10 (least deprived)	3.72 (-2.41, 9.85)	0.23	
Chemotherapy and/or novel androgen receptor inhibitors	Yes	3.58 (-2.16, 9.31)	0.22	0.84
	No	4.67 (1.98, 7.37)	0.00069	
Radiotherapy	Yes	16.61 (0.40, 32.81)	0.045	0.097
	No	4.15 (1.68, 6.62)	0.00099	
Time on ADT	≤ 12 weeks	4.30 (1.11, 7.50)	0.0083	0.62
	> 12 weeks	4.67 (0.74, 8.61)	0.020	
Cardiovascular comorbidities	No cardiorespiratory comorbidities	3.36 (-1.07, 7.78)	0.14	0.41
	Hypertension and/or DM	3.03 (-1.62, 7.67)	0.020	
	At least one cardiorespiratory comorbidity (+/- hypertension or DM)	6.31 (2.49, 10.12)	0.0012	
Musculoskeletal comorbidities	No musculoskeletal comorbidities	3.94 (-0.50, 8.39)	0.082	0.40
	Musculoskeletal comorbidities	4.96 (2.00, 7.92)	0.0010	
Disease status	Localised disease	4.60 (-0.60, 9.81)	0.083	0.59
	Locally advanced	6.55 (2.62, 10.49)	0.0011	
	Hormone sensitive	1.57 (-3.00, 6.14)	0.50	
	Castrate resistant	8.29 (-3.29, 19.88)	0.16	

* Presented mean differences and associated p-values are those estimated at specific values of continuous moderators (at the quartiles 1, 2, and 3) and within subgroups for categorical moderators. The p-value in the final column represents the treatment by moderator interaction effect. Moderator analyses showed no clear evidence that the effect of SLI differed between participant groups. Baseline characteristics did not appear to meaningfully influence the results, suggesting the intervention had a similar effect across the study population.

Appendix 15: Forest Plot depicting the mean difference in FACIT-F at 12-months in SLI versus OUC by moderator subgroup variables*

Moderator	Value	Mean difference (95% CI)		p-value for arm*moderator effect
Age	Q1	2.57 (0.85, 4.29)	0.0034	0.26
	Median	1.82 (0.45, 3.19)	0.0094	
	Q3	1.19 (-0.54, 2.93)	0.18	
ABSI Z-score	Q1	1.83 (0.22, 3.44)	0.026	0.85
	Median	1.97 (0.60, 3.34)	0.0048	
	Q3	2.14 (0.44, 3.83)	0.014	
Chair sit-to-stand	Q1	2.27 (0.61, 3.93)	0.0073	0.36
	Median	2.06 (0.66, 3.45)	0.0039	
	Q3	1.73 (0.22, 3.23)	0.024	
IMD decile	1 (most deprived)	4.17 (-3.17, 11.52)	0.27	0.78
	2	1.48 (-5.47, 8.42)	0.68	
	3	1.19 (-4.59, 6.96)	0.69	
	4	3.29 (-1.75, 8.33)	0.20	
	5	-0.55 (-5.31, 4.22)	0.82	
	6	-0.61 (-4.94, 3.71)	0.78	
	7	0.35 (-3.76, 4.46)	0.87	
	8	4.07 (0.72, 7.43)	0.017	
	9	3.08 (-0.35, 6.51)	0.078	
	10 (least deprived)	1.27 (-2.09, 4.63)	0.46	
Chemotherapy and/or novel androgen receptor inhibitors	Yes	0.90 (-2.38, 4.18)	0.59	0.67
	No	2.12 (0.62, 3.63)	0.0057	
Radiotherapy	Yes	6.52 (-3.08, 16.13)	0.18	0.33
	No	1.77 (0.39, 3.15)	0.012	
Time on ADT	≤ 12 weeks	1.59 (-0.21, 3.38)	0.083	0.63
	> 12 weeks	2.28 (0.18, 4.39)	0.034	
Cardiovascular comorbidities	No cardiorespiratory comorbidities	2.13 (-0.30, 4.57)	0.086	0.26
	Hypertension and/or DM	0.46 (-2.07, 3.00)	0.72	
	At least one cardiorespiratory comorbidity (+/- hypertension or DM)	2.73 (0.47, 4.98)	0.018	
Musculoskeletal comorbidities	No musculoskeletal comorbidities	2.97 (0.48, 5.45)	0.019	0.38
	Musculoskeletal comorbidities	1.57 (-0.12, 3.25)	0.068	
Disease status	Localised disease	1.65 (-1.21, 4.51)	0.26	0.033
	Locally advanced	3.84 (1.65, 6.03)	0.00061	
	Hormone sensitive	-0.74 (-3.31, 1.83)	0.57	
	Castrate resistant	4.09 (-2.63, 10.82)	0.23	

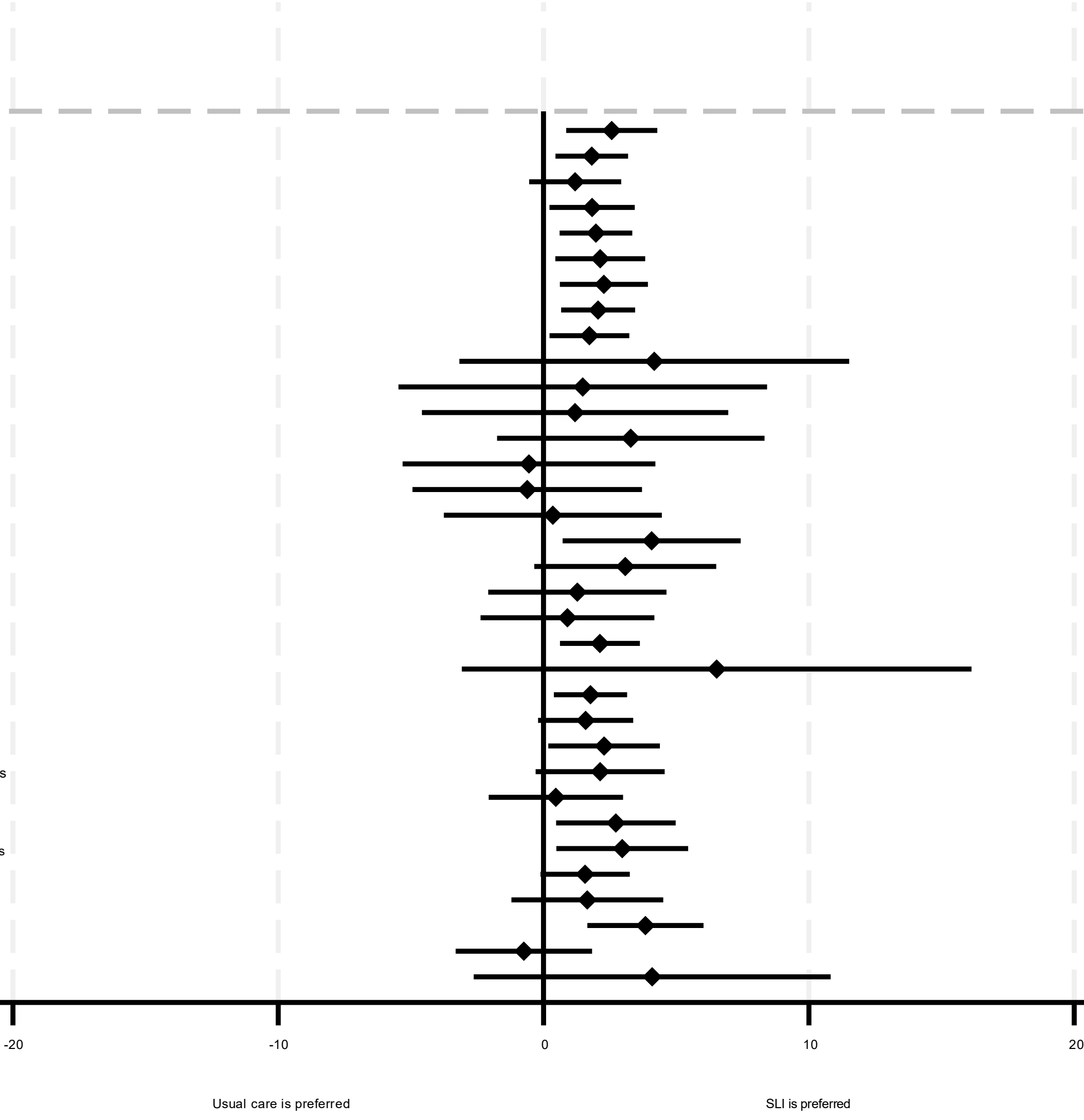

* Presented mean differences and associated p-values are those estimated at specific values of continuous moderators (at the quartiles 1, 2, and 3) and within subgroups for categorical moderators. The p-value in the final column represents the treatment by moderator interaction effect. Moderator analyses showed no clear evidence that the effect of SLI differed between participant groups. Baseline characteristics did not appear to meaningfully influence the results, suggesting the intervention had a similar effect across the study population.

Appendix 16: Mediation analysis for the association between FACT-P and FACIT-F scores at 12-months*

Mediators	FACT-P	FACIT-F
**ABSI z-score at 3-months, N**	485	495
Natural direct association	4.23 (1.25 to 7.21)	2.07 (0.36 to 3.79)
Natural indirect association	0.06 (-0.17 to 0.29)	0.01 (-0.11 to 0.12)
Proportion mediated (%)	1.4% (-3.9% to 6.8%)	0.3% (-5.1% to 5.8%)
**ABSI z-score at 6-months, N**	506	516
Natural direct association	3.59 (0.67 to 6.51)	1.90 (0.23 to 3.57)
Natural indirect association	0.07 (-0.16 to 0.31)	0.01 (-0.13 to 0.14)
Proportion mediated (%)	2.0% (-4.5% to 8.6%)	0.4% (-6.9% to 7.6%)
**Godin score at 3-months, N**	545	556
Natural direct association	4.53 (1.66 to 7.40)	2.13 (0.54 to 3.73)
Natural indirect association	0.08 (-0.45 to 0.62)	0.08 (-0.17 to 0.33)
Proportion mediated (%)	1.8% (-9.6% to 13.3%)	3.6% (-8.0% to 15.2%)
**Godin score at 6-months, N**	536	546
Natural direct association	3.96 (1.04 to 6.87)	2.07 (0.46 to 3.68)
Natural indirect association	0.19 (-0.23 to 0.62)	0.15 (-0.08 to 0.38)
Proportion mediated (%)	4.7% (-5.9% to 15.2%)	6.7% (-4.5% to 17.9%)
**FACIT-F score at 6-months, N**	557	N/A
Natural direct association	3.32 (0.65 to 5.99)	
Natural indirect association	0.82 (-0.12 to 1.77)	
Proportion mediated (%)	19.9% (-1.6% to 41.3%)	

Mediation analyses showed that only a small proportion of the treatment effect was explained by the measured intermediates, with most of the effects appearing to operate through direct pathways.

* All mediators were modelled as continuous variables.

Appendix 17. Cost-effectiveness acceptability curves

Cost-effectiveness acceptability curve (CEAC) – Base Case, Scenario 1

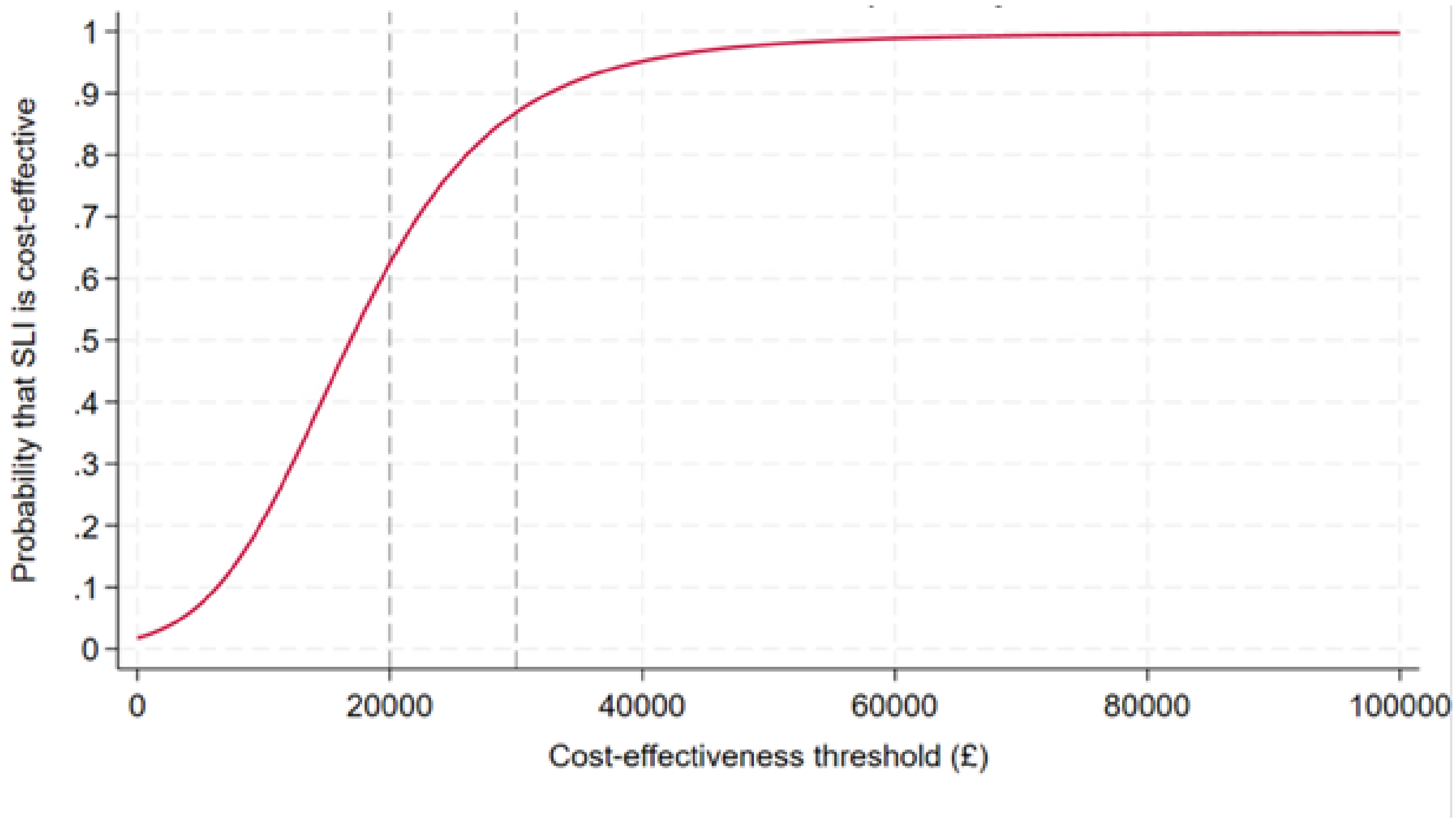

Cost-effectiveness acceptability curve (CEAC) – Base Case, Scenario 2

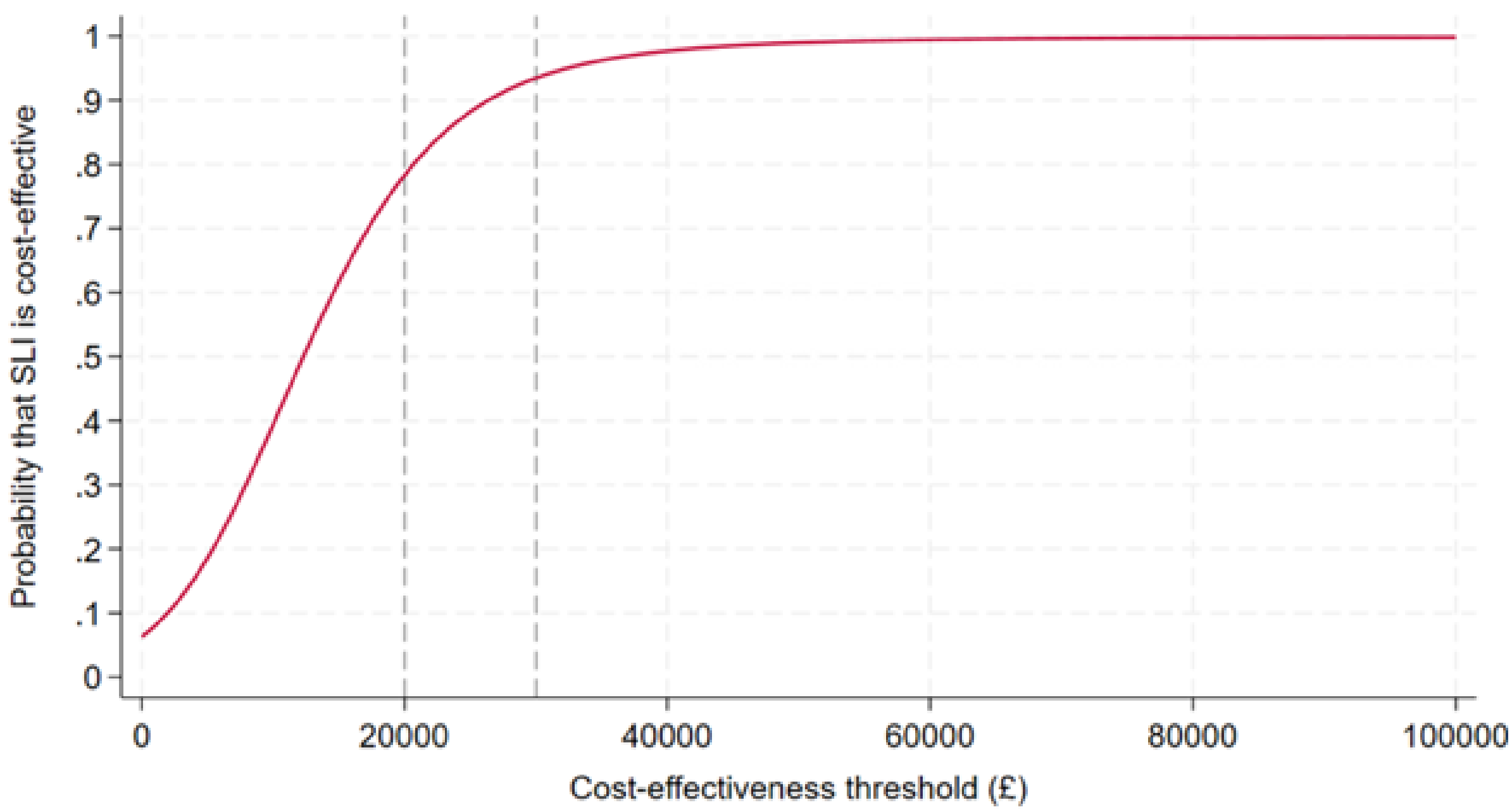

Appendix 18. Economic evaluation: Decision Modelling methods

A decision model was developed[1,2] to estimate the long-term cost-effectiveness of SLI given that benefits may extend beyond the end of the trial follow-up period. The model structure (See Figure 1 and Table 1) was informed by literature, trial data and consultation with a clinical expert. The model included health states defined by physical activity level (e.g. active, moderately active, insufficiently active) based on the Godin questionnaire categories. Transition probabilities, costs, and utilities were based on analysis of the trial data and supplemented with secondary data. The 12-month distribution of patients across the health states was determined from the trial data at end of follow-up. Health state cost and utility values were derived using mixed effects repeated measures analysis of the trial data. We used data from a published study in ADT to estimate background survival in this group. This was done by digitizing Kaplan-Meier data from the trial publication and fitting several parametric survival curves. On the basis of visual fit and plausibility of long-term predictions, a Gompertz survival curve was fitted to the data. This was applied to the STAMINA cohort and published hazard ratios for survival relating to physical activity levels was applied to determine transitions to the dead state. Some adjustment was required such that the activity categories better matched Godin categories. Survival expectations from the trial (mean age 66) were adjusted to match the starting age of the STAMINA trial cohort (mean age 72 at 12 months).

We applied multinomial logit models to the Godin category levels over time in the trial data (from 3-12 months in OUC; and from 6-12 months in SLI) to determine transition probabilities and trends in activity levels. However, no statistically significant changes in categories over time were observed indicating activity levels (including maintenance of activity in the SLI arm) were relatively static. Given this, and with a view to retaining simplicity in the modelling, we did not model transitions between Godin categories beyond 12 months.

Although free gym membership ended at 12 months in the SLI arm, personal communication from Nuffield indicated that around 70% of men had chosen to continue gym membership beyond trial end. However, to capture potential waning of activity in the SLI arm, the activity health state proportions in the SLI arm was adjusted such that by year 5, they matched the distribution in the OUC arm. The initial time horizon planned for the decision modelling was over a lifetime. However, given the uncertainty in maintenance in behaviours, the lack of follow-up in SLI beyond the end of free gym membership and acknowledging the mean age of the group, the analysis period was shortened to 10 years. Furthermore, since waning of effectiveness was applied at 5 years, there is little differential benefit captured beyond this point.

The adjusted total costs and QALYs at 12 months from the trial (and associated uncertainty) was incorporated, and subsequently future costs, survival and HRQoL in annual cycles was modelled, with costs and outcomes beyond one year being discounted at 3.5% per annum.

A probabilistic sensitivity analysis (PSA) was run where Monte Carlo simulations were used to draw (n=1,000) parameter values from distributions to reflect uncertainty in the data. The simulated estimates of cost-effectiveness were used to estimate the probability of cost-effectiveness. The model was developed in R.

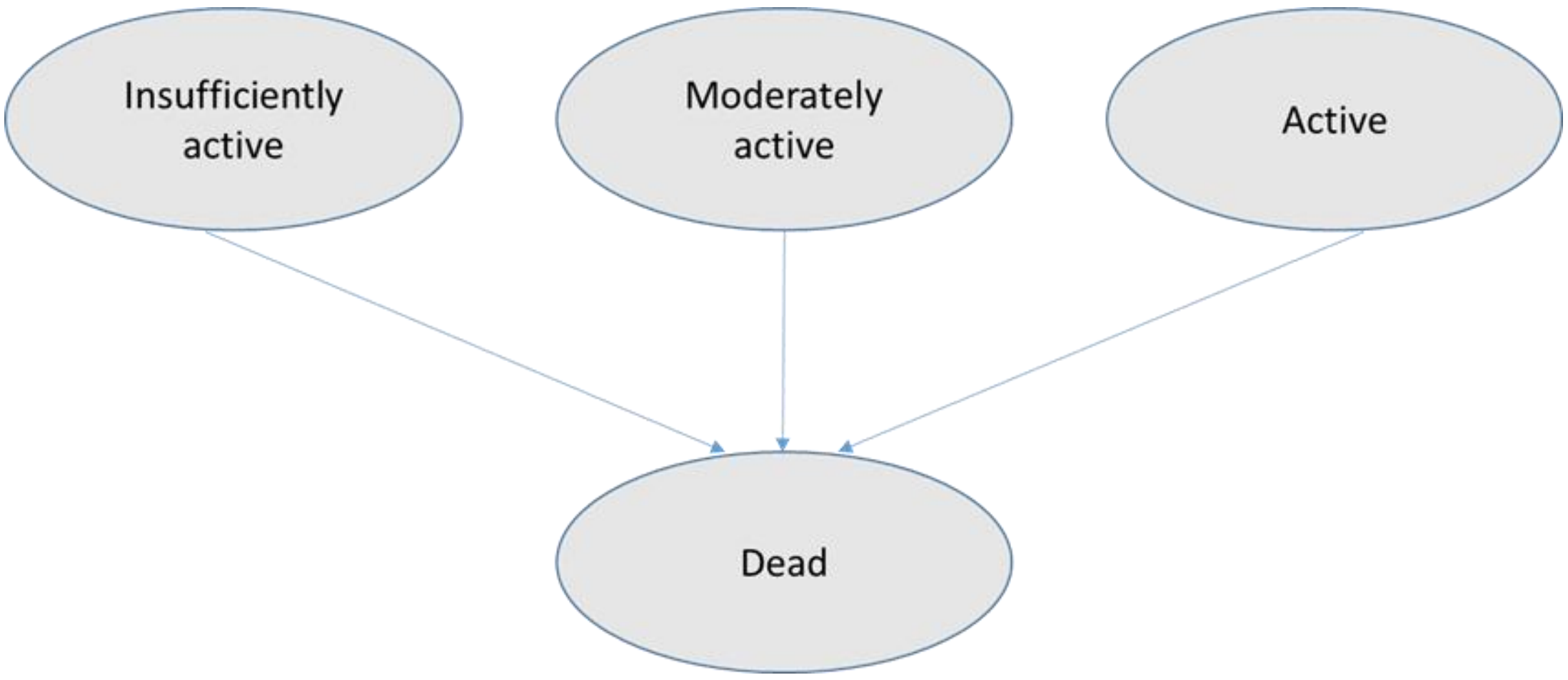

Figure: Decision model diagram

Table: Decision model parameter values

Parameter	Arm	Value	PSA Distribution	Parameter 1	Parameter 2	Source
Threshold	Both	£20,000	N/A			NICE Manual
Cohort start age	Both	72	N/A			STAMINA Trial
Discount rate - QALYs	Both	0.035	N/A			NICE Manual
Discount rate: Costs	Both	0.035	N/A			NICE Manual
**Health state distribution: 12 months:**			All - Dirichlet			STAMINA Trial
Insufficiently active	OUC	19.5%				
Moderately active	OUC	21.2%				
Active	OUC	56.4%				
Dead	OUC	2.8%				
Insufficiently active	SLI	12.9%				
Moderately active	SLI	16.1%				
Active	SLI	69.2%				
Dead	SLI	1.8%				
**Background mortality**	Both		Gompertz	0.0065	0.1827	Parker et al. (2024)
**Activity-related mortality HRs:**			Normal	SD		Friedenreich et al.(2016)
Insufficiently active	Both	1.0		N/A	N/A	
Moderately active	Both	0.77		0.20		SD assumed
Active	Both	0.62		0.20		SD assumed
**Costs-Resource use (per year):**				SE		STAMINA Trial
Insufficiently active	Both	£1209.59	Gamma	88.74		
Moderately active	Both	£1041.50	Gamma	68.47		
Active	Both	£999.53	Gamma	42.52		
Dead	Both	£2,170.00	Fixed			NICE TA3920 (Olaparib with abiraterone)
**Utility**:				SE		STAMINA Trial
Insufficiently active	Both	0.756	Beta	0.008		
Moderately active	Both	0.783	Beta	0.007		
Active	Both	0.805	Beta	0.006		

Appendix 18 references

1. Friedenreich CM, Wang Q, Neilson HK, Kopciuk KA, McGregor SE, Courneya KS. Physical Activity and Survival After Prostate Cancer. Eur Urol. 2016 Oct;70(4):576-585.

2. Parker CC, Clarke NW, Cook AD et al. Randomised Trial of No, Short-term, or Long-term Androgen Deprivation Therapy with Postoperative Radiotherapy After Radical Prostatectomy: Results from the Three-way Comparison of RADICALS-HD (NCT00541047). Eur Urol. 2024 Nov;86(5):422-430

Appendix 19: Recruiting sites, Principal Investigators and numbers of participants recruited per site

Site	Principal Investigator	Number of participants recruited
University Hospitals Plymouth NHS Trust	Ms Bea Hamilton	143
University Hospital Coventry and Warwickshire NHS Trust	Mr Kieran Jefferson	97
Sheffield Teaching Hospitals NHS Foundation Trust	Mr Marcus Cumberbatch	90
Surrey and Sussex Healthcare NHS Trust	Dr Chee Goh	70
Chesterfield Royal Hospital NHS Trust	Ms Kate Linton	48
Nottingham University Hospitals NHS Trust	Claire Brotherton	45
Cambridge University Hospital NHS Foundation Trust	Mr Kasra Saeb-Parsy	38
Gloucestershire Hospitals NHS Foundation Trust	Ms Kim Davenport	36
Hull University Teaching Hospitals NHS Foundation Trust	Mr Matt Simms	36
Stockport NHS Foundation Trust	Mr James Dyer	28
Liverpool University Hospitals NHS Foundation Trust	Mr Philip Cornford	18
Clatterbridge Cancer Centre NHS Foundation Trust	Michelle Cain	16
University Hospitals of Derby and Burton NHS Trust	Mr Simon Williams	15
Yeovil District Hospital NHS Foundation Trust	Mr Tim Porter	12
Bradford Teaching Hospitals NHS Foundation Trust	Mr Chidi Molokwu	8

Appendix 20 Acknowledgements and STAMINA study group members

Acknowledgements

The trial was funded by the NIHR Programme Grant for Applied Research panel (RP-PG-1016-20007). Nuffield Health provided 12-month memberships to patients randomised to SLI and provided staff to be upskilled to CES providers. This work acknowledges the support of the National Institute for Health Research Barts Biomedical Research Centre (NIHR203330).

The STAMINA study group members include the site Principal Investigators, development grant collaborators and key staff at the Leeds CTRU

Claire Brotherton - Nottingham University Hospitals NHS Trust

Michelle Cain – Clatterbridge Cancer Centre NHS Foundation Trust

Philip Cornford – Liverpool University Hospitals NHS Foundation Trust

Marcus Cumberbatch – Sheffield Teaching Hospitals NHS Foundation Trust

Kim Davenport – Gloucestershire Hospitals NHS Foundation Trust

James Dyer – Stockport NHS Foundation Trust

Chee Goh – Surrey and Sussex Healthcare NHS Trust

Bea Hamilton – University Hospitals Plymouth NHS Trust

Kieran Jefferson – University Hospital Coventry and Warwickshire NHS Trust

Kate Linton – Chesterfield Royal Hospital NHS Trust

Chidi Molokwu – Bradford Teaching Hospitals NHS Foundation Trust

Tim Porter – Yeovil District Hospital NHS Foundation Trust

Kasra Saeb-Parsy – Cambridge University Hospital NHS Foundation Trust

Matt Simms – Hull University Teaching Hospitals NHS Foundation Trust

Simon Williams – University Hospitals of Derby and Burton NHS Trust

Rebecca Turner – Manchester University

Suzanne Hartley – Data Access service, NHS England

Emma McNaught – Leeds Institute of Clinical Trials Research

Florence Day – Leeds Institute of Clinical Trials Research

Chris Taylor – Leeds Institute of Clinical Trials Research

Geoffrey Wong – University of Oxford

Abi Fisher – University College London

Nanette Muttrie, University of Edinburgh

Noel Clarke, The Christie NHS foundation trust and Salford Royal Hospital

Joe Sullivan, Northern Ireland Cancer Centre and Queen’s University Belfast

Claire Hulme, University of Exeter

Julia Brown, University of Leeds

Raj Persad, North Bristol NHS Trust

Amit Bahl, Bristol Haematology & Oncology Centre (BHOC), University Hospitals Bristol NHS Trust

McNaught et al. Trials (2024) 25:257
https://doi.org/10.1186/s13063-024-07989-y

Trials

STUDY PROTOCOL

Open Access

Supported exercise TrAining for Men wIth prostate caNcer on Androgen deprivation therapy (STAMINA): study protocol for a randomised controlled trial of the clinical and cost-effectiveness of the STAMINA lifestyle intervention compared with optimised usual care, including internal pilot and parallel process evaluation

Emma McNaught[1†], Sophie Reale[2†], Liam Bourke[2], Janet E. Brown[3], Michelle Collinson[1], Florence Day[1], Jenny Hewison[4], Amanda J. Farrin[1], Saïd Ibeggazene[2], Aidan Q. Innes[5], Ellen Mason[1], David Meads[6], Alison Scope[2], Chris Taylor[1], Steph JC. Taylor[7], Rebecca R. Turner[8], Derek J. Rosario[9*†] and on behalf of the STAMINA co-investigators

Abstract

Background UK national clinical guidance recommends that men with prostate cancer on androgen deprivation therapy are offered twice weekly supervised aerobic and resistance exercise to address iatrogenic harm caused by treatment. Very few NHS trusts have established adequate provision of such services. Furthermore, interventions fail to demonstrate sustained behaviour change. The STAMINA lifestyle intervention offers a system-level change to clinical care delivery addressing barriers to long-term behaviour change and implementation of new prostate cancer care pathways. This trial aims to establish whether STAMINA is clinically and cost-effective in improving cancer-specific quality of life and/or reducing fatigue compared to optimised usual care. The process evaluation aims to inform the interpretation of results and, if the intervention is shown to benefit patients, to inform the implementation of the intervention into the NHS.

Methods Men with prostate cancer on androgen deprivation therapy ($n = 697$) will be identified from a minimum of 12 UK NHS trusts to participate in a multi-centre, two-arm, individually randomised controlled trial. Consenting men

†Emma McNaught and Sophie Reale are the lead authors.

†Derek J. Rosario is the senior author.

*Correspondence:
Derek J. Rosario
d.j.rosario@sheffield.ac.uk
Full list of author information is available at the end of the article

© The Author(s) 2024. **Open Access** This article is licensed under a Creative Commons Attribution 4.0 International License, which permits use, sharing, adaptation, distribution and reproduction in any medium or format, as long as you give appropriate credit to the original author(s) and the source, provide a link to the Creative Commons licence, and indicate if changes were made. The images or other third party material in this article are included in the article's Creative Commons licence, unless indicated otherwise in a credit line to the material. If material is not included in the article's Creative Commons licence and your intended use is not permitted by statutory regulation or exceeds the permitted use, you will need to obtain permission directly from the copyright holder. To view a copy of this licence, visit http://creativecommons.org/licenses/by/4.0/. The Creative Commons Public Domain Dedication waiver (http://creativecommons.org/publicdomain/zero/1.0/) applies to the data made available in this article, unless otherwise stated in a credit line to the data.

will have a 'safety to exercise' check and be randomly allocated (5:4) to the STAMINA lifestyle intervention ($n = 384$) or optimised usual care ($n = 313$). Outcomes will be collected at baseline, 3-, 6- and 12-month post-randomisation. The two primary outcomes are cancer-specific quality of life and fatigue. The parallel process evaluation will follow a mixed-methods approach to explore recruitment and aspects of the intervention including, reach, fidelity, acceptability, and implementation. An economic evaluation will estimate the cost-effectiveness of the STAMINA lifestyle intervention versus optimised usual care and a discrete choice experiment will explore patient preferences.

Discussion The STAMINA lifestyle intervention has the potential to improve quality of life and reduce fatigue in men on androgen deprivation therapy for prostate cancer. Embedding supervised exercise into prostate cancer care may also support long-term positive behaviour change and reduce adverse events caused by treatment. Findings will inform future clinical care and could provide a blueprint for the integration of supervised exercise and behavioural support into other cancer and/or clinical services.

Trial registration ISRCTN 46385239, registered on 30/07/2020. Cancer Research UK 17002, retrospectively registered on 24/08/2022.

Keywords Prostate cancer, Supervised exercise, Lifestyle intervention, Behaviour change, Complex intervention, Healthcare professionals, Exercise professionals, Randomised controlled trial, Process evaluation, Health economics

Administrative information

Title {1}	*S*upported exercise *TrA*ining for *M*en w*I*th prostate ca*N*cer on *A*ndrogen deprivation therapy – (STAMINA): study protocol for a randomised controlled trial of the clinical and cost-effectiveness of the STAMINA lifestyle intervention compared with optimised usual care, including internal pilot and parallel process evaluation
Trial registration {2a and 2b}	ISRCTN 46385239 (30/07/2020), Cancer Research UK 17002 (24/08/2022 – retrospectively registered)
Protocol version {3}	Protocol v10.0 05/07/2023
Funding {4}	NIHR Programme Grants for Applied Research (PGfAR) RP-PG-1016–20007
Author details {5a}	Emma McNaught[1] (E.McNaught@leeds.ac.uk), Sophie Reale[2] (S.Reale@shu.ac.uk), Liam Bourke[2] (L.Bourke@shu.ac.uk), Janet E Brown[3] (j.e.brown@sheffield.ac.uk), Michelle Collinson[1] (M.Collinson@leeds.ac.uk), Florence Day[1] (F.Day1@leeds.ac.uk), Jenny Hewison[4] (j.hewison@leeds.ac.uk), Amanda J. Farrin[1] (A.J.Farrin@leeds.ac.uk), Saïd Ibeggazene[2] (S.Ibeggazene@shu.ac.uk), Aidan Q Innes[5] (Aidan.Innes@nuffieldhealth.com), Ellen Mason[1] (e-mason@live.com), David Meads[6] (D.Meads@leeds.ac.uk), Alison Scope[2] (Alison.Scope@shu.ac.uk), Chris Taylor[1] (C.M.Taylor@leeds.ac.uk), Steph JC Taylor[7] (S.J.C.Taylor@qmul.ac.uk), Rebecca R Turner[8](Rebecca.Turner@manchester.ac.uk), Derek J Rosario[9] (D.J.Rosario@Sheffield.ac.uk) [1]Clinical Trials Research Unit, Leeds Institute of Clinical Trials Research, University of Leeds, Leeds, LS2 9JT [2]Dept. of Allied Health Professions, Sheffield Hallam University, Sheffield, S10 2BP [3]Division of Clinical Medicine, University of Sheffield, Sheffield, S10 2RX [4]Division of Health Services Research, Leeds Institute of Health Sciences, University of Leeds, Leeds, LS2 9JT [5]Nuffield Health, 2 Ashley Avenue, Epsom, Surrey, KT18 5AL [6]Academic Unit of Health Economics, Leeds Institute of Health Sciences, University of Leeds, Leeds, LS2 9JT [7]Wolfson institute of Population Health, Queen Mary University of London, Yvonne Carter Building, 58 Turner Street, London, E1 2AB [8]Division of Psychology and Mental Health in the School of Health Sciences, The University of Manchester, Manchester, M13 9PL [9]Sheffield Teaching Hospitals NHS Foundation Trust, Department of Urology, Royal Hallamshire Hospital, Glossop Road, Sheffield, S10 2JF
Name and contact information for the trial sponsor {5b}	Sheffield Teaching Hospitals NHS Foundation Trust: Clinical Research Office 0114 226 5943
Role of sponsor {5c}	The Sponsor had no direct input into the design or conduct of the trial and accepts no responsibility for the accuracy of content reproduced into documentation developed by collaborating or third-party organisations The funder had no role in trial design

Introduction

Background and rationale {6a}

Prostate cancer is common and responsible for a quarter of new male cancer diagnoses in the UK [1], with around 50% being locally advanced or metastatic at presentation. The prevalence of prostate cancer is likely to continue rising as a result of an ageing population, increased screening and advancements in treatment. However, for many men living with and beyond prostate cancer, persistent adverse effects from treatment are common, often debilitating and can be experienced lifelong [2].

Androgen deprivation therapy (ADT) remains the mainstay of treatment in prostate cancer as sole treatment in men unfit for any other treatment, combined with radiotherapy for locally advanced disease [3] and in conjunction with taxane-based chemotherapy or novel androgenic signalling inhibitors (e.g. abiraterone, enzalutamide, apalutamide and daralutamide or, in fitter patients, an androgenic signalling inhibitor in combination with taxane-based chemotherapy) for metastatic disease [4]. ADT is effective for prostate cancer and men can remain on ADT for up to two decades [5, 6]. However, ADT is associated with significant adverse events including hot flushes, fatigue, bone fracture risk, diabetes, cognitive dysfunction, depression, and sexual dysfunction [7, 8]. For example, 12 weeks of ADT have been shown to increase body fat by 5% and reduce lean body mass, predisposing men towards sarcopenia [9]. Furthermore, abdominal obesity, hyperglycaemia, and an increased risk of cardiovascular disease have been observed in men receiving ADT [10–13]. Subsequently, the impact of ADT adverse events on quality of life (QoL) [14] and healthcare/treatment costs [15] is increasingly recognised.

To date, exercise training is the only evidence-based intervention to demonstrate clinically relevant beneficial effects on fatigue and disease-specific QoL for men receiving ADT for prostate cancer [16]. Exercise is also associated with improvements in cardiovascular health and potential benefits for mental health for men on ADT [17, 18]. Consequently, UK (the National Institute for Health and Care Excellence (NICE) NG 131 1.4.19 [19]) and international (European Association of Urology; EAU [20]) guidelines recommend 12 weeks of combined resistance and aerobic exercise as standard treatment for people on ADT. However, the UK National Health Service (NHS) provision of such treatment is almost non-existent [21]. Furthermore, established short-term benefits of supervised exercise dissipate without ongoing support demonstrating the significant challenges associated with maintaining behaviour change [22].

The STAMINA lifestyle intervention (SLI) is a behaviourally informed supervised exercise programme with dietary support developed using intervention development methodology [23] and underpinned by evidence from an earlier programme development grant [21]. Healthcare professionals (HCPs) will be trained to endorse and refer men to SLI and provide ongoing behavioural support whilst undergoing ADT [24]. Supervised exercise will be delivered by trained exercise professionals (known as Clinical Exercise Specialists (CESs)) from Nuffield Health (NH); a UK healthcare charity that invests heavily in staff training and routinely delivers exercise and behavioural support to various clinical populations.

The SLI has demonstrated good feasibility, acceptability, fidelity, and safety when embedded into routine clinical practice with delivery partner NH during a feasibility study [25]. The STAMINA model could offer an improved standard of care for men with prostate cancer and provide a blueprint for the integration of supervised exercise and behavioural support into other cancer or clinical services. However, the long-term clinical and cost-effectiveness of the intervention is currently unknown.

Objectives {7}

Primary objective

To examine the effect of SLI on cancer-specific QoL measured using the Functional Assessment of Cancer Therapy – Prostate (FACT-P [26]) or on cancer-specific fatigue measured using the Functional Assessment of Chronic Illness Therapy – Fatigue (FACIT-F [27]), both at 12-month post-randomisation.

Secondary objectives

- To establish whether the intervention promotes physical, social, emotional, and functional wellbeing at 3-, 6- and 12-month post-randomisation using FACT-P [26] subdomains.
- To establish whether the intervention reduces cancer-specific fatigue at 3- and 6-month post-randomisation using FACIT-F [27].
- To establish whether the intervention increases leisure time physical activity, measured using the Godin [28] questionnaire at 3-, 6-, and 12-month post-randomisation.
- To establish whether the intervention reduces fear of cancer recurrence measured using Fear of Cancer Recurrence (FCR4 and FCR7) [29] at 3-, 6- and 12-month post-randomisation.
- To establish whether the intervention improves functional capacity and body composition, measured using blood pressure, chair sit-to-stand, waist and hip circumference and weight at 3-, 6-, and 12-month post-randomisation.

- To establish related and unexpected serious adverse event (RUSAE) rates, and severity.
- To establish whether the SLI reduces adverse effects, commonly associated with ADT measured using a trial-specific ADT symptom index at 3-, 6- and 12-month post-randomisation.
- To establish participants' perceptions of capabilities, opportunities, and motivations to perform a target behaviour using the Capability, Opportunity, Motivation-Behaviour (COM-B) [30] Questionnaire.
- To establish whether the intervention is cost-effective, assessed using incremental cost-effectiveness ratios (ICERs). The outcome of interest is cost per incremental quality-adjusted life year (QALY) at 12-month post-randomisation with QALYs being derived from the EuroQol 5 Domains 5 Levels (EQ-5D-5L) [31].
- To establish patient preferences and to predict demand for alternative extended exercise programme features using a discrete choice experiment (DCE) [32].
- To establish moderator and mediator variables which influence engagement with and benefit from treatment.

Internal pilot objectives

To assess rates of recruitment, 3-month follow-up, and adherence to the intervention, after 12 months of participant recruitment, against pre-defined progression criteria.

Process evaluation objectives

- To understand trial recruitment performance.
- To describe intervention reach, dose delivered, and dose received.
- To describe the fidelity of intervention delivery.
- To understand how the intervention was experienced and understood by patients using semi-structured interviews (underpinned by consideration of the Theoretical Framework of Acceptability).
- To explore the organisational implications of embedding and sustaining the intervention in preparation for wider NHS roll-out (underpinned by consideration of Normalization Process Theory).

Trial design {8}

The STAMINA trial is a multi-centre, two-arm, individually randomised controlled trial (RCT) with a two-level partially nested design, an internal pilot with clear progression criteria, a cost-effectiveness analysis and a parallel mixed-methods process evaluation. Six hundred and ninety-seven participants will be randomly allocated with a 5:4 ratio to either SLI or optimised usual care (OUC).

Methods: participants, interventions and outcomes

Study setting {9}

Recruitment will take place across England at a minimum of 12 NHS Trusts (listed in the 'Acknowledgements' section). Potential trial participants will be approached to take part by secondary care clinical or research teams, and those willing and potentially eligible will be referred onto the Sheffield Hallam University research team for consent and baseline data collection. The SLI will be delivered in NH fitness and wellbeing centres (listed in the 'Acknowledgements' section) by CESs. Participants in both trial arms will receive OUC provided by STAMINA-trained secondary care NHS HCPs.

Eligibility criteria {10}

Trial sites

NHS sites that previously expressed an interest in participating in the STAMINA programme as part of earlier work will be approached by the trial team and assessed for eligibility. A feasibility questionnaire will determine if sites have services appropriate to support recruitment, delivery of OUC, and support for trial-related research activity (e.g. data collection). Eligible sites include secondary or tertiary care hospitals with a urology diagnostic centre or a cancer treatment centre where men on ADT have their allocated consultant, that:

- Have at least one NH fitness and wellbeing centre located within reasonable travelling distance in the locality
- Agree to keep an ADT log to facilitate audit of the men's treatment
- Identify the care team involved in delivering care to men with prostate cancer and make them available for training, and
- Agree to usual care for men on ADT eligible to exercise to become the STAMINA OUC arm.

Sites already delivering an embedded exercise intervention that is considered superior to OUC will be excluded.

Participants

Patients meeting all the following criteria (and none of the exclusion criteria) prior to randomisation will be eligible to take part:

Inclusion criteria

- Men with prostate cancer on ADT or due to start ADT within the next 12 weeks.[1]
- Willing to provide informed consent.

Exclusion criteria

- Absolute contraindication to exercise as defined by clinical guidance, e.g. ACPICR standards [33];
- Uncontrolled hypertension;
- Uncontrolled diabetes mellitus;
- Recent myocardial infarction (within the past 6 months);
- Unable to provide informed consent (e.g. lack of capacity);
- Unstable bony metastases unresponsive to treatment;
- Unable to complete study assessments;
- Participation in other lifestyle intervention trials for prostate cancer;
- Estimated life expectancy of less than 12 months for reasons unrelated to prostate cancer diagnosis; and
- Involvement in previous STAMINA work packages or patient and public involvement (PPI) panel.

Participation in another study will not necessarily exclude a patient from participation. The feasibility of co-enrolment will be reviewed, considering methodological impact and participant burden.

Documented reasons for ineligibility or declining participation will be monitored by the Leeds Clinical Trials Research Unit (CTRU) as part of a regular review of recruitment progress.

Who will take informed consent? {26a}

Clinical staff at site will be required to complete an ADT log for men with prostate cancer receiving ADT within their diagnostic unit. HCPs will gain verbal consent from initially eligible patients for referral to the research team at Sheffield Hallam University (SHU) for a 'safety to exercise' check. Any uncertainties around suitability for exercise will be checked against participant's medical records. The potential participant will be given written information about the trial, and the opportunity to ask any questions or raise any queries about participation. A trained member of the research team at SHU will obtain participant consent by telephone for trial participation and data collection. If the potential participant chooses to consent, the patient will be registered onto the trial. Following confirmation of eligibility and completion of baseline assessments the registered participant will be randomised to their treatment allocation.

Withdrawal of consent

Withdrawal from, or non-attendance for, the intervention are not classed as withdrawal from the trial, and all follow-up will continue as planned, unless a participant specifically expresses a wish to withdraw from trial processes. Clarification will be sought on whether withdrawal is from participation in the intervention, questionnaire completion or ongoing access to health records and data processing.

The right of a participant to refuse participation without giving reasons will be accepted. The participant will remain free to withdraw at any time from the trial without giving reasons and without prejudicing their further treatment. If participants of the proposed trial withdraw consent from further participation, their data collected up to that point will be included in the final trial analysis. This will be made clear to the participants at the time of consent and when they withdraw from the trial.

Additional consent provisions for collection and use of participant data and biological specimens {26b}

Participant consent for information collected as part of the STAMINA trial to be shared for use in future research is optional in the original consent. Participant consent to wear a heart rate monitor and upload their anonymised data onto Garmin is optional in the original consent for SLI participants.

Interventions

Explanation for the choice of comparators {6b}

Optimised usual care was developed based on a) findings from the programme development grant and early work packages [21], exploring variations in NHS prostate cancer care pathways and exercise provision for men on ADT and b) from the SHU research teams' experience of providing written guidance for control groups in cancer and lifestyle research [34].

Intervention description {11a}

Optimised usual care (OUC)

All participants will receive OUC (including SLI participants). OUC includes endorsement of the NICE guidelines around the benefits of exercise for men on ADT and behavioural support (where appropriate) from key

[1] Where a man is due to start Taxane based chemotherapy in conjunction with ADT, an initial health questionnaire will be conducted but inclusion in the trial will be deferred until after completion of the course of chemotherapy (typically 18–20 weeks). Baseline assessments will be repeated before randomisation where necessary.

workers during routine clinic appointments. Consenting participants will then undergo a 'safety to exercise' check administered over the phone by researchers at Sheffield Hallam University, with support from clinical teams, principal investigators, and the chief investigator where clinical queries arise. Participants will be sent an information pack through the post including a booklet about the NICE guidelines for people on ADT and local and nationally available resources related to lifestyle (e.g. information materials from Prostate Cancer UK (PCUK) [35], Macmillan [36], Cancer Research UK (CRUK) [37]).

The healthcare professional intervention

NHS prostate cancer clinical teams will receive a behaviorally informed, bespoke training package and continuous intervention support during recruitment to the STAMINA trial (see Additional file 1) [24]. Training will last up to 3 h and will be delivered face-to-face or remotely depending on local policy and preference. The first training session will be delivered during site set-up and will be centred on four R's: *R*ecognizing who is suitable for the STAMINA trial, *R*ecommending exercise in line with NICE guidance, *R*eferral to the STAMINA trial and *R*ecording research related information on the ADT log. Follow-up training will be delivered at 6–12 weeks following initiation of recruitment and will focus on supporting lifestyle behaviour change during routine clinic follow-up appointments. In parallel, ad hoc intervention support will be offered and regular feedback on referral performance will be provided via email. Full details of intervention development have been published [24].

The STAMINA lifestyle intervention (SLI)

SLI was developed in line with two overarching principles: (1) SLI will be offered as an intrinsic part of a man's cancer care as opposed to an 'add-on' and (2) the intervention will be designed in a way to maximise both efficacy (likelihood of change) and effectiveness (accessibility and generalisability beyond urban areas).

SLI is a behaviourally informed, lifestyle intervention embedded into prostate cancer care to support delivery of NICE (NG131 1.4.19) guidance for people with prostate cancer on ADT (see Additional file 1). The SLI is focussed on supervised exercise training (primarily face-to-face) combining resistance and aerobic exercise with application of the behavioural skills learnt to other patient-negotiated lifestyle goals, including dietary modification. The SLI will be delivered in partnership with NH, a community-based, appropriately trained exercise provider, and will only be available to participants randomised to the intervention arm for 12 months.

SLI participants will be invited to an induction to exercise with a CES to explore their capability, opportunity, and motivation to exercise twice a week for 12 months. The supervised exercise sessions will include both aerobic (30–45 min) and resistance (up to 4 sets, 8–12 reps of major muscle groups) components as recommended by NICE (NG131 1.4.19 [19]) and in accordance with exercise programmes previously shown to be beneficial and safe to men on ADT (e.g. Bourke et al., 2014 [16]). The supervised exercise programme will be tailored to address individual requirements and will be delivered twice weekly for the first 12 weeks, initially one-to-one before transition to small groups (based on satisfactory progress). For the remaining 9 months of the programme, negotiated supervision will be offered (i.e. once a month maximum, once every 3 months minimum).

In parallel to supervised exercise sessions, behaviour change support will be delivered during programme reviews and catch-up calls between the CES and participant, and by completion of a behaviourally informed STAMINA diary. Programme reviews will be scheduled at 12 weeks, 6 months and 12 months following the induction session and will include a review of participant progress and a written summary report. Summary reports will be sent to the referring clinical team via email as part of the feedback loop and for discussion with the participant at their next routine clinic appointment. Catch-ups (face-to-face or over the phone) will also be organised with the CES and participant every 6 weeks to explore participant progress and address any barriers to intervention adherence (where applicable).

During the 12-month intervention period, SLI participants will be provided with a complimentary NH gym membership to support additional, independent exercise behaviour. SLI participants will also be offered one reduced price membership (~50%) for a family member to further support their exercise behaviour by addressing social support.

The exercise professional intervention

Community-based CESs and gym management/operational/front-of-house staff will receive a behaviourally informed, role-specific, bespoke training package with ongoing intervention support during delivery of the SLI (see Additional file 1). Level 1 training will comprise three online modules providing high-level information about prostate cancer, the SLI, patient confidentiality and operational procedures. All management, operational, sales, fitness and front-of-house staff will be invited to complete the online modules with a mandatory 80% pass mark. Staff responsible for the delivery of SLI (i.e. fitness managers, rehab specialists and personal trainers) will be invited to level 2 training which targets knowledge, confidence and skill related to delivering supervised exercise and behavioural support to

men with prostate cancer. Level 2 training will be delivered face-to-face on site at NH over one full day. Ongoing tapered intervention support will be offered in the form of a weekly telephone call for up to 4 weeks followed by a monthly meeting with all CESs involved in the delivery of SLI. Full details of intervention development have been published [38].

Criteria for discontinuing or modifying allocated interventions {11b}

The risks of the supervised exercise programme in men on ADT are minimal, with no increase in the risk of serious adverse events reported in a systematic review of RCTs evaluating the delivery of such programmes in these men [22]. All recruited men will continue to be under the care of their treating cancer clinician, who will be aware of their participation in the trial. In line with standard clinical care, cessation of the SLI at any time will be at the discretion of the clinicians or the participants themselves. Pauses to the programme will be accommodated for health-related reasons, holidays (up to 2 weeks maximum) and modification will be considered for individual cases (e.g. to remote sessions).

Strategies to improve adherence to interventions {11c}

Intervention adherence will be monitored weekly by the research team at SHU by reviewing SLI participants' attendance at supervised exercise sessions, to identify those who have dipped below 75% attendance. CESs at each NH site will upload data relating to attendance and completion of aerobic and resistance exercise components of the SLI after each session using a REDCap web-based reporting software. Researchers will download, compile, and clean the data on a weekly basis. The database will track adherence relative to the number of prescribed sessions to date and produce "alerts" once a SLI participant drops below 75% adherence. This alert with suggested actions will be sent onto the NH fitness manager who will identify and deliver behavioural support in line with the COM-B [30] model of behaviour change (i.e. data verification, identify required behavioural support and deliver behavioural support). If a participant's attendance remains below 75%, a maximum two further alerts are sent. A record of all alerts sent and subsequent actions will be maintained.

Relevant concomitant care permitted or prohibited during the trial {11d}

The protocol does not restrict participant access to usual care services. The usual care delivered at each site is documented at the point of site inclusion in the trial, at 12 months after the site opening and at 12 months after the last participant at the site is recruited. Participants also self-report their usual care received at 3-, 6- and 12-month post-randomisation. Should the trial team become aware of any new referrals to services, the nature of the referral will be established and discontinuation in the SLI considered.

Provisions for post-trial care {30}

Should participants disclose anything to the trial team which puts them or anyone else at risk, the trial team may feel it necessary to report this to the appropriate persons. Additional exercise support outside of the SLI is not provided by CESs, and no specific aftercare is planned as part of the trial.

The NHS Sponsor is a member of the Clinical Negligence Scheme for Trusts (CNST) which provides indemnity cover for clinical negligent harm only. The Sponsor has an agreement in place with NH and therefore CNST indemnity extends to cover this service provider and their staff who have a duty of care to research participants.

Outcomes {12}

Outcome data will be collected via participant self-report postal/online questionnaires and follow-up assessments with a local research nurse, or delegate, at 3-, 6- and 12-month post-randomisation. Supported completion of questionnaires by site staff can be requested by participants. If supported follow-up is required, wherever possible a research nurse, or delegate, who is blind to the participant's allocation will offer telephone follow-up to participants who are unable to complete assessments independently. If site staff are subsequently unblinded, additional and subsequent data collection will be completed by an alternative researcher who is blinded to allocation.

Primary outcomes

Disease-specific QoL and fatigue at 12-month post-randomisation were measured by FACT-P [26] and FACIT-F [27]. The FACT-P [26] incorporates primary QOL domains: Physical Well-Being, Social/Family Well-Being, Emotional Well-Being, and Functional Well-Being with the addition of a prostate cancer-specific subscale. The FACIT-F [27] assesses perceptions of fatigue over the last 7 days.

Secondary outcomes

Secondary endpoints will be measured at 3-, 6- and 12-month post-randomisation and are as follows:

Questionnaire outcomes

- Physical, Social/Family, Emotional and Function wellbeing, assessed using FACT-P [26] subdomains
- Cancer-specific fatigue (FACIT-F [27].)
- Leisure time physical activity assessed using the Godin [28] questionnaire.
- Fear of recurrence and psychological distress markers assessed using FCR4 [29] and FCR7 [29].
- Adverse effects of ADT assessed using trial specific ADT Symptom Index.
- Perceptions of capabilities, opportunities and motivations to perform a target behaviour assessed by the COM-B [30] Questionnaire.

Physical measures

Functional capacity and body composition assessed by blood pressure, chair sit-to-stand, waist and hip circumference and weight.

Safety

- Adverse events assessed by:

Number and proportion of RUSAEs
Number of RUSAEs per participant
Details of RUSAEs including severity
Number and proportion of deaths
Details of deaths including primary cause and timing

Health economics

- Generic quality of life assessed using EQ-5D-5L [31] questionnaire
- Costs of the intervention and of health care resource use
- Cost per incremental QALY using EQ-5D-5L [31] questionnaire
- Patient preferences and willingness to pay for exercise programmes as assessed by the DCE [32].

Moderator/mediators Moderator and mediator variables which influence engagement with and benefit from the intervention will also be measured.

Participant timeline {13}

The trial timeline for participants is presented in Fig. 1, outlining the schedule of enrolment and interventions, and in Fig. 2, outlining the schedule of assessments.

Sample size {14}

A total sample size of 697 men (313 OUC, 384 SLI) is required to provide 90% power to detect a small to moderate effect size of either 0.33 in FACT-P [26] (8 point difference) or 0.35 in FACIT-F [27] (3 point difference) at the 5% significance level. Calculations assume a maximum standard deviation of 24 (FACT-P [26]) and 8.6 (FACIT-F [27]) and 30% loss to follow-up by 12 months [38]. Clustering at the level of the CES in the intervention arm is accounted for using an intracluster correlation coefficient (ICC) of 0.05 [39], a coefficient of variation of 0.6 to account for variation in the number of men each CES will deliver the intervention to, an average cluster size of 6 participants (range 1 to 14) per CES and 64 CES.

The sample size calculation takes into account the correlation between the two primary endpoints based on methodology by Micheaux et al. [40]. A correlation between FACT-P [26] and FACIT-F [27] of 0.7 (95% confidence internal (CI): 0.58–0.78) was observed at baseline in the pilot data [16]. The lower bound of the CI for the correlation estimate (0.58) was incorporated into the calculations. After accounting for multiple testing with correlated continuous multiple primary endpoints, the adjusted alpha level is 0.02768.

Recruitment {15}

To ensure recruitment of 697 men within an 18-month period, the internal pilot green progression criteria for recruitment was set as at least 4 men per site per month from a minimum of 12 NHS Trusts based on a minimum of 40 men from each site, per year, over a total recruitment period of 18 months. Additional sites will be added during the recruitment period to deliver the overall sample size of 697 if required.

Assignment of interventions: allocation

Sequence generation {16a}

Participants will be randomised on a 5:4 basis to receive either SLI or OUC. A computer-generated minimisation programme incorporating a random element will be used to ensure arms are well-balanced for the following stratification factors:

- Age: <70 years OR ≥ 70 years
- Duration on ADT: ≤ 12 weeks OR >12 weeks
- Receiving chemotherapy and/or novel androgen receptor inhibitors: yes OR no
- Receiving radiotherapy: yes OR no

Concealment mechanism {16b}

Randomisation will be performed using the Leeds CTRU automated 24-h randomisation service, which can be

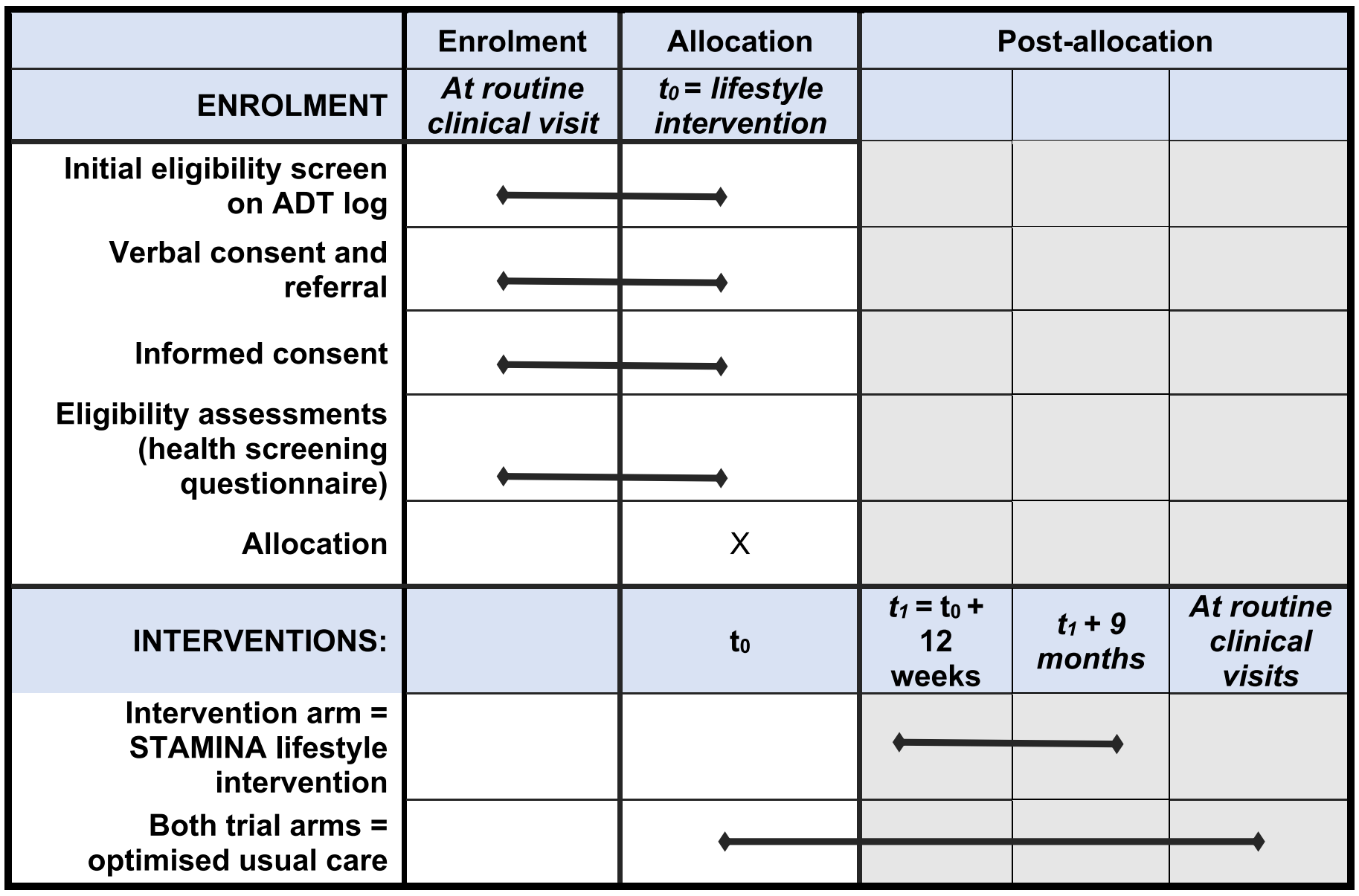

Fig. 1 Schedule of enrolment and interventions

accessed via the web and ensures allocation concealment. Personal usernames and passwords required to access the randomisation system will be provided to sites when all relevant trial approvals are in place.

Implementation {16c}

Following participant consent, confirmation of eligibility and collection of baseline data, a member of the SHU research team will randomise the participant. The principal investigator and relevant local research team at the site will receive an automated email confirmation of successful randomisation, highlighting subsequent tasks required and omitting randomisation allocation details. Participants will be contacted via letter/email to confirm their randomisation allocation. The research team at SHU will refer participants randomised to SLI to NH to initiate the exercise programme.

Assignment of interventions: blinding

Who will be blinded {17a}

Participants and NH staff will be aware of treatment allocation, but researchers involved in data collection, general practitioners (GP) and clinical teams will be blind to the allocation. Analysis will be conducted unblind to allocation according to pre-specified analysis plans.

It is not possible to blind participants or those involved in the delivery of the intervention in studies evaluating exercise/lifestyle interventions.

CTRU will regularly monitor for bias by reviewing participant characteristics (e.g. stratification factors, number of comorbidities and other characteristics as deemed appropriate) by the treatment arm to check for imbalances. CTRU and the trial management group (TMG) will monitor instances of unblinding to review for systematic errors that could impact upon trial integrity and elevate concerns to the programme steering committee (PSC).

Procedure for unblinding if needed {17b}

Trial participation will be recorded in participants' hospital notes and with their GP. This will not reveal allocation but will detail where further information can be gained if required. A central list of participants' randomisation allocations will be maintained at SHU and at CTRU. We do not foresee a situation where allocation needs to be revealed outside of regular office hours.

Data collection and management

Plans for assessment and collection of outcomes {18a}

The schedule for participant assessments is summarised in Fig. 2: Schedule of assessments.

Baseline data for consenting patients will be collected by the research team at SHU from the participant directly. The local researcher at the participating site will collect data from medical records (e.g. blood pressure). Participants will self-complete all remaining assessments. Where inclusion in the trial is deferred until after completion of a course of chemotherapy, baseline assessments will be repeated before randomisation.

At 3-, 6- and 12-month post-randomisation, follow-up questionnaires will be completed by participants online or by post. Supported follow-up by site staff can

ASSESSMENTS	*Baseline*	*3 months*	*6 months*	*12 months*
Participant contact details	X			
Participant demographics	X			
FACT-P	X	X	X	X
FACIT-F	X	X	X	X
EQ-5D-5L	X	X	X	X
Godin	X	X	X	X
FCR4 & FCR7	X	X	X	X
ADT Symptom Index	X	X	X	X
Health Service Use	X	X	X	X
Exercise Preference Survey				X
Blood Pressure	X	X	X	X
Height & Weight	X	X	X	X
Chair Sit to Stand	X	X	X	X
Waist & Hip Measurements	X	X	X	X
Changes in prostate cancer treatment		X	X	X
Related and Unexpected Serious Adverse events	←———	———	———	———→
**OUC DATA**		***Baseline***	***12 months after site opening***	***12 months after last patient recruited at site***
Usual care delivered at site		X	X	X
**INTERVENTION DATA**		**Referral**	**Intervention period**	**Post intervention period**
Nuffield Health referral form		X		
Exercise CRF tailoring information		X		
Staff training attendance record			At intervention training sessions for HCP and CES	
Staff pre- and post-training forms			At intervention training sessions for HCP and CES	
Submaximal exercise test			1st visit to Nuffield Health and at 3-, 6- and 12-months	
Complete exercise prescription			1st visit to Nuffield Health	
Nuffield Health exercise record			At each visit to Nuffield Health gym	
Behaviour change (COM-B)			Baseline, 3-, 6- and 12-months	
Exercise reviews			Updated as appropriate by CES based on patient progress	
STAMINA summary report			At 3-, 6- and 12-months progress is documented and communicated to NHS HCP for review and any relevant clinical updates	
**PROCESS EVALUATION DATA**		**During follow-up**	**Intervention period**	**Post intervention period**
OUC participant interviews		X		
SLI participant interviews		X		
Interviews or focus groups with CESs			Must have delivered at least 12 weeks of supervised exercise to five or more participants	
Interviews or focus groups with HCPs			Must have completed all required training	

Fig. 2 Schedule of assessments

be requested by participants. If questionnaires are not returned on time, up to three reminders may be sent via post, email, or text depending on the participants' preferred method of contact. Where relevant, local researchers at participating sites will be asked by CTRU to confirm survival status and current address.

At 3-, 6- and 12-month post-randomisation, local researchers at participating sites will collect data from participants directly and from their medical records (e.g. blood pressure, changes in prostate cancer treatment). Any RUSAEs will also be collected.

Local researchers at participating sites will receive training on the completion of all trial-specific assessments as part of trial initiation, to ensure standardised completion. CTRU will chase missing and discrepant data as appropriate. Data collection forms are available on request at ctru-dataaccess@leeds.ac.uk.

Plans to promote participant retention and complete follow-up {18b}

Initial/reminder letters, texts and emails will be used to maximise questionnaire data return at all timepoints. Participants may request support in the completion of questionnaires by site staff or the trial team in advance or during routine telephone reminders. The TMG will monitor questionnaire return rates overall and by the treatment arm.

A postcard from the PPI group will be sent to participants at 11 months to remind them their 12-month questionnaire will arrive soon and to encourage engagement with the project. A poster from the PPI group will also be sent to participants alongside their 12-month questionnaire to provide thanks, encouragement to complete the final questionnaire, and to remind participants how to receive the study results.

Participants will remain free to withdraw at any time from the trial without giving reasons and without prejudicing their further treatment. If a participant withdraws consent to participate, clarification will be sought on whether withdrawal is because of participation in the intervention, questionnaire completion or ongoing access to health records and data processing. Data collected up to the point of withdrawal will be included in the final trial analysis. This will be made clear to the participants at the time of consent and when they withdraw from the trial.

Data management {19}

All data collection forms transferred to or from the CTRU will be coded with the participant's trial number, initials and site code. Data will be held securely on paper and electronically at CTRU, Sheffield Hallam University and at NH. All relevant Standard Operating Procedures, Guidelines and Work Instructions in relation to data management, processing and analysis of data will be followed. CTRU will provide sites with an electronic file to safely maintain essential trial documentation. Interviews for the process evaluation will be audio recorded and professionally transcribed, with any identifiable information removed. Audio files will be securely transferred in encrypted format and securely stored at Queen Mary University London, Sheffield Hallam University and Bristol.

Confidentiality {27}

All information collected during the trial will be kept strictly confidential, complying with all aspects of the 2018 Data Protection Act [41]. Appropriate storage, restricted access and disposal arrangements of personal and clinical details of participants will be put in place. At the end of the trial, sites will archive all trial data until written permission for confidential destruction is provided by the Sponsor. The Trial Master File and documents held by the CTRU will be archived at a secure facility at the University of Leeds.

Plans for collection, laboratory evaluation and storage of biological specimens for genetic or molecular analysis in this trial/future use {33}

Not applicable; no samples were collected.

Statistical methods

Statistical methods for primary and secondary outcomes {20a}

A detailed Statistical Analysis Plan will be written before any analyses are undertaken. Final analysis will be conducted once all available outcome data is received. All analyses will be conducted on the intention-to-treat (ITT) population, with all participants included in the analysis according to their randomisation allocation, and regardless of non-adherence to the intervention or withdrawal from the trial. Blinded interim reports will be presented to the PSC containing descriptive summaries of recruitment, follow-up, safety and data quality.

Primary endpoint analysis

Primary analysis will compare mean FACT-P [26] and FACIT-F [27] scores at 12-month post-randomisation between the trial arms using partially nested mixed-effects linear regression models to account for clustering of outcomes in the intervention arm due to the nesting of participants within CESs [42]. The models will be adjusted for the stratification factors and other participant-level covariates expected a priori to be associated with the outcome of interest. Each endpoint will be tested against the calculated adjusted alpha level 0.02768.

If a statistically significant result is observed in either FACT-P [26] or FACIT-F [27], the SLI will be deemed clinically different to OUC. Results will be expressed as estimated mean differences with 97.232% confidence intervals, *p*-values and ICCs. Model diagnostics will be visually assessed to check the underlying assumptions of the model and alternative methodology will be used if required.

Secondary endpoint analysis

Summary statistics will be presented for each time point by arm for secondary outcomes FACT-P [26] (overall and domain-specific), FACIT-F [27], leisure time physical activity (Godin [28]), fear of recurrence (FCR4 [29] and FCR7 [29]), adverse effects of ADT (ADT Symptom Index), functional capacity and body composition (blood pressure, chair sit-to-stand, waist and hip circumference and weight). Means, standard deviations, medians, minimum, maximum, quartiles and ranges will be presented for continuous variables and frequencies and percentages for categorical variables. Secondary endpoints will be analysed using the same approach as for the primary outcome with the relevant model for the type of outcome variable. Safety endpoints will be analysed descriptively between arms and no formal statistical comparisons will be made.

Moderators and mediators will be identified. Whether the treatment effect differs depending on the pre-specified baseline characteristics of the participant will be explored.

Internal pilot

Descriptive analysis of the internal pilot against progression criteria (Table 1) together with an examination of the sample size assumptions will take place after 12 months of recruitment.

Interim analyses {21b}

No formal interim analyses of outcomes will be undertaken.

Methods for additional analyses (e.g. subgroup analyses) {20b}

No sub-group analyses are planned.

Methods in analysis to handle protocol non-adherence and any statistical methods to handle missing data {20c}

We will explore missing data patterns and reasons for missingness to guide the assumptions around missing data. If it can be assumed that data is missing at random (MAR), the primary ITT analysis will use multiple imputation, enabling inclusion of all randomised participants. If data cannot be assumed MAR we will explore other more complex methods for the primary analysis such as pattern mixture modelling.

Plans to give access to the full protocol, participant-level data and statistical code {31c}

Anonymised data supporting this work are available on reasonable request. All requests will be reviewed by relevant stakeholders, based on the principles of a controlled access approach. Requests to access data should be made to CTRU-DataAccess@leeds.ac.uk in the first instance.

Economic evaluation

The economic evaluation will use data at 12 months and will adopt the NICE reference case employing a primary endpoint of cost per incremental QALY [43]. The primary perspective will be the health and personal social service provider, but will include supplementary analyses incorporating patient/caregiver costs (e.g. out-of-pocket expenses in attending exercise sessions).

Costs will include the costs of the interventions, costs of any additional exercise equipment and exercise facility access purchased or provided and any additional healthcare resources used (including primary and secondary care). Unit costs will be taken from the Personal Social Services Research Unit report and NHS Reference costs. Health outcomes will be assessed by the EQ-5D-5L [31] at follow-up but we will also explore basing utility on the FACT-P [26] and/or FACIT-F [27] measures (via mapping) depending on the availability of suitable mapping algorithms or direct valuation tariffs.

A Health economic analysis plan (HEAP) will be developed prior to analysis. The HEAP will be reviewed by the project team and an external health economist. The primary analysis will adopt an ITT approach but with additional pre-specified analyses conducted to evaluate the impact of intervention compliance on cost-effectiveness.

We will generate ICERs per QALY at 3, 6 and 12 months. We will use seemingly unrelated regression to account for the correlation between costs and QALYs and within this use relevant covariates to adjust for any imbalance across arms. We will use robust standard errors to account for any potential clustering in outcomes by CES in the intervention arm. The analytical process will also incorporate the strategy for handling missing data (e.g. multiple imputation, if appropriate) and the variance–covariance matrix used to generate 10,000 ICERs. The latter simulations will be plotted on the cost-effectiveness plane and help generate the cost-effectiveness acceptability curve to characterise the level of sampling uncertainty in the analysis.

Table 1 Progression criteria

Decision Criterion	Go (green) Continue the trial with no modifications	Modify (amber) Continue the trial with modifications	Stop (red) Stop the trial
**Recruitment (1,2,3)** Average number of participants randomised / site / month	≥4	<4 and ≥ 2	<2
**Follow-up at 3 months (4)** % participants completing 3m outcomes	≥75%	50-74%	<50%
**12 weeks (patient) intervention adherence** % participants completing 75% scheduled SLI exercise sessions (within 18 weeks of randomisation)	≥75%	50-74%	<50%

[1] Data from August 2022 onwards will be used for analysis of this criterion to allow for a sufficient number of sites to be open and to ensure the trial has reached a steady rate of recruitment

[2] This target is set at a gym level. Therefore, for sites with 2 gyms, the criteria are green ≥ 8, amber < 8 and ≥ 4, and red < 4

[3] Based on 12 recruiting sites

[4] FACT-P [26] and FACIT-F [27] only

A decision-analytic model will be developed to extrapolate costs and benefits over a lifetime horizon following consultation with clinical experts and patients. It will likely be a Markov Model. We will use trial data and targeted literature reviews to derive the model parameters and explore using resources such as the National Prostate Cancer Audit. We will generate lifetime ICERs for SLI vs. OUC. We will assume a cost-effectiveness threshold of £20,000 per QALY gained. We will generate net monetary benefit (NMB) and conduct extensive one-way and scenario deterministic sensitivity analyses. We will explore, the impact of different intervention costs, engagement and effectiveness decay over time on the estimates of cost-effectiveness. A probabilistic sensitivity analysis will capture the total parameter uncertainty in the model. Results from this will be presented in the form of cost-effectiveness planes, NMB distributions and cost-effectiveness acceptability frontiers [44].

We will use information from the discrete choice experiment on men's willingness to pay and likely uptake for extended (and alternative) programmes to explore alternative funding strategies. These will be incorporated in supplementary analysis exploring the cost-effectiveness of alternative programmes.

Process evaluation

The mixed methods process evaluation will be undertaken based on the Medical Research Council (MRC) guidance [45] for process evaluations and informed by the framework of Linnan and Steckler [46]. Quantitative and qualitative data will be collected to address five objectives. Data will be collected throughout the whole trial period to explore different stages of the intervention/trial. Further detail will be presented in a separate process evaluation protocol manuscript (*in preparation*).

Oversight and monitoring

Composition of the coordinating centre and trial steering committee {5d}

The coordinating centre (SHU) is responsible for intervention training and undertaking individual participant research activities, including consent, eligibility and data collection. The CTRU at the University of Leeds is responsible for set up, implementation and monitoring of trial conduct; data management and statistical design, analysis and reporting and is where the trial and data management and statistics teams are based.

The PSC will provide overall supervision of the trial and are responsible for monitoring trial progress and

providing public, clinical, and professional advice. It will include an Independent Chair, at least two other independent members, and a PPI representative. The Committee will meet annually as a minimum.

The programme management group (PMG) oversees the STAMINA Programme Grant and includes the chief investigator, co-applicants and co-investigators. The PMG will oversee the whole programme of studies and meet twice annually as a minimum.

The TMG includes the chief investigator, trial and process evaluation leads, key co-applicants, the CTRU delivery team, the SHU research team, a PPI representative and other key external members of staff involved in the trial. The TMG will oversee trial set-up; on-going management; promotion of the trial; and the interpretation and publishing of the results. The TMG will meet quarterly as a minimum.

Composition of the data monitoring committee, its role and reporting structure {21a}

For a trial of this nature, a separate Data Monitoring and Ethics Committee is not required. Rather, the PSC will adopt a safety function, with the constitution of a subcommittee to review safety issues, where necessary.

Summary safety data will be provided to the PSC to determine patterns and trends of events or to identify safety issues, which would not be apparent on an individual case basis.

Adverse event reporting and harms {22}

Adverse events fulfilling the definition of related and unexpected are reportable in this trial (resulting from the administration of any of the research procedures). All related and unexpected serious adverse events will be reviewed by the principal investigator and reported to CTRU within 24 h of becoming aware. They will be subject to expedited reporting to the Sponsor and the Research Ethics Committee (REC) within 15 days. Where the principal investigator is unable to decide whether or not the serious adverse event is either 'related' or 'unexpected', the event should be reported to the chief investigator, who will liaise with the coordinating centre and CTRU in arriving at a decision. Events will be followed up until the event has been resolved or a final outcome has been reached.

Frequency and plans for auditing trial conduct {23}

Investigators are required to promptly notify the CTRU if there is a breach of the protocol or of the conditions or principles of Good Clinical Practice which is likely to affect to a significant degree the safety or physical or mental integrity of the trial subjects, or the scientific value of the research.

The CTRU/Sponsor (or delegate) or regulatory authority reserves the right to conduct intermittent source data verification on a sample of participants. Source data verification will involve direct access to patient notes at the participating hospitals, and other relevant investigation reports.

Plans for communicating important protocol amendments to relevant parties (e.g. trial participants, ethical committees) {25}

Protocol amendments will be processed in line with REC and Health Research Authority (HRA) guidelines. Investigators will be notified and asked to confirm ongoing capacity and capability.

Dissemination plans {31a}

Trial results will be disseminated nationally and internationally to extend reach to participants, HCPs, CESs, academic researchers, the public and other key stakeholders on completion of the trial. Dissemination activities include participation in conferences, publication in peer-reviewed journals, delivery of webinars and organisation of PPI events. To maintain the scientific integrity of the trial, data will not be released prior to the end of the trial, either for publication or oral presentation purposes, without the permission of the PSC or the chief investigator and trial leads.

Discussion

STAMINA is a multi-centre, two-arm RCT examining the clinical and cost-effectiveness of the SLI compared to OUC at a 12-month follow-up. In addition, a mixed methods process evaluation and health economic evaluation will be conducted.

Men on ADT for prostate cancer experience persistent adverse effects from treatment that are often debilitating and can be experienced lifelong [2]. To date, supervised exercise is the only evidence-based intervention to address the harms caused by treatment, but previous research has failed to demonstrate sustained clinical benefit and behaviour change maintenance [22]. Moreover, NHS provision of supervised exercise, as recommended nationally and internationally, is almost non-existent due to behavioural and implementation barriers [21]. Therefore, a major opportunity exists to address this unmet need and to improve the QoL and reduce fatigue of men on ADT for prostate cancer through STAMINA.

The SLI is a behaviourally informed complex intervention designed with methodological rigour. The intervention elements are underpinned by behaviour change theory, implementation science and evidence to optimise

long-term behaviour change [47]. Intervention development took an iterative approach with continuous feedback from stakeholders and PPI to enhance acceptability and implementation of the proposed service level change to clinical care delivery [48]. The complex intervention addresses professional (i.e. HCPs and CESs) and patient behaviour change and will be the first of its kind to be delivered as part of routine care to bridge the gap between research, policy and practice and maximise future scale-up. Moreover, STAMINA benefits from the addition of a novel 'safety to exercise' check for all participants to maximise safety and a unique approach to patient recruitment. Men with prostate cancer will be identified in routine NHS clinics but recruited by non-NHS researchers to reduce burden on NHS staff during unprecedented times (i.e. the COVID-19 pandemic).

The trial has the potential to significantly impact a large cohort of men with prostate cancer on ADT due to its rigorous approach to intervention development and the trial design, e.g. the broad eligibility criteria, long-term follow-up and because the prevalence and survival time for prostate cancer is continuing to rise [1]. Eight out of ten men now survive the disease for 10 years or more [1] and thus the patient and professional demand for such intervention continues to grow. The process evaluation will be essential for understanding if, why and how the intervention works in the real-world context and will contribute towards recommendations for future clinical care [49]. Moreover, if found to be effective, STAMINA could produce a blueprint for the integration of supervised exercise and behavioural support into other cancer and/or clinical services.

To date, the SLI has demonstrated good feasibility, fidelity, safety and acceptability when embedded into routine NHS care as part of a feasibility study [25]. This definitive pragmatic trial will evaluate the long-term clinical and cost-effectiveness of SLI when delivered in the real world and when compared to OUC.

Trial status

Protocol v10.0 05/07/2023. Recruitment began in January 2022 and will be ongoing until all interviews for the process evaluation are complete (~ January 2024) .

Abbreviations

ADT	Androgen deprivation therapy
CES	Clinical Exercise Specialist
CI	Confidence interval
CNST	Clinical Negligence Scheme for Trusts
COM-B	Capability, Opportunity, Motivation-Behaviour
CRUK	Cancer Research UK
CTRU	Leeds Clinical Trials Research Unit
EQ-5D-5L	EuroQol 5 Domains 5 Levels
DCE	Discrete choice experiment
FACIT-F	Functional Assessment of Chronic Illness Therapy – Fatigue
FACT-P	Functional Assessment of Cancer Therapy – Prostate
FCR	Fear of Cancer Recurrence (questionnaire)
GP	General practitioner
HCP	Healthcare professional
HEAP	Health Economic Analysis Plan
HRA	Health Research Authority
ICC	Intracluster correlation coefficient
ICER	Incremental cost-effectiveness ratio
ITT	Intention-to-treat
NIHR	National Institute for Health Research
NH	Nuffield Health
NHS	National Health Service
NICE	National Institute of Health and Care Excellence
NMB	Net monetary benefit
OUC	Optimised usual care
MAR	Missing at random
MRC	Medical Research Council
PCUK	Prostate Cancer UK
PMG	Programme management group
PPI	Patient and Public Involvement
PSC	Programme Steering Committee
QALY	Quality-adjusted life year
QoL	Quality of life
RCT	Randomised controlled trial
REC	Research Ethics Committee
RUSAE	Related and unexpected serious adverse event
SHU	Sheffield Hallam University
SLI	STAMINA lifestyle intervention
TMG	Trial management group

Supplementary Information

The online version contains supplementary material available at https://doi.org/10.1186/s13063-024-07989-y.

Additional file 1. This file contains three tables to describe each component of the intervention using the TIDieR framework: 1) STAMINA Lifestyle Intervention., 2) Healthcare Professional Intervention. and 3) Exercise Professional Intervention.

Acknowledgements

We are especially grateful to Nuffield Health for their tremendous support in the setting up and delivery of this trial. We would in particular like to thank Aidan Innes and Ben Kelly for their support, and all local Clinical Exercise Specialists and fitness managers. Nuffield Health sites include:

- Nuffield Health Plymouth Fitness & Wellbeing Gym
- Nuffield Health The Devonshire Health & Racquets Club
- Nuffield Health Nuneaton Fitness & Wellbeing Gym
- Nuffield Health Rugby Fitness & Wellbeing Gym
- Nuffield Health Warwick Fitness & Wellbeing Gym
- Nuffield Health Chesterfield Fitness & Wellbeing Gym
- Nuffield Health Nottingham Fitness & Wellbeing Gym
- Nuffield Health Sheffield Fitness & Wellbeing Gym
- Nuffield Health Crawley Fitness & Wellbeing Gym
- Nuffield Health Crawley Central Fitness & Wellbeing Gym
- Nuffield Health Liverpool Fitness & Wellbeing Gym
- Nuffield Health Derby Fitness & Wellbeing Gym
- Nuffield Health Cambridge Fitness & Wellbeing Gym
- Nuffield Health Yeovil Fitness & Wellbeing Gym
- Nuffield Health Didsbury Fitness & Wellbeing Gym
- Nuffield Health Cottingley Fitness & Wellbeing Gym
- Nuffield Health Gloucester Fitness & Wellbeing Gym
- Nuffield Health Hull Fitness & Wellbeing Gym

We are very grateful for the substantial contributions made by many to the setting up of this trial: our Sponsor team at Sheffield Teaching Hospitals NHS Foundation Trust; our PSC chaired by Peter Sasieni, and other members Alison Birtle, Richard Bryant and Rachel Elliott; our PMG and STAMINA co-applicants (Patrick Doherty, Liz Steed, Eileen Sutton, Diana Greenfield*, Dylan Morrissey, Suzanne Hartley and Malcolm Mason); PPIE lay member of our PSC (Geoff

Ogden), and PMG/TMG (Tom Baker) who reports on behalf of the PPI Group, co-chaired with John Kidder and Chris Allen; colleagues at the University of Leeds CTRU and Sheffield Hallam University who supported development and implementation of the trial protocol.
*Diana Greenfield is a National Institute for Health Research (NIHR) Senior Nurse Research Leader.
We would also like to offer our gratitude to the sites participating in the trial for their huge support in its delivery. Site personnel include principal investigators, research staff, clinical staff and Clinical Research Networks. Our trial sites are:
• University Hospitals Plymouth NHS Trust
• University Hospitals Coventry & Warwickshire NHS Trust
• Chesterfield Royal Hospital NHS Foundation Trust
• Nottingham University Hospitals NHS Trust
• Sheffield Teaching Hospitals NHS Trust
• Surrey and Sussex Healthcare NHS Trust
• Liverpool University Hospitals NHS Foundation Trust
• University Hospitals of Derby and Burton NHS Foundation Trust
• Cambridge University Hospital NHS Foundation Trust
• Somerset NHS Foundation Trust
• Stockport NHS Foundation Trust
• Bradford Teaching Hospitals NHS Foundation Trust
• Gloucestershire Hospitals NHS Foundation Trust
• Hull University Teaching Hospitals NHS Trust
• Clatterbridge Cancer Centre NHS Foundation Trust
• University Hospitals Bristol and Weston NHS Foundation Trust
Authors' contributions {31b}
All authors meet the International Committee of Medical Journal Editors (ICMJE) criteria for authorship and contributed to the work. DR is the chief investigator and conceived the trial. DR, LB, JB, AJF, ST, SR, SI, RT, AI, DM, JH and MC contributed to the trial design and to the development of the protocol. ST and AJF lead the process evaluation. DM leads the health economic evaluation. AJF, MC and EM developed the statistical analysis plan. SR, SI, AS, EMc, FD and CT perform trial and data management. EMc and SR produced a first draft of the manuscript. All authors read and approved the final manuscript.

Funding {4}
This trial is funded by the NIHR Programme Grants for Applied Research (PGfAR) RP-PG-1016-20007. The views expressed are those of the author(s) and not necessarily those of the NHS, the NIHR or the Department of Health and Social Care.

Availability of data and materials {29}
The Sponsor (Sheffield Teaching Hospitals NHS Foundation Trust), Coordinating Centre (Sheffield Hallam University) and the Clinical Trials Unit (CTRU, University of Leeds) will act as Data Controllers. Requests to access anonymised data should be made to CTRU-DataAccess@leeds.ac.uk in the first instance.

Declarations

Ethics approval and consent to participate {24}
West of Scotland Research Ethics Committee 1 20/WS/0069. Written, informed consent to participate will be obtained from all participants.

Consent for publication {32}
Not applicable.

Competing interests {28}
The authors declare one competing interest. Professor Liam Bourke is funded by the NIHR and acts as a scientific consultant for Boston Scientific Corp.

Author details
[1]Clinical Trials Research Unit, Leeds Institute of Clinical Trials Research, University of Leeds, Leeds LS2 9JT, UK. [2]Department of Allied Health Professions, Sheffield Hallam University, Sheffield S10 2BP, UK. [3]Division of Clinical Medicine, University of Sheffield, Sheffield S10 2RX, UK. [4]Division of Health Services Research, Leeds Institute of Health Sciences, University of Leeds, Leeds LS2 9JT, UK. [5]Nuffield Health, 2 Ashley Avenue, Epsom, Surrey KT18 5AL, UK. [6]Academic Unit of Health Economics, Leeds Institute of Health Sciences, University of Leeds, Leeds LS2 9JT, UK. [7]Wolfson Institute of Population Health, Queen Mary University of London, Yvonne Carter Building, 58 Turner Street, London E1 2AB, UK. [8]Division of Psychology and Mental Health in the School of Health Sciences, The University of Manchester, Manchester M13 9PL, UK. [9]Department of Urology, Sheffield Teaching Hospitals NHS Foundation Trust, Royal Hallamshire Hospital, Glossop Road, Sheffield S10 2JF, UK.

Received: 1 December 2023 Accepted: 5 February 2024
Published online: 12 April 2024

References

1. Cancer Research UK. Prostate Cancer Statistics. https://www.cancerresearchuk.org/health-professional/cancer-statistics/statistics-by-cancer-type/prostate-cancer. Accessed 05 May 2023.
2. Edmunds K, Tuffaha H, Galvao DA, Scuffham P, Newton RU. Incidence of the adverse effects of androgen deprivation therapy for prostate cancer: a systematic literature review. Support Care Cancer. 2020;28(5):2079–93.
3. Mason MD, Parulekar WR, Sydes MR, Brundage M, Kirkbride P, Gospodarowicz M, et al. Final report of the intergroup randomized study of combined androgen-deprivation therapy plus radiotherapy versus androgen-deprivation therapy alone in locally advanced prostate cancer. J Clin Oncol. 2015;33(19):2143–50.
4. James ND, Sydes MR, Clarke NW, Mason MD, Dearnaley DP, Spears MR, et al. Addition of docetaxel, zoledronic acid, or both to first-line long-term hormone therapy in prostate cancer (STAMPEDE): survival results from an adaptive, multiarm, multistage, platform randomised controlled trial. Lancet. 2016;387(10024):1163–77.
5. Schroder FH, Kurth KH, Fossa SD, Hoekstra W, Karthaus PP, De Prijck L, et al. Early versus delayed endocrine treatment of T2–T3 pN1-3 M0 prostate cancer without local treatment of the primary tumour: final results of European Organisation for the Research and Treatment of Cancer protocol 30846 after 13 years of follow-up (a randomised controlled trial). Eur Urol. 2009;55(1):14–22.
6. Konijeti R, Kibel AS. Androgen deprivation therapy for localized and nonmetastatic prostate cancer: too much of a good thing? Eur Urol. 2012;61(6):1129–30 discussion 31.
7. Nguyen PL, Alibhai SM, Basaria S, D'Amico AV, Kantoff PW, Keating NL, et al. Adverse effects of androgen deprivation therapy and strategies to mitigate them. Eur Urol. 2015;67(5):825–36.
8. Wilding S, Downing A, Wright P, Selby P, Watson E, Wagland R, et al. Cancer-related symptoms, mental well-being, and psychological distress in men diagnosed with prostate cancer treated with androgen deprivation therapy. Qual Life Res. 2019;28(10):2741–51.
9. Smith MR, Lee H, Nathan DM. Insulin sensitivity during combined androgen blockade for prostate cancer. J Clin Endocrinol Metab. 2006;91(4):1305–8.
10. Braga-Basaria M, Dobs AS, Muller DC, Carducci MA, John M, Egan J, et al. Metabolic syndrome in men with prostate cancer undergoing long-term androgen-deprivation therapy. J Clin Oncol. 2006;24(24):3979–83.
11. Keating NL, O'Malley AJ, Freedland SJ, Smith MR. Diabetes and cardiovascular disease during androgen deprivation therapy: observational study of veterans with prostate cancer. J Natl Cancer Inst. 2010;102(1):39–46.
12. Keating NL, O'Malley AJ, Freedland SJ, Smith MR. Does comorbidity influence the risk of myocardial infarction or diabetes during androgen-deprivation therapy for prostate cancer? Eur Urol. 2013;64(1):159–66.
13. Van Hemelrijck M, Garmo H, Holmberg L, Ingelsson E, Bratt O, Bill-Axelson A, et al. Absolute and relative risk of cardiovascular disease in men with prostate cancer: results from the population-based PCBaSe Sweden. J Clin Oncol. 2010;28(21):3448–56.
14. Cheung AS, de Rooy C, Hoermann R, Lim Joon D, Zajac JD, Grossmann M. Quality of life decrements in men with prostate cancer undergoing androgen deprivation therapy. Clin Endocrinol (Oxf). 2017;86(3):388–94.
15. Bourke L, Chico TJ, Albertsen PC, Hamdy FC, Rosario DJ. Cardiovascular risk in androgen suppression: underappreciated, under-researched and unresolved. Heart. 2012;98(5):345–8.
16. Bourke L, Gilbert S, Hooper R, Steed LA, Joshi M, Catto JW, et al. Lifestyle changes for improving disease-specific quality of life in sedentary men on long-term androgen-deprivation therapy for advanced prostate cancer: a randomised controlled trial. Eur Urol. 2014;65(5):865–72.

17. Gunlusoy B, Ceylan Y, Koskderelioglu A, Gedizlioglu M, Degirmenci T, Ortan P, et al. Cognitive effects of androgen deprivation therapy in men with advanced prostate cancer. Urology. 2017;103:167–72.
18. DiBlasio CJ, Hammett J, Malcolm JB, Judge BA, Womack JH, Kincade MC, et al. Prevalence and predictive factors for the development of de novo psychiatric illness in patients receiving androgen deprivation therapy for prostate cancer. Can J Urol. 2008;15(5):4249–56 discussion 56.
19. National Institute for Health Care Excellence. Prostate cancer: diagnosis and management. https://www.nice.org.uk/guidance/ng131/chapter/recommendations#people-having-hormone-therapy. Accessed 31 May 2023.
20. European Association of Urology. EAU Guidelines on Prostate Cancer. https://uroweb.org/guidelines/prostate-cancer. Accessed 02 June 2023.
21. Bourke L, Turner R, Greasley R, Sutton E, Steed L, Smith D, et al. A multi-centre investigation of delivering national guidelines on exercise training for men with advanced prostate cancer undergoing androgen deprivation therapy in the UK NHS. Plos One. 2018;13(7):e0197606.
22. Bourke L, Smith D, Steed L, Hooper R, Carter A, Catto J, et al. Exercise for men with prostate cancer: a systematic review and meta-analysis. Eur Urol. 2016;69(4):693–703.
23. Michie S, Atkins L, West R. The Behaviour Change Wheel: A Guide to Designing Interventions. 1st ed. Great Britain: Silverback Publishing; 2014.
24. Turner RR, Arden MA, Reale S, Sutton E, Taylor SJC, Bourke L, et al. The development of a theory and evidence-based intervention to aid implementation of exercise into the prostate cancer care pathway with a focus on healthcare professional behaviour, the STAMINA trial. BMC Health Serv Res. 2021;21(1):273.
25. Reale S, Turner RR, Sutton E, Steed L, Taylor SJC, Morrissey D, et al. Embedding supervised exercise training for men on androgen deprivation therapy into standard prostate cancer care: a feasibility and acceptability study (the STAMINA trial). Sci Rep. 2021;11(1):12470.
26. Esper P, Mo F, Chodak G, Sinner M, Cella D, Pienta KJ. Measuring quality of life in men with prostate cancer using the functional assessment of cancer therapy-prostate instrument. Urology. 1997;50(6):920–8.
27. Yellen SB, Cella DF, Webster K, Blendowski C, Kaplan E. Measuring fatigue and other anemia-related symptoms with the Functional Assessment of Cancer Therapy (FACT) measurement system. J Pain Symptom Manage. 1997;13(2):63–74.
28. Godin G. The Godin-Shephard leisure-time physical activity questionnaire. Health Fitness J Can. 2011;4(1):18–22.
29. Humphris GM, Watson E, Sharpe M, Ozakinci G. Unidimensional scales for fears of cancer recurrence and their psychometric properties: the FCR4 and FCR7. Health Qual Life Outcomes. 2018;16(1):30.
30. Keyworth C, Epton T, Goldthorpe J, Calam R, Armitage CJ. Acceptability, reliability, and validity of a brief measure of capabilities, opportunities, and motivations ("COM-B"). Br J Health Psychol. 2020;25(3):474–501.
31. Herdman M, Gudex C, Lloyd A, Janssen M, Kind P, Parkin D, et al. Development and preliminary testing of the new five-level version of EQ-5D (EQ-5D-5L). Qual Life Res. 2011;20(10):1727–36.
32. de Bekker-Grob EW, Ryan M, Gerard K. Discrete choice experiments in health economics: a review of the literature. Health Econ. 2012;21(2):145–72.
33. Association of Chartered Physiotherpaists in Cardiovascular Rehabilitation. Healthcare Professionals publications. https://www.acpicr.com/publications/healthcare-professionals/. Accessed April 01 2023.
34. Bourke L, Stevenson R, Turner R, Hooper R, Sasieni P, Greasley R, et al. Exercise training as a novel primary treatment for localised prostate cancer: a multi-site randomised controlled phase II study. Sci Rep. 2018;8(1):8374.
35. Prostate Cancer UK. All Publications. https://shop.prostatecanceruk.org/all-publications. Accessed 05 May 2023.
36. Support Macmillan Cancer. Booklets. https://www.macmillan.org.uk/cancer-information-and-support/stories-and-media/booklets. Accessed 05 May 2023.
37. Cancer Research UK. Publications. https://publications.cancerresearchuk.org/. Accessed 05 May 2023.
38. Reale S, Turner RR, Sutton E, Taylor SJC, Bourke L, Morrissey D, et al. Towards implementing exercise into the prostate cancer care pathway: development of a theory and evidence-based intervention to train community-based exercise professionals to support change in patient exercise behaviour (The STAMINA trial). BMC Health Serv Res. 2021;21(1):264.
39. Iliffe S, Kendrick D, Morris R, Masud T, Gage H, Skelton D, et al. Multicentre cluster randomised trial comparing a community group exercise programme and home-based exercise with usual care for people aged 65 years and over in primary care. Health Technol Assess. 2014;18(49):1–105 vii-xxvii.
40. de LafayeMicheaux P, Liquet B, Marque S, Riou J. Power and sample size determination in clinical trials with multiple primary continuous correlated endpoints. J Biopharm Stat. 2014;24(2):378–97.
41. Data Protection Act, 2018. http://www.legislation.gov.uk/ukpga/2018/12/contents/enacted. Accessed 01 June 2023.
42. Flight L, Allison A, Dimairo M, Lee E, Mandefield L, Walters SJ. Recommendations for the analysis of individually randomised controlled trials with clustering in one arm - a case of continuous outcomes. BMC Med Res Methodol. 2016;16(1):165.
43. Drummond MF, Sculpher MJ, Claxton K, Stoddart GL, Torrance GW. Methods for the Economic Evaluation of Health Care Programmes. 4th ed. Oxford: Oxford University Press; 2015.
44. Barton GR, Briggs AH, Fenwick EA. Optimal cost-effectiveness decisions: the role of the cost-effectiveness acceptability curve (CEAC), the cost-effectiveness acceptability frontier (CEAF), and the expected value of perfection information (EVPI). Value Health. 2008;11(5):886–97.
45. Moore GF, Audrey S, Barker M, Bond L, Bonell C, Hardeman W, et al. Process evaluation of complex interventions: medical research council guidance. BMJ. 2015;350:h1258.
46. Steckler A, Linnan L, editors. Process Evaluation for Public Health Interventions and Research. 1st ed. San Francisco: Jossey-Bass; 2002.
47. O'Cathain A, Croot L, Duncan E, Rousseau N, Sworn K, Turner KM, et al. Guidance on how to develop complex interventions to improve health and healthcare. BMJ Open. 2019;9(8):e029954.
48. Yardley L, Morrison L, Bradbury K, Muller I. The person-based approach to intervention development: application to digital health-related behavior change interventions. J Med Internet Res. 2015;17(1):e30.
49. Limbani F, Goudge J, Joshi R, Maar MA, Miranda JJ, Oldenburg B, et al. Process evaluation in the field: global learnings from seven implementation research hypertension projects in low-and middle-income countries. BMC Public Health. 2019;19(1):953.

Publisher's Note

Springer Nature remains neutral with regard to jurisdictional claims in published maps and institutional affiliations.

CLINICAL TRIALS RESEARCH UNIT (CTRU)

UNIVERSITY OF LEEDS

FINAL

STATISTICAL ANALYSIS PLAN

SUPPORTED EXERCISE TRAINING FOR MEN WITH PROSTATE CANCER ON ANDROGEN DEPRIVATION THERAPY - THE STAMINA PROGRAMME

A RANDOMISED CONTROLLED TRIAL OF THE CLINICAL AND COST EFFECTIVENESS OF THE STAMINA LIFESTYLE INTERVENTION (SLI) COMPARED WITH OPTIMISED USUAL CARE (OUC)

VERSION 3.0

FEBRUARY 2025

Trial Statistician:	Jamie Stokes
Supervising Statistician:	Michelle Collinson
CTRU Scientific Lead:	Professor Amanda Farrin
Senior Trial Manager:	Emma McNaught
Senior Data Manager:	Christopher Taylor
CTRU Project Delivery Lead:	Florence Day
Chief Investigator:	Professor Derek Rosario

Copyright © 2007 CTRU, University of Leeds. All rights reserved.

Contents

1. TRIAL SUMMARY ... 3
2. ABBREVIATIONS ... 5
3. INTRODUCTION ... 6
3.1. Background ... 6
3.2. Design ... 7
3.3. Aims ... 7
3.3.1. Primary objective ... 8
3.3.2. Secondary objectives ... 8
3.3.3. Process Evaluation objectives ... 8
3.4. Randomisation ... 9
3.5. Sample size and expected accrual ... 9
3.6. Planned analyses ... 9
4. ENDPOINTS ... 10
4.1. Primary endpoints ... 10
4.1.1. Primary endpoint estimand ... 10
4.2. Secondary endpoints ... 10
4.2.1. Process evaluation endpoint ... 11
4.3. Derivation of endpoints ... 11
4.4. Missing data ... 16
4.4.1. Investigation of missing data pattern ... 16
4.4.2. Method of imputation ... 17
5. POPULATIONS ... 17
5.1. Screening population ... 17
5.2. Eligibility ... 17
5.3. Intention to treat population ... 18
5.4. CACE population ... 18
6. DATA HANDLING ... 18
6.1. Data monitoring ... 18
6.2. Data validation ... 19
7. DATA ANALYSIS ... 19
7.1. General calculations ... 20
7.2. Trial summary ... 20
7.2.1. Milestones ... 20
7.2.2. Populations ... 20
7.2.3. Participant summary ... 20
7.3. Characteristics ... 21
7.3.1. Patient screening characteristics ... 21
7.3.2. Participant baseline characteristics and clinical measurements ... 21
7.3.3. Baseline outcomes ... 22
7.4. Intervention delivery ... 22
7.4.1. Participant level summaries ... 22
7.4.2. Supervised sessions ... 23
7.4.3. Unsupervised sessions ... 24
7.4.4. Clinical Exercise Specialist (cluster) level summaries ... 25
7.5. Withdrawals (F15, F16) ... 25
7.6. Deaths (F14) ... 25
7.7. Unblinding (F21) ... 25
7.8. Protocol violations and deviations (F17) ... 26
7.9. Primary endpoint analysis ... 26
7.9.1. Sensitivity Analysis ... 26
7.9.2. CACE Analysis ... 26
7.10. Secondary endpoint analysis ... 27

Copyright © 2007 CTRU, University of Leeds. All rights reserved.

7.11. **ADT symptom index (F57)** 28
7.12. **Physical (F8, F9, F10)** 28
7.13. **Safety (F18)** 28
7.14. **Exploratory analyses** 28
7.14.1. Moderator analysis 28
7.14.2. Mediator analysis 29
7.14.3. Dose-response analysis 30
8. REPORTING AND DISSEMINATION OF RESULTS 30
9. REFERENCES 30
10. APPENDICES 32
10.1. **Appendix 1 – Key data items** 32
10.2. **Appendix 2 - Internal pilot and progression criteria** 33
10.2.1. Objectives 33
10.2.2. Analysis 34
10.2.3. Results 34
10.2.4. Recruitment 35
10.2.5. Follow-up at 3 months 37
10.2.6. 12 weeks (participant) intervention adherence 41
11. APPROVAL OF ANALYSIS PLAN 50

1. Trial Summary

**Trial Title**	**S**upported exercise **T**r**A**ining for **M**en w**I**th prostate ca**N**cer on **A**ndrogen deprivation therapy (**STAMINA)**: a randomised controlled trial of the clinical and cost effectiveness of the STAMINA lifestyle intervention compared with optimised usual care, including internal pilot and parallel process evaluation.	
**Trial short title**	STAMINA Trial	
**Trial Design**	Multi-centre, two arm, Randomised Controlled Trial (RCT) with internal pilot and embedded cost-effectiveness and process evaluation	
**Key inclusion criteria**	Men with PCa on androgen deprivation therapy (ADT) or likely to start ADT within the following 3 months	
**Planned Sample Size**	Target of 697 participants	
**Experimental Intervention duration**	12 months	
**Follow up schedule**	At 3, 6, 12 months (Primary endpoint) post-randomisation for all participants	
**Planned Trial Period**	September 2021 to end-Jan 2025	
**Actual Trial Period**	January 2022 to end-Mar 2025	
	**Objectives**	**Outcome Measures**
**Primary (12 months post randomisation)**	Prostate cancer-specific quality of life.<br>Cancer specific fatigue.	FACT-P total score<br>FACT-F FS subscale
**Secondary (3, 6, 12 months post randomisation)**	Prostate cancer-specific quality of life.	FACT-P total score
	Physical, Social/Family, Emotional, and Functional wellbeing.	FACT-P subscales

Copyright © 2007 CTRU, University of Leeds. All rights reserved.

	Cancer specific fatigue.	FACT-F FS subscale
	Cancer-specific quality of life	FACT-G total score
	Health utility	EQ-5D-5L
	Healthcare Utilisation	Resource Use Diary
	Leisure time physical activity	Godin questionnaire
	Fear of cancer recurrence	FCR4 and FCR7
	Impact of side effects of ADT	ADT Symptom Index
	Capability, opportunity and motivation for behaviour change	COM-B Questionnaire
	Functional capacity and body composition	Blood pressure, chair sit-to-stand, waist and hip circumference, weight.
	Adverse Events (RUSAEs)	Safety CRF
	Cost-effectiveness	Cost per Quality-Adjusted Life Year (QALY)
**Active Intervention**	The STAMINA lifestyle intervention (SLI) is an embedded, behaviourally informed, lifestyle intervention within cancer care to provide NICE guidance exercise supervision delivery for men with PCa on ADT integrated into routine clinical follow-up. We will examine whether the intervention improves quality of life and fatigue in men being treated for prostate cancer with ADT.	
**Optimised Usual Care**	Usual prostate cancer follow-up, optimised to include 'safe to exercise' checks and provision of local and national cancer survivorship support information.	
**Internal trial pilot**	To assess recruitment, follow-up rates and intervention adherence rates after 12 months of recruitment; we will proceed with the trial unchanged if we are recruiting an average of at least 1 participant per site per month, at least 75% of participants are completing their 3 month outcomes and at least 75% of participants are completing their supervised exercise sessions. We will also monitor for evidence of potential selection bias.	
**Process evaluation (PE)**	A parallel PE using quantitative data gathered as part of the trial as well as relevant associated qualitative data with the aim of informing the interpretation of the trial results will be carried out. Should the trial reveal a positive finding, then the PE will inform the optimal implementation into the NHS.<br><br>Quantitative data collected during the trial will include numbers recruited to the intervention, numbers both taking up and completing the intervention and the number of sessions delivered and received.	

Copyright © 2007 CTRU, University of Leeds. All rights reserved.

Amendments

- The primary endpoint estimands have been described.
- Details of the strategies for handling missing data have been added.
- Planned summaries of intervention delivery have been described.
- Planned summaries of withdrawals, deaths, unblindings, protocol violations/deviations and safety have been added.
- Details of the primary and secondary endpoint analyses have been added.
- The CACE population and analysis have been described.
- Mediator and moderators together with relevant analysis approaches have been defined.

Revision History

Protocol version no.	Updated SAP version no.	Section revised	Description of and reason for change	Date changed
11.0	2.0	4, 5, 6, 7	Addition of items listed in Amendments.	18/02/2025
11.0	3.0	4.1.1, 4.4	Clarification of intercurrent event and missing data strategies	28/02/2025

2. Abbreviations

ADT	Androgen deprivation therapy
AE	Adverse event
ARI	Androgen Receptor Inhibitor
BMI	Body Mass Index
CES	Clinical Exercise Specialist
CI	Chief Investigator
CRUK	Cancer Research UK
CTRU	Clinical Trials Research Unit
FACT	Functional Assessment of Cancer Therapy
HEAP	Health Economic Analysis Plan
ICER	Incremental Cost Effectiveness Ratio
ITT	Intention to Treat
NH	Nuffield Health
NHS	National Health Service
NICE	National Institute for Health and Care Excellence
OUC	Optimised Usual Care

Copyright © 2007 CTRU, University of Leeds. All rights reserved.

PCa	Prostate cancer
PEAP	Process Evaluation Analysis Plan
QALY	Quality Adjusted Life Year
QoL	Quality of life
SAP	Statistical Analysis Plan
SLI	STAMINA Lifestyle Intervention
SOP	Standard Operating Procedure

3. Introduction

This is the Statistical Analysis Plan (SAP) for the final analysis of the STAMINA trial which has been developed using protocol version 11.0 (1st February 2024).

STAMINA is a randomised, controlled, multi-centre trial (with an internal pilot) designed to determine the clinical and cost-effectiveness of an exercised-based lifestyle intervention programme in improving cancer-specific quality of life and reducing fatigue for men with prostate cancer, receiving Androgen deprivation therapy (ADT).

3.1. Background

Prostate cancer (PCa) is common and responsible for a quarter of all new diagnoses of male cancers in the UK (CRUK) (1) with around 50% being locally advanced or metastatic at presentation. ADT remains the cornerstone of therapy for more advanced disease. It is usually combined with radiotherapy for locally advanced disease (2), with Taxane-based chemotherapy for metastatic disease (3), as sole treatment or in conjunction with more novel androgenic signalling inhibitors.

ADT is associated with significant adverse events (AEs). The impact of these AEs on QoL is increasingly recognised (4-6) as well as their impact on overall healthcare costs/treatment costs for PCa (7). Of the numerous strategies described to manage ADT-AEs, exercise-training is the only evidence-based intervention with beneficial effects on fatigue and disease-specific QoL exceeding clinically relevant thresholds (8), improvement in cardiovascular health (8) and potential benefits for mental health (9, 10).

In 2014 the National Institute of Health and Care Excellence guidelines on PCa recommended 12 weeks of combined resistance and aerobic exercise as standard treatment (NICE CG175) for men on ADT, based on findings from non-NHS practice (8, 11-13). The 2017 European Association of Urology guidelines made similar recommendations (14). Nevertheless, meagre provision of exercise services exists for men on ADT.

The majority of men requiring ADT are sedentary and overweight; indeed, these are risk factors for advanced PCa and PCa mortality (15). Despite established short-term benefits of exercise, maintaining such men's exercise behaviour is challenging (16). A diagnosis of PCa can result in improved exercise behaviour in some men: this could be exploited in many more. Advice from clinical teams has a significant influence on behaviour, so-called 'teachable moments' to improve healthy behaviour (17, 18).

There is a consistent message of unmet needs and ongoing support requirements from men undergoing treatment for PCa (19, 20). As men on ADT remain on life-long follow-up for disease-monitoring and assessment of treatment AEs, this provides an opportunity to involve the clinical team in initiating and supporting behaviour

Copyright © 2007 CTRU, University of Leeds. All rights reserved.

change. Clearly, should exercise advice and support from the healthcare team alone under these circumstances be sufficient in promoting and maintaining improved exercise behaviour, this might well prove to be sufficient to deliver clinically meaningful improvement in prostate-cancer specific quality of life and ADT-induced fatigue.

Consistent quality-assured exercise provision is essential. Although some Charity programmes for delivery exist, referral and attendance are inconsistent and quality assurance is lacking. Findings from our previous work showed considerable variability in usual care with regards to exercise delivery at the vast majority of NHS sites, with most not delivering the NICE-specific exercise recommendations for men on ADT. Nuffield Health (NH) is a non-profit organisation with 130 gym facilities nationally and a track-record in delivering exercise interventions to clinical populations (21). NH has invested heavily in staff training to deliver exercise to the general public and to various clinical populations and provide a robust partner for testing the delivery of a behaviourally informed exercise intervention.

3.2. Design

The STAMINA trial is a definitive, multi-centre, two arm, individually randomised controlled superiority trial with a two-level partially nested design, an internal pilot with clear progression criteria, a cost-effectiveness analysis and parallel process evaluation.

697 eligible, consenting men will be randomly allocated (5:4) to either the STAMINA lifestyle intervention (SLI, 384 men), or Optimised Usual Care (OUC, 313 men). Clinical teams will deliver usual care to all men recruited to the trial, regardless of their randomised allocation. A larger number of men were randomised to the SLI arm to account for a clustering effect at the level of the Clinical Exercise Specialist.

The STAMINA lifestyle intervention is behaviourally informed to support embedding of NICE guidance for men with PCa on ADT into routine clinical follow-up. SLI is focussed primarily on supervised exercise training combining resistance and aerobic exercise with application of the behavioural skills learnt to other patient negotiated goals, including dietary modification. Each participant allocated to the SLI arm of the trial will receive supervised exercise sessions (one-to-one and group delivery), behavioural support and programme reviews. The sessions will involve both aerobic and resistance components on a twice weekly basis during the initial twelve weeks of the exercise programme, tailored to individual participant requirements. A programme review into each participant’s progress will be carried out at twelve weeks, six months and twelve months after beginning SLI. After the initial twelve weeks, exercise sessions may be supervised remotely or face-to-face for the remaining nine months of the programme. During the entire programme the Clinical Exercise Specialist (CES) will have a catch-up call or visit every six weeks with each participant to discuss progress.

Outcomes will be collected at three, six- and twelve-months post-randomisation. This trial has two multiple primary endpoints; the SLI will be deemed clinically different from OUC if a significant difference is observed in either cancer-specific quality of life (FACT-P) or fatigue (FACT-F FS subscale) measured at twelve months post-randomisation. A statistically significant difference in either primary endpoint is deemed sufficient to declare effectiveness of the SLI as both outcomes (cancer-specific quality of life and fatigue) are clinically important in their own right and are of comparable importance to both patients and clinicians.

Participant recruitment, three-month follow-up and twelve-week intervention adherence was assessed in the internal pilot twelve months after the start of recruitment, based on a traffic light system of green (go), amber (review) and red (stop). Details of the progression criteria and analysis of the internal pilot can be found in Appendix 1.

3.3. Aims

The aim of the STAMINA trial is to establish whether the SLI is clinically and cost effective in improving cancer-specific quality of life or reducing fatigue as a result of ADT in men with prostate cancer, when compared with OUC.

Copyright © 2007 CTRU, University of Leeds. All rights reserved.

The aim of the parallel process evaluation is to inform the interpretation of the trial's results (regardless of evidence of intervention effect) and, if the intervention is shown to benefit patients, to inform the implementation of the intervention in the NHS.

3.3.1. Primary objective

To establish whether SLI improves cancer-specific quality of life, measured using the Functional Assessment of Cancer Therapy – Prostate (FACT-P) and fatigue caused by ADT measured using the Functional Assessment of Cancer Therapy – Fatigue (FACT-F FS subscale) at twelve months.

3.3.2. Secondary objectives

1. To establish whether the intervention reduces prostate cancer specific quality of life at three and six-months post-randomisation using FACT-P total score.
2. To establish whether the intervention promotes physical, social, emotional, and functional wellbeing at three, six- and twelve-months post-randomisation using FACT-P subdomains.
3. To establish whether the intervention reduces cancer specific fatigue at three and six- months post-randomisation using FACT-F FS subscale.
4. To establish whether the intervention improves cancer-specific quality of life at three, six- and twelve-months post-randomisation using the FACT-G total score.
5. To establish whether the intervention improves leisure time physical activity, measured using the Godin questionnaire at three, six, and twelve months.
6. To establish whether the intervention reduces fear of cancer recurrence measured using FCR4 and FCR7 at three, six and twelve months.
7. To establish whether the intervention improves functional capacity, body composition, measured using blood pressure, chair sit-to-stand, waist and hip circumference and weight at three, six, and twelve months.
8. To establish RUSAE rates, and severity, measured using a Safety Case Report Form (CRF).
9. To assess whether the SLI reduces adverse effects, commonly associated with ADT measured using a trial specific ADT symptom index at three, six and twelve months.
10. To assess participants' perceptions of capabilities, opportunities, and motivations to perform a target behaviour using the COM-B Questionnaire
11. To establish whether the intervention is cost-effective, assessed using incremental cost effectiveness ratios (ICERs). The outcome of interest is cost per incremental quality-adjusted life year (QALY) at twelve months with QALYs being derived from the EQ-5D-5L. A decision model will allow estimation of cost-effectiveness over a lifetime horizon.
12. To determine patient preferences and to predict demand for alternative extended exercise programme features using a discrete choice experiment.
13. To assess mediator and moderator variables which influence engagement with and benefit from treatment
14. To quantitatively assess intervention implementation, uptake and fidelity to inform the Process Evaluation

3.3.3. Process Evaluation objectives

The aims of the process evaluation are as follows:

- To understand trial recruitment performance
- To describe intervention reach, dose delivered and dose received
- To describe the fidelity of intervention delivery
- To understand how the intervention was experienced and understood by patients
- To explore the organisational implications of embedding and sustaining the intervention in preparation for wider NHS roll-out (underpinned by consideration of Normalization Process Theory) (22)

This statistical analysis plan does not cover secondary objective 10 (Section 3.3.2) or the process evaluation objectives specified in Section 3.3.3 (with the exception of dose delivered and received) as these will be covered

Copyright © 2007 CTRU, University of Leeds. All rights reserved.

by a separate Process Evaluation Analysis Plan (PEAP) developed by the PE team. Secondary objectives 11-12 will be covered by a separate Health Economics Analysis Plan (HEAP) developed by the HE team.

3.4. Randomisation

Registered participants were randomised following confirmation of eligibility and completion of at least the baseline FACT-P and FACT-F FS subscale questionnaires. Randomisation was performed using the CTRU automated 24-hour randomisation system (GEN24) which was accessed via the web.

Participants were individually randomised on a 5:4 basis (STAMINA Lifestyle Intervention: Optimised Usual Care) to ensure the trial is powered for the primary objectives whilst accounting for the partially nested trial design. A computer-generated minimisation programme incorporating a random element was used to ensure arms are well-balanced for the following participant characteristics (stratification factors):

- Age: <70 years OR ≥70 years
- Duration on ADT: ≤ 12 weeks OR > 12 weeks
- Receiving chemotherapy and/or novel androgen receptor inhibitors (ARI): yes OR no
- Receiving radiotherapy: yes OR no

3.5. Sample size and expected accrual

This trial is powered to detect an effect in either FACT-P or FACT-F FS subscale, i.e. multiple primary endpoints. A total sample size of 697 men is required to provide 90% power to detect a small to moderate effect size of either 0.33 in FACT-P or 0.35 in FACT-F FS subscale at the 5% significance level and assuming 30% loss to follow-up (22). This equates to an 8-point difference in FACT-P and a 3-point difference in FACT-F FS subscale and assumes a maximum standard deviation of 24 (FACT-P) and 8.6 (FACT-F FS subscale). Clustering at the level of the CES in the intervention arm is accounted for using an ICC of 0.05 (23) and a coefficient of variation of 0.6 accounts for variation in the number of men each CES will deliver the intervention to. This results in a total sample size of 697 men; 313 in the OUC arm and 384 in the SLI arm. An average cluster size (i.e. number of men per CES) of 6 (range: 1 to 14) requires a total of 64 CES to deliver the intervention. If each gym has 3 CES available at any one time to deliver the intervention, 22 gyms are required; if each gym has 6 CES available, 11 gyms are required.

The sample size calculation takes into account the correlation between the two primary endpoints based on methodology by Micheaux et al., (2014) (24). A correlation between FACT-P and FACT-F FS subscale of 0.7 (95% CI: 0.58-0.78) was observed at baseline in the pilot data (25). The lower bound of the confidence interval for the correlation estimate (0.58) was incorporated into the calculations. After accounting for multiple testing with correlated continuous multiple primary endpoints, the adjusted alpha level is 0.02768.

The trial planned to recruit participants over 21 months between September 2021 and May 2023 however due to delays brought on by the COVID-19 pandemic relating to site capacity and necessary changes to the delivery of the trial, a 16-month extension was approved by the funder in March 2023 allowing recruitment to continue to August 2023.

3.6. Planned analyses

Progression to the main trial together with an examination of the sample size assumptions was assessed after 12 months of recruitment in the internal pilot. No further formal interim analysis of primary or secondary outcomes will be undertaken.

Analysis will take place in two stages. Stage 1 will cover analysis of all data collected up to and including baseline / randomisation. Stage 1 analysis will begin once all relevant data has been collected and cleaned (anticipated Summer 2024). Stage 2 will cover all remaining trial analyses, including analysis of the primary and secondary endpoints. Stage 2 analysis will begin once the final participant has reached twelve months post-randomisation

Copyright © 2007 CTRU, University of Leeds. All rights reserved.

and all data has been collected and cleaned (anticipated Autumn 2024). Data from participants recruited in the internal pilot will be incorporated into both the Stage 1 and Stage 2 analyses where relevant.

The statistical report of the final analysis will follow the guidance defined in G21 – Statistical Reports (version 4.0).

4. Endpoints

4.1. Primary endpoints

The multiple primary endpoints are measured by the Functional Assessment of Cancer Therapy – Prostate (FACT-P) and the fatigue subscale of the Functional Assessment of Cancer Therapy - Fatigue (FACT-F FS subscale) at twelve months post-randomisation. The null hypothesis for the primary endpoints of the trial is that no difference in the total score of the FACT-P or the FACT-F FS subscale exists between trial arms.

SLI will be deemed as clinically different to OUC if a statistically significant difference between trial arms is detected in either the FACT-P total score or the FACT-F FS subscale. Statistical significance will be determined by comparing outcomes using an adjusted alpha level of 0.02768.

4.1.1. Primary endpoint estimand

The elements to inform the estimands for the FACT-P total score and FACT-F FS subscale primary endpoints are defined as follows:

- Population: Men with prostate cancer on androgen deprivation therapy or likely to start ADT within the following 3 months as defined by the inclusion/exclusion criteria (Section 5.2).
- Treatment: The STAMINA Lifestyle Intervention, an embedded, behaviourally informed lifestyle intervention within cancer care to provide NICE guidance exercise supervision delivery for men with PCa on ADT integrated into routine clinical follow-up.
- Variable(s) of interest: The FACT-P total score and the FACT-F FS subscale.
- Summary measure: Difference in mean FACT-P total score and FACT-F FS subscale at 12 months, with 97.232% confidence interval.
- Handling of intercurrent events:
    - Deaths: The while on treatment strategy will explore the effect of the intervention until death. Missing FACT-P total score / FACT-F FS subscale scores due to death will not be imputed.
    - Withdrawal / protocol deviations or violations / lack of adherence: The treatment policy strategy will explore the effect of the intervention regardless of whether or not the intercurrent event occurred. FACT-P total score / FACT-F FS subscale scores collected after the intercurrent event will be used in the primary analysis. Missing FACT-P total score / FACT-F FS subscale scores due the intercurrent event will be imputed in the primary analysis where appropriate, as per Section 4.4.

The estimand for each primary endpoint can be stated as: “In men with PCa on ADT, or likely to start ADT within 3 months, what is the difference in mean FACT-P total score/FACT-F FS subscale score between SLI and optimised usual care, at 12 months from randomisation or death (whichever occurs first), regardless of withdrawal, deviations from the protocol or adherence to the intervention”.

The estimands for the participant reported secondary endpoints will follow the same definition as per the primary endpoints, replacing the variable of interest with the relevant secondary outcome.

4.2. Secondary endpoints

Secondary endpoints will be measured at three, six- and twelve-months post randomisation and are as follows:
<u>Participant reported outcome measures (PROMs)</u>

Copyright © 2007 CTRU, University of Leeds. All rights reserved.

- FACT-P total score at three and six months
- Physical, Social/Family, Emotional and Function wellbeing, assessed using FACT-P sub domains
- Cancer specific fatigue (FACT-F) FS subscale at three and six months
- Cancer-specific quality of life assessed using the FACT-G total score
- Leisure time physical activity assessed using the Godin questionnaire
- Fear of recurrence and psychological distress markers assessed using FCR4 and FCR7
- Adverse effects of ADT assessed using trial specific ADT Symptom Index*

Physical

- Functional capacity and body composition assessed by blood pressure, chair sit-to-stand, waist and hip circumference and weight.*

Safety

- Adverse events assessed by:
    - Number and proportion of RUSAEs*
    - Number of RUSAEs per participant*
    - Details of RUSAEs including severity*
    - Number and proportion of deaths*
    - Details of deaths including primary cause and timing*

Moderators (measured at baseline)

- A Body Shape Index (ABSI)
- IMD decile
- Cardiorespiratory comorbidities
- Musculoskeletal comorbidities
- Disease status
- Chair sit-to-stand
- Age
- Second line treatment
- Radiotherapy
- Length of time on ADT
- FACT-F FS subscale (as a moderator for FACT-P only)

Mediators

- Godin activity level
- A Body Shape Index (ABSI)
- FACT-F FS subscale at 6 months (as a mediator for FACT-P only)

*These endpoints will be analysed descriptively.

4.2.1. Process evaluation endpoint

As part of the process evaluation, the following will be assessed descriptively in the SLI arm only:

- Dose of the SLI intervention delivered and received

4.3. Derivation of endpoints

All participants are requested to complete questionnaires at baseline and three-, six- and twelve-months post randomisation. Details of scoring instructions and methods for handling missing item data for questionnaires are provided below. Where documented instructions for dealing with missing data are available these will be used, in the absence of official instructions the half rule will be used (where appropriate). The half rule allows for substitution of the mean of the answered questions for that specific scale (or subscale, for scales with multiple factors) for the missing responses as long as at least half the questions on the given scale / subscale

Copyright © 2007 CTRU, University of Leeds. All rights reserved.

are answered. If the half rule calculation results in a fractional value, then it will be rounded to the nearest whole number. If more than 50% of the items for the specific scale / subscale are missing, the outcome will be assigned as missing. Individual item responses will be summarised for all measures in addition to subscale / total scores where relevant. Unless otherwise specified, all endpoints will be derived after the data has been downloaded into SAS.

4.3.1. Primary endpoints

4.3.1.1. FACT-P Total Score (F50)

The FACT-P is a 39-item participant-reported questionnaire consisting of 5 subscales:

- Physical well-being (PWB)
- Social/family well-being (SWB)
- Emotional well-being (EWB)
- Functional well-being (FWB)
- Prostate cancer (PCS)

For all items, responses can take a value of either 0 “Not at all”, 1 “A little bit”, 2 “Somewhat”, 3 “Quite a bit” or 4 “Very much”. Participants can select “prefer not to answer” for one item within the social/family wellbeing domain.

1. Calculate an individual score for each item as indicated below:

Items(s)	Subscale	Item Score
GP1 – GP7	Physical well-being	4 – item response
GS1 – GS7	Social / family well-being	0 + item response
GE1, GE3 – GE6	Emotional well-being	4 – item response
GE2	Emotional well-being	0 + item response
GF1 – GF7	Functional well-being	0 + item response
C2, P1, P2, P3, P6, P7, BL2, P8	Prostate cancer	4 – item response
C6, P4, P5, BL5	Prostate cancer	0 + item response

2. Calculate a subscale score using the formulas below:

Subscale(s)	Formula	Range
Physical well-being	$\frac{Sum\ of\ individual\ item\ scores\ \times\ 7}{Number\ of\ items\ answered}$	0-28
Social / family well-being	$\frac{Sum\ of\ individual\ item\ scores\ \times\ 7}{Number\ of\ items\ answered}$	0-28
Emotional well-being	$\frac{Sum\ of\ individual\ item\ scores\ \times\ 6}{Number\ of\ items\ answered}$	0–24
Functional well-being	$\frac{Sum\ of\ individual\ item\ scores\ \times\ 7}{Number\ of\ items\ answered}$	0-28
Prostate cancer	$\frac{Sum\ of\ individual\ item\ scores\ \times\ 12}{Number\ of\ items\ answered}$	0-48

3. Calculate a FACT-P total score by summing the PWB, SWB, EWB, FWB and PCS subscale scores. The total score ranges from 0-156

Copyright © 2007 CTRU, University of Leeds. All rights reserved.

4. For the FACT P, higher scores represent better quality of life.

A FACIT measure is considered to be an acceptable indicator of patient quality of life as long as the overall item response rate for the measure is greater than 80%. If the overall item response rate is ≤80%, the total scores for the FACT-P will be set to missing. If there are missing items in a subscale, a valid subscale / total score may still be computed. In line with the FACIT scoring guidelines (https://www.facit.org/scoring), a subscale score can be calculated by taking the mean of the other completed items in the subscale, provided more than 50% of a subscale's items have been answered. If 50% or fewer items in a subscale have been answered, a score should not be calculated for the subscale. In addition, a total score for the FACT-P will only be calculated if all component subscales have valid scores.

The scoring manual for the FACT-P can be found here:
P:\CTRU\Projects\Health_Sciences\Cancer&PallCare\STAMINA\Stats\Notes and Reports\SAP\References\doc22_ScoringFACT-P v4-REVISED.doc

4.3.1.2. FACT-F FS subscale (F50)

The FS subscale is a 13-item section of the FACT-F questionnaire, with items that can take a value of either 0 "Not at all", 1 "A little bit", 2 "Somewhat", 3 "Quite a bit" or 4 "Very much".

1. Calculate an individual score for each item as indicated below:

Items(s)	Subscale	Item Score
HI7, HI12, AN1, AN2, AN3, AN4, AN8, AN12, AN14, AN15, AN16	Fatigue	4 – item response
AN5, AN7	Fatigue	0 + item response

2. Calculate a subscale score using the formulas below:

Subscale(S)	Formula	Range
Fatigue	$\frac{Sum\ of\ individual\ item\ scores\ \times\ 13}{Number\ of\ items\ answered}$	0-52

3. For the FACT-F FS subscale, a higher score represents a lower level of fatigue.

The FACT-F scoring manual can be found here:
P:\CTRU\Projects\Health_Sciences\Cancer&PallCare\STAMINA\Stats\Notes and Reports\SAP\References\doc40_ScoringFACIT-F v4-REVISED.doc

A FACIT measure is considered to be an acceptable indicator of patient quality of life as long as the overall item response rate for the measure is greater than 80%. If the overall item response rate is ≤80%, the FACT-F fatigue subscale score will be set to missing. If there are missing items in a subscale, a valid score may still be computed. In line with the FACIT scoring guidelines (https://www.facit.org/scoring), a subscale score can be calculated by taking the mean of the other completed items in the subscale, provided more than 50% of a subscale's items have been answered. If 50% or fewer items in a subscale have been answered, a score should not be calculated for the subscale.

4.3.2. Secondary endpoints

Copyright © 2007 CTRU, University of Leeds. All rights reserved.

4.3.2.1. FACT-P at three and six-months (F50)

The FACT-P will be assessed at three and six-months and derived as described in Section 4.3.1.1.

4.3.2.2. Physical, Social/Family, Emotional and Function wellbeing, assessed using FACT-P subscales (F50)

The PWB, SWB, EWB and FWB subscales of the FACT-P will be assessed separately and derived as described in Section 4.3.1.1.

4.3.2.3. FACT-F FS subscale at three and six months (F50)

The FACT FS subscale will be assessed at three and six months and derived as described in Section 4.3.1.2.

4.3.2.4. FACT-G at three, six and twelve months (F50)

The FACT-G score will be assessed at three, six and twelve months. The FACT-G score is calculating by summing together the PWB, SWB, EWB and FWB subscale scores which are derived as described in Section 4.3.1.1. The total FACT-G score ranges from 0-108, where a higher score represents a better quality of life.

4.3.2.5. Leisure time physical activity assessed using the Godin questionnaire (F51)

The Godin questionnaire is a simple, 3-item, validated participant-reported questionnaire to measure leisure time exercise. Individuals report how many times on average they have exercised for more than 15 minutes during their free time, in the past 7 days, and whether it was strenuous, moderate, or mild/light exercise.

The total weekly leisure activity score is calculated as:
(9 x number of times strenuous exercise was reported) + (5 x number of times moderate exercise was reported) + (3 x number of times mild/light exercise was reported)

Higher weekly leisure activity scores indicate a higher level of activity.

The Godin scale score is interpreted as either "Insufficiently Active/Sedentary" (score < 14), "Moderately Active" ($14 \leq$ score ≤ 23) or "Active" (score ≥ 24). The Godin score will be reported both as an overall score and using these categorisations at baseline but will be interpreted as a continuous score for further analysis.

There is no questionnaire specific guidance on the handling of missing data for this measure, however in this instance it is not appropriate to use the half-rule. Missing items will therefore be coded as 0.

The scoring manual can be found here:
P:\CTRU\Projects\Health_Sciences\Cancer&PallCare\STAMINA\Stats\Notes and Reports\SAP\References\Godin LTPAQ.pdf

4.3.2.6. Fear of recurrence and psychological distress markers assessed using FCR4 and FCR7 (F53)

The FCR4 and FCR7 scales are used to assess the fear, worry or concern relating to the possibility that cancer will come back or progress. The FCR7 is comprised of 7 items, with the first four of these items making up the FCR4. Each of the first six items are scored using a five-point Likert scale, taking values 1 (not at all), 2 (a little), 3 (sometimes), 4 (a lot) or 5 (all the time). The 7th item is scored from 0 (not at all) to 10 (a great deal).

Responses for items 1-4 are summed to calculate the FCR4 total score. Responses for items 1-7 are summed to calculate the FCR7 total score. The FCR4 total score ranges from 4-20, whilst the FCR7 total score ranges from 6-40. For both the FCR4 and FCR7, higher scores represent a greater fear of recurrence.

There is no questionnaire specific guidance on the handling of missing data for this measure therefore missing data will be imputed using the half-rule for both FCR4 and FCR7.

Copyright © 2007 CTRU, University of Leeds. All rights reserved.

The reference for the FCR4 and the FCR7 can be found here:
P:\CTRU\Projects\Health_Sciences\Cancer&PallCare\STAMINA\Stats\Notes and Reports\SAP\References\FCR4_FCR7.pdf

4.3.2.7. Adverse effects of ADT assessed using trial specific ADT Symptom Index (F57)

The ADT Symptom Index consists of 16 items which measure the severity of symptoms experienced by participants that may be related to receiving ADT. The responses for each item will be summarised descriptively and a total score will not be derived. No imputation for missing data will take place.

4.3.2.8. Dose delivered and received

Dose monitoring is drawn from participant intervention logbooks, session attendance reports, swipe card data and progress/summary reports produced by Clinical Exercise Specialists (CES).

Data from the exercise sessions will be recorded on intervention logbooks by the appropriate CES. The SHU team will annotate the SLI logbooks to aid the data entry process for the CTRU team. Details of the possible annotations can be found in the SOP for logbook data entry, linked here: P:\CTRU\Projects\Health_Sciences\Cancer&PallCare\STAMINA\Stats\Notes and Reports\Logbook documents\SOP_Logbook data entry_v3_finalise.docx.

Additionally, once entered into MACRO, data from logbooks will be cross-checked for validity against session attendance reports and swipe card data by the statistician. Dates from each of the three data sources will be compared to determine whether an exercise session was supervised or unsupervised.

4.3.2.9. Other derivations

Body Mass Index (BMI) will be derived by dividing the patient's weight in kilograms by the square of their height in metres.

A Body Shape Index (ABSI) (26) will be calculated using the following formula:

$$ABSI = \frac{Waist\ Circumference}{BMI^{2/3} \times Height^{1/2}}$$

Musculoskeletal and cardiorespiratory comorbidities will be assessed as part of the moderator analysis (Section 7.14.1). The comorbidities listed on the health screening questionnaire (F03) will be the source data.

For musculoskeletal comorbidities, participants will be classified into one of the follow categories:

- 0 - No musculoskeletal comorbidities
- 1 - Musculoskeletal comorbidities, including:
    - Arthritis (including rheumatoid)
    - Significant musculoskeletal injury
    - Chronic joint/musculoskeletal complaint

For cardiorespiratory comorbidities, participants will be classified as follows:

- 0 - No cardiorespiratory comorbidities
- 1 – Hypertension and/or DM with no other cardiorespiratory comorbidities
- 2 – At least one of the below cardiorespiratory comorbidities (with or without hypertension and/or diabetes):
    - Asthma
    - Bronchitis
    - Any COPD
    - Aneurysm
    - Embolism

Copyright © 2007 CTRU, University of Leeds. All rights reserved.

- Atrial fibrillation
- Stable angina
- Unstable angina
- Recent myocardial infarction
- Pacemakers
- Any other significant cardiovascular disease

Disease status (as collected on F05) will also be categorised in an alternate way for the moderator analysis. Participants will be categorised in the following way:

- 1 – Localised disease, including:
    - Localised disease T1/2, N0, M0
- 2 – Locally advanced, including:
    - Locally advanced (node negative) T3/4 N0 M0
    - Locally advanced (node positive) Tx N1 M0
- 3 – Hormone sensitive, including:
    - Metastatic hormone sensitive
- 4 – Castrate resistant, including:
    - Non-metastatic castrate-resistant
    - Metastatic castrate-resistant

4.4. Missing data

The amount of missing data will be summarised for each endpoint described in Section 4.1 and 4.2. This will include the type (measure-level, subscale-level and item-level) of missing data for each questionnaire. Reasons for missingness (where available) will be reported.

4.4.1. Investigation of missing data pattern

The number and proportion of participants with missing outcome data will be presented overall, by treatment arm, by baseline characteristics and by baseline characteristics between arms and by site. Where available, reasons for missing data will be presented (death, withdrawal etc.). A distinction will be made between missing data due to questionnaires not returned and those with invalid scores/subscale scores due to missing individual items. The missing data percentages for questionnaires not returned will take into account forms not expected for reasons such as a participant's withdrawal from the trial. If an endpoint is missing <5% of data, a complete case analysis will be performed. If an endpoint is missing ≥5% of data and the reason for missingness is not due to death then multiple imputation will be used; if the reason for missingness is due to death, no imputation will be performed.

Data will be examined to assess whether data is missing at random (MAR), missing completely at random (MCAR) or missing not at random (MNAR). The missing data pattern of the following variables will be investigated:

- Missing outcome data (including corresponding baseline scores)
- Missing participant-level covariate data measured at baseline
    - Age
    - Duration on ADT
    - Receiving chemotherapy and/or novel ARI
    - Receiving radiotherapy
    - Preferred method of outcome data completion
- Missing CES-level covariate data
    - Missing CES ID

To determine whether the pattern of missingness is monotone or non-monotone, the pattern and frequency of the missingness will be explored as per the table below and as recommended by Carpenter & Smuk (27).

Copyright © 2007 CTRU, University of Leeds. All rights reserved.

Pattern	Var 1	Var 2	Var 3	Outcome	Number
1	X	O	O	O	N (%)
2	X	X	O	O	N (%)
3	X	X	X	O	N (%)
…	…				

As a predefined standard for all outcomes, if the data is not considered to be MCAR and >5% of observations are missing/invalid and would therefore not contribute to the final analysis model, imputation will be used (28). Missing outcome data due to participant death will not be imputed in the analysis models.

4.4.2. Method of imputation

Patterns of missing questionnaire data will be explored to identify whether the data is MAR, MNAR or MCAR, as above. If the data is MCAR, complete case analysis will be used. If the data is MAR or MNAR, questionnaire data / scores will be estimated using multiple imputation for all participants. Multiple imputation via pattern-mixture modelling method will be used to impute missing values for each of the questionnaire scores and allows for the clustered nature of the data to be taken into account, as well as the pattern of missingness (29). The model uses the average differences in response between baseline and time points of 3, 6 and 12 months, along with the difference between treatment arms.

Each of the two primary outcomes will be evaluated for missing data imputation separately. Pattern mixture models will use linear mixed models to evaluate whether each missing data pattern predicts the outcome variables (FACT-P total score, FACT-F FS subscale) or interacts with time (time points 0, 1, 2 and 3 representing baseline, 3, 6 and 12 months respectively) to predict changes in the outcome variables over time. The dependent variables will be the FACT-P total score and the FACT-F FS subscale, and the predictors will be the missing data patterns (missing FACT-P total score/FACT-F FS subscale at 3 months only, missing FACT-P total score/FACT-F FS subscale at 6 months only, missing FACT-P total score/FACT-F FS subscale at 12 months only, missing FACT-P total score/FACT-F FS subscale at 3 and 6 months, missing FACT-P total score/FACT-F FS subscale at 3 and 12 months, missing FACT-P total score/FACT-F FS subscale at 6 and 12 months, no missing FACT-P total score/FACT-F FS subscale score at any time point). We expect there to be no missing data at baseline.

5. Populations

5.1. Screening population

The screening population will consist of all patients who were screened for entry into the STAMINA trial. If patients were known to have been screened but no screening form was completed (i.e. only summary information was obtained from sites) then these patients will be included in this population.

5.2. Eligibility

Patients meeting all of the inclusion criteria and none of the exclusion criteria prior to randomisation will be eligible to take part.

Inclusion criteria

- Men with prostate cancer on ADT or due to start ADT within the next 12 weeks*
- Willing to provide informed consent.

** where a man is due to start Taxane based chemotherapy in conjunction with ADT, an initial health questionnaire will be conducted but inclusion in the trial will be deferred until after completion of the course of chemotherapy (typically 18-20 weeks). Baseline assessments will be repeated before randomisation where necessary.*

Exclusion criteria

Copyright © 2007 CTRU, University of Leeds. All rights reserved.

- Absolute contraindication to exercise as defined by clinical guidance e.g. ACPICR standards (30)
- Uncontrolled hypertension[#]
- Uncontrolled diabetes mellitus
- Recent myocardial infarction (within past 6 months)
- Unable to provide informed consent (e.g. lack of capacity)
- Unstable bony metastases unresponsive to treatment
- Unable to complete study assessments
- Participation in other lifestyle intervention trial for PCa[~]
- Estimated life expectancy of less than 12 months for reasons unrelated to PCa diagnosis
- Involvement in previous STAMINA work packages or PPI panel

[#]Uncontrolled hypertension is defined for the study as a mean systolic BP>180 mmHg or diastolic BP>110 mmHg. Men who are found to have uncontrolled hypertension will be referred back to their GP.

~Participation in another study will not necessarily exclude a patient from participation. CTRU should be notified of any potential conflicting studies to facilitate a review of the feasibility of co-enrolment by the Chief Investigator and Trial Management Group. The review will consider methodological impact and participant burden.

5.3. Intention to treat population

The intention-to-treat (ITT) population will consist of all randomised participants, regardless of any non-compliance with their assigned treatment. Participants will be grouped and analysed according to their randomised treatment. Participants found to be ineligible after randomisation will be included in this population. Participants who withdraw consent for use of their data, or for whom written informed consent has not been obtained (and implied consent cannot be assumed), will be excluded.

5.4. CACE population

Complier Average Causal Effect (CACE) analysis (31) can be used to estimate the effect of an intervention amongst compliant participants. The CACE is defined as the average effect of treatment in the compliers. Compliers are defined as participants who would have received an effective dose of the intervention had it been offered. Analysis takes non-adherence to the intervention into account, comparing treatment received rather than treatment allocated.

The CACE approach assumes four types of compliance status: compliers, never takers, defiers and always takers:

1. Always-takers will always receive the intervention irrespective of their allocation
2. Never-takers will never receive the intervention irrespective of their allocation
3. Compliers receive the intervention if and only if they are allocated to the SLI arm
4. Defiers receive the intervention if and only if they are allocated to the OUC arm

The CACE population will therefore consist of all randomised participants. Compliance with the SLI intervention will be defined as the proportion of prescribed exercise sessions completed out of all prescribed exercise sessions. Each participant allocated to SLI is expected to complete 2 sessions per week during the first 12 weeks of the intervention (24 sessions in total). Over the remainder of the 12-month intervention period, the prescribed number of sessions is agreed between each participant and their CES at progress reviews which take place at regular intervals. The number of prescribed exercises sessions can therefore vary by participant. Participants who complete 21 or more prescribed exercise sessions, including at least 18 during the first 12 weeks of intervention delivery, will be classed as compliers.

6. Data Handling

6.1. Data monitoring

Where possible, data will be monitored for quality and completeness by the CTRU, using established

Copyright © 2007 CTRU, University of Leeds. All rights reserved.

verification, validation and checking processes. Missing data, except individual data items collected via the postal/online questionnaires, will be chased until they are received, confirmed as not available, or when the trial is at analysis. If questionnaires are not returned on time, up to three reminders may be sent via post, email, or text depending on the participants preferred method of contact. Every effort will be made to ensure as much data as possible are available for analysis, and that reasons for unobtainable data are obtained. Any issues with data collection will be discussed at Project Delivery Meetings and, if appropriate, at Trial Management Group meetings.

6.2. Data validation

Data management will conduct initial validation of trial data collection forms in accordance with work instructions developed for the trial. Data relating to eligibility and consent will be checked upon receipt so that discrepancies are raised and resolved in a timely fashion. Validation checks, incorporated into the trial database will identify errors at the time of data entry. Discrepancies will be raised with sites for resolution. Key data items relating to the primary endpoints will be 100% checked by the Data Manager or delegate and are defined in Appendix 1.

MACRO data will be downloaded and converted into SAS datasets via the CTRU SAS data views process. Participant reported outcome data will be downloaded from the REDCap platform.

Prior to the final analysis, the statistician will create SAS programs to validate the data and check for any spurious, inconsistent or missing data. This will include, but is not restricted to checking:

- Eligibility of all randomised participants;
- Consistency between stratification factors provided at screening / baseline / randomisation;
- Sequential dates;
- Outliers and unusual data values;
- Missing data;
- Data that is inconsistent between forms.

Any suspicious or inconsistent data identified via these checks will be recorded in the Statistician's Trial Folder on the CTRU P: drive and the Data Manager will be notified. The Data Manager or their delegate will check inconsistencies against the relevant forms. If there has been an error in data input causing such inconsistencies this will be corrected on the database. If there has been no error in data input, and if the query relates to data that can be accessed by a researcher, clinician, or clinical exercise specialist, then a query requesting clarification will be sent to the relevant personnel. Details of any corresponding data changes will be documented by the Data Manager and stored in the Statistician's Trial Folder on the CTRU P: drive.

Additional data relating to participant eligibility, unsupervised/supervised gym sessions and Index of Multiple Deprivation (IMD) will be transferred by the research team at Sheffield Hallam University (SHU) to the CTRU via the Secure Transfer service. Validation of this data will be conducted in accordance with the SLI Logbook SOP (P:\CTRU\Projects\Health_Sciences\Cancer&PallCare\STAMINA\Stats\Notes and Reports\Logbook documents). Prior to importing the data into SAS, the trial statistician will check the data to ensure only that required for analysis (and for STAMINA participants) has been transferred. This data will then be imported into SAS by the trial statistician.

7. Data analysis

Statistical analysis is the responsibility of the CTRU Trial Statistician. This SAP has been written in accordance with current CTRU standard operating procedure (SOP) S04. Any changes to the analysis plan outlined in this section, and reasons for the changes will be documented in the amendment log (TEM20) and filed with this analysis plan.

All analysis will be carried out by the trial statistician using the most recent version of SAS available at the time

Copyright © 2007 CTRU, University of Leeds. All rights reserved.

of analysis unless stated otherwise. The version will be recorded in the SAS program header of all programs. Analyses will not be conducted blind to randomised allocation. Analyses which adjust for the minimisation factors will use data as provided on the 24-hour registration / randomisation system. Subgroup analyses (if applicable) will use data as provided on F07 for the relevant variable e.g. age.

7.1. General calculations

All analyses will be conducted on the ITT population as defined in Section 5.3, except for summaries of screening data which will be conducted on the screening population. Tables will present summary statistics by treatment arm and overall, unless otherwise specified. Means, standard deviations, medians, interquartile ranges (IQRs) and ranges will be presented for continuous variables, with counts and percentages shown for categorical/binary variables. Means, medians, quartiles, ranges and percentages will be rounded to 1 decimal place (or 1 significant figure for numbers less than 1), whilst standard deviations will be rounded to 2 decimal places (or 2 significant figures for numbers less than 1). P-values will be rounded to 3 decimal places (with those less than 0.001 displayed as <0.001). Parameter estimates, standard errors, ICCs and 95% confidence intervals will be reported to 2 decimal places (or 2 significant figures for numbers less than 1). Unless otherwise stated, all percentages will be calculated using the total number of participants with data available as the denominator and the number of participants with missing values will be presented. Hypothesis testing for the primary endpoints will be two-sided and use an adjusted alpha of 0.02768. All other hypothesis testing will be two-sided at the 5% significance level.

Template tables are available in a separate document, saved here:
P:\CTRU\Projects\Health_Sciences\Cancer&PallCare\STAMINA\Stats\Notes and Reports\SAP\Tables and Figures.

7.2. Trial summary

7.2.1. Milestones

The timelines for the trial, including the recruitment period, follow-up period, and data cut-off point for the analysis will be summarised.

7.2.2. Populations

The number of patients included in the screening, eligibility, ITT and CACE populations will be summarised overall and by arm.

7.2.3. Participant summary

A CONSORT (32) flow diagram will be used to summarise the course of participants throughout the trial, this will include the number of participants:

- Screened
- Referred (with reasons for non-referral where available)
- Registered
- Not registered (with reasons for non-registration where available)
- Randomised
- Not randomised (with reasons for non-randomisation where available)
- Allocated to each trial arm
- Lost to follow-up or withdrawn (with reasons where available)
- Analysed in the ITT population

Screening and recruitment will also be summarised by site, and recruitment per month (overall and by arm) will be displayed graphically.

Copyright © 2007 CTRU, University of Leeds. All rights reserved.

7.3. Characteristics

7.3.1. Patient screening characteristics

The following characteristics collected on F01 will be summarised using frequencies and summary statistics for the overall screened population:

- Age
- Ethnicity
- Identification method
- ECOG status
- Type of ADT
- Disease status

7.3.2. Participant baseline characteristics and clinical measurements

The following characteristics and clinical measurements collected on F02, F03, F04, F05, F06, F07 and F99 will be summarised for the ITT population using frequencies and summary statistics, overall and by arm:

1. Age at registration (derived from date of birth) * (F02)
2. Gender * (F02)
3. Preferred method of questionnaire completion (F02)
4. Ethnicity * (F03)
5. Index of Multiple Deprivation (IMD) Deciles*
6. Retired? (F03)
7. Current health status (F03)
8. Current functional capacity (F03)
9. Registered disabled (F03)
10. Current level of fitness (F03)
11. Moderate intensity exercise (F03)
12. Resting heart rate (F03)
13. Resting blood pressure (F03)
14. Method of blood pressure measurement (F03)
15. Smoking status and details (F03)
16. Alcohol consumption levels (F03)
17. Any consultation with doctor in last 6 months (F03)
18. 1st degree relative diagnosed with angina or heart attack (F03)
19. Taking any ARIs (F03)
20. Form of ADT (F03)
21. Length of time on ADT (F03)
22. Due to receive chemotherapy or radiotherapy to treat prostate cancer in next 3 months (F03)
23. Receiving any other treatments for prostate cancer (F03)
24. Comorbidities/other conditions (F03)
25. Cardiorespiratory comorbidities (as defined in Section 4.3.2.9)
26. Musculoskeletal comorbidities (as defined in Section 4.3.2.9)
27. COVID-19 (F03)
28. Received COVID-19 vaccine (F03)
29. Other medical conditions (F03)
30. Access to technological devices (F03)
31. Weight (kg) (F04)
32. Height (m) (F04)
33. BMI (derived as in Section 4.3.2.9)
34. A Body Shape Index (ABSI) (26) (derived as in Section 4.3.2.9)
35. Waist circumference (cm) (F04)
36. Hip circumference (F04)
37. Chair sit-to-stand test in 30 seconds (F04)
38. Disease status (F05)

Copyright © 2007 CTRU, University of Leeds. All rights reserved.

39. Alternative disease status (as defined in Section 4.3.2.9)
40. Form of ADT patient is receiving * (F05)
41. Resting systolic blood pressure (F05)
42. Resting diastolic blood pressure (F05)
43. Length of time ADT has been prescribed (F05)
44. Any other planned treatment for prostate cancer (F05)
45. Eligibility criteria (F06)
46. Age at randomisation in years (derived from date of birth) * (F07)
47. Stratification factors (F07 + F99)

*These characteristics will also be compared (where possible) for the screened, registered and randomised populations to check representativeness.

The stratification factors (age, duration on ADT, receiving chemotherapy and/or novel ARI, receiving radiotherapy) collected on F99 will be summarised using frequencies overall and by arm. If applicable, the number of participants randomised under incorrect stratification factors will be summarised.

For randomised participants, summaries of the eligibility criteria collected on F06 alongside the number of participants with missing data or who do not fulfil the eligibility criteria (if applicable) will be presented overall and by arm.

No statistical testing will be carried out for differences in baseline characteristics between the arms.

7.3.3. Baseline outcomes

Participant reported outcome measures (PROMs) measured at baseline will be reported by overall and by arm. These outcomes include:

1. FACT-P (F50)
2. FACT-F FS subscale(F50)
3. FACT – PWB (F50)
4. FACT – SWB (F50)
5. FACT – EWB (F50)
6. FACT – FWB (F50)
7. FACT-G (F50)
8. Godin Leisure-Time Score (F51)
9. FCR4 (F53)
10. FCR7 (F53)
11. ADT symptom index (F57)

Completion rates, the method of completion (online vs. postal) of participant reported outcome measures will be summarised overall and by arm. Method of completion vs. preferred method of completion indicated at baseline will also be summarised overall and by arm. The number of participants requiring assisted completion of questionnaires (via telephone) will be described if applicable. The number of items of missing data per participant reported outcome measure will also be reported.

7.4. Intervention delivery

Summaries in this section will be presented for participants randomised to the SLI arm only.

7.4.1. Participant level summaries

A flow diagram illustrating the number of participants randomised to SLI, number commencing SLI, number of supervised SLI sessions attended and number withdrawing or discontinuing will be presented, along with the timing of withdrawal or discontinuation. Summary tables presenting this information by NH gym will also be reported.

Copyright © 2007 CTRU, University of Leeds. All rights reserved.

7.4.2. Supervised sessions

The SLI intervention will be delivered to participants by trained CES. SLI sessions will be delivered in four phases:

Phase 1: Twice weekly supervised sessions for 12 weeks post-randomisation
Phase 2: Negotiated supervision from 3-6 months post-randomisation
Phase 3: Negotiated supervision from 6-9 months post-randomisation
Phase 4: Negotiated supervision from 9-12 months post-randomisation

Participants requiring a run-in period will follow a ramped approach to exercise, tailored to their capabilities.

Participants will undergo progress reviews with their CES at intervals throughout receipt of the intervention. These progress reviews will take place at week 12, week 19, month 6, week 32, month 9, week 45 and month 12. Various aspects of the participant's experience will be discussed, and plans will be set out for further prescribed exercise sessions.

Participants will also undergo submaximal exercise tests at baseline, week 12, month 6 and month 12 to test their fitness levels.

The following will be summarised at a participant level using frequencies and summary statistics (where relevant):

- Details of participant induction, including:
  - Number and proportion of participants completing an induction
  - Number and proportion of participants where blood pressure recommendations from referral information were reinforced
  - Distance from participant's home to gym (in miles)
  - Method of participant's transport to NH gym
- Details of intervention delivery timings, including:
  - Time between randomisation and induction session (in days)
  - Time between randomisation and the first session (in days)
  - Time between induction and the first session (in days)
- Details of run-in period, including:
  - Number and proportion of participants having a run-in period
  - Length of run-in period (in days)
  - Whether the prescribed frequency of sessions changed in the run-in period
- Number of sessions attended by participants

The following will be summarised at a participant level using frequencies and summary statistics (where relevant):

- Number and proportion of participants who received at least one session (i.e. commenced SLI)
- Number of supervised sessions attended per participant
- Number and proportion of participants attending at least one remotely delivered session
- Number and proportion of participants attending at least one group delivered session
- Number of group delivered sessions attended per participant
- Number of one-to-one delivered sessions attended per participant

The following will be summarised at a session level by phase, timepoint post-randomisation (week 1 to 12, month 3-6, month 6-9, month 9-12 and post month 12) and overall using summary statistics:

- The total number of supervised sessions attended per participant

The following will be summarised at a session level by timepoint and overall using frequencies and summary statistics (where relevant):

- Details of supervised sessions, including:

Copyright © 2007 CTRU, University of Leeds. All rights reserved.

    - Number and proportion of sessions attended
    - Reasons for non-attendance at sessions
    - Number and proportion of sessions where an exercise prescription was altered (overall only)
    - Reasons for alteration of exercise prescription
    - Time of exercise sessions (AM/PM)
    - Number and proportion of one-to-one vs. group delivered sessions
    - Number and proportion of face-to-face vs. remotely delivered sessions
    - Number and proportion of sessions where aerobic exercise was performed
    - Type of aerobic exercise performed
    - Number and proportion of sessions where resistance exercise was performed
    - Type of resistance exercise performed

The following will be summarised at a participant level and by timepoint overall using frequencies and summary statistics (where relevant):

- Details of submaximal exercise testing, including:
    - Number and proportion of sessions where test was performed
    - Reason test was not performed
    - Type of equipment test was performed on
    - Duration of test (in minutes)
    - RPE at test termination
    - Reason for test termination
- Details of progress reviews, including:
    - Number and proportion of face-to-face vs. remotely delivered sessions
    - Number and proportion of sessions where Boom and Bust was discussed (week 12 only)
    - Number and proportion of sessions where rewards were discussed (week 12 only)
    - Number and proportion of sessions where social support was discussed (week 12 only)
    - Number and proportion of sessions where habit formation was discussed (week 12 only)
    - Number and proportion of sessions where exercise plans were discussed (week 12 only)
    - Number and proportion of sessions where goal setting was discussed (week 12, month 9 only)
    - Number and proportion of prescribed sessions in phase 2 (week 12 only)
    - Number and proportion of sessions where motivation was discussed (week 19 only)
    - Number and proportion of prescribed sessions in phase 3 and 4 (month 6 only)
    - Number and proportion of sessions where reflection was discussed (month 6 only)
    - Number and proportion of sessions where imagery was discussed (month 6 only)
    - Number and proportion of sessions where setbacks were discussed (week 32 only)
    - Number and proportion of sessions where achievements were discussed (week 45 only)
    - Number and proportion of sessions where action planning was discussed (month 12 only)

The following will be summarised at a session level using summary statistics for each phase and overall:

- Number of sessions scheduled
- Number of sessions attended
- Aerobic exercise intensity during sessions
- Aerobic exercise duration during sessions
- Resistance exercise intensity during sessions
- Resistance exercise volume during sessions

7.4.3. Unsupervised sessions

The following will be summarised at a participant level by phase, timepoint post-randomisation (week 1 to 12, month 3-6, month 6-9, month 9-12 and post month 12) and overall using summary statistics:

- The total number of unsupervised exercises sessions attended per participant.
- The total number of exercise sessions (both supervised and unsupervised) attended per participant

Copyright © 2007 CTRU, University of Leeds. All rights reserved.

7.4.4. Clinical Exercise Specialist (cluster) level summaries

The STAMINA trial design ensures that any clustering of participant outcomes due to the way in which the CES delivers the intervention is accounted for. Participants will be assigned to a CES cluster based on the following rules:

1. Where participants have ≥50% of their supervised sessions delivered by one CES, participants will be assigned to that CES.
2. Where participants do not have one CES delivering ≥50% of their supervised sessions, participants will be assigned to the CES that has delivered the highest number of their sessions.
3. In the case of equal delivery (i.e. a participant has seen multiple CES who have all delivered the same number of supervised sessions to that participant), participants will be assigned to the CES that delivered their first supervised session.

Two sensitivity analyses will be conducted for the primary endpoints which explore different clustering structures based on the following rules:

1. Participants will be assigned to a cluster according to the NH gym they attended to explore the possibility of a "team effect" of intervention delivery.
2. Participants will be assigned to a cluster according to the CES that delivered their first supervised exercise session to explore the possibility that the first CES "sets the tone" of a participant's experience with SLI.

The following summaries will be presented overall and by NH gym:

- Number of CES who delivered at least one SLI session to a participant (number of clusters)
- Number and proportion of participants assigned their cluster according to each rule above
- Mean and range of participants per CES (cluster size)
- Number and proportion of participants who had SLI delivered by more than one CES
- Number, mean and range of CES delivering the intervention per participant

7.5. Withdrawals (F15, F16)

The number and proportion of participants who requested a withdrawal and reasons (where available) will be summarised overall, and by arm. The type of withdrawal will also be summarised: withdrawal from the SLI intervention (for those randomised to SLI), from follow-up appointments, from questionnaire completion or from collection of data from medical records. The timing of participant withdrawals (in months and by time point) will be summarised overall, by arm and by type of withdrawal. A line listing summarising the details of all withdrawals will also be presented by participant.

The number and proportion of participants clinically withdrawn from the SLI intervention the reasons (where available) and timing of withdrawal (in months and by time point) will be summarised for those in the SLI arm. A line listing summarising the details of all clinical withdrawals will also be presented by participant.

7.6. Deaths (F14)

The number and proportion of participant deaths, cause of death (where available) and timing of death post-randomisation (in months) will be summarised overall and by arm. A line listing summarising the details of all deaths will also be presented by participant.

7.7. Unblinding (F21)

The number of and proportion of participants for whom a researcher became aware of their randomised allocation and the number of times this occurred per participant will be summarised overall, by arm and by site. The allocation of the participant, time-point of unblinding and how the researcher was unblinded will also be summarised by overall, by arm, and by site. A line listing summarising the details of all unblindings will also be presented by participant.

Copyright © 2007 CTRU, University of Leeds. All rights reserved.

7.8. Protocol violations and deviations (F17)

The number and proportion of participants who were randomised without consent or who were found to breach the trial eligibility criteria (protocol violation) after randomisation, the eligibility criteria breached, timing of identification (in months), will be summarised overall, by arm and by site. A line listing summarising the details of all protocol violations (including action taken, whether the event was medically significant and linked to an RUSAE) will be presented by participant.

The number and proportion of participants for whom any other type of protocol deviation was reported and the timing of identification (in months) will be summarised overall, by arm and by site. A line listing summarising the details of all protocol deviations (including action taken, whether the event was medically significant and linked to an RUSAE) will be presented by participant.

7.9. Primary endpoint analysis

The analysis of the primary endpoints will be carried out on the ITT population. The primary endpoints are the FACT-P total score and the FACT-F FS subscale at 12 months post-randomisation. The corresponding estimands and derivations of the endpoints are defined in Section 4.1.1 and Section 4.3.1. Missing data will be handled as per Section 4.4. Summary statistics for each endpoint will be presented overall, by arm and by time point.

The primary analysis of the FACT-P total score will be undertaken using partially nested mixed-effects linear regression. The analysis model will adjust for the stratification factors as defined in Section 3.4 (age will be treated as a continuous variable) as well as the baseline FACT-P total score, site, and type of site (cancer vs. non-cancer). To account for potential clustering of participant outcomes due to CES delivery, the SLI arm will also be adjusted for the CES (as defined in Section 7.4.4). This model confines the random effect to the SLI arm and does not require artificial clustering in the optimised usual care arm. We will check the assumption of homoscedastic participant errors across the trial arms and if differences are apparent, a heteroskedastic participant errors model will be used instead (33).

Prior to analysis, we will also produce summaries at the cluster level as per Section 7.4.4 to explore mean cluster size. If the mean cluster size in the SLI arm is five or above, analysis will proceed using the partially nested model as described above. If the mean cluster size in the SLI arm is below five, a generalised linear model, adjusted for the same covariates as above minus the CES in the SLI arm, will be considered (34).

Appropriate model assumption checks will be conducted, including deviance residuals and influence statistics (leverage). Scatter plots will be produced for each endpoint with trend lines to visualise differences in outcome distributions between arms. The final estimated treatment effect, covariate estimates, ICC in the SLI arm and associated standard errors, p-values and 97.232% confidence intervals will be presented.

The primary analysis of the FACT-F FS subscale will be conducted in the same way as described for the FACT-P total score, adjusting for the same covariates but replacing baseline FACT-P total score with the baseline FACT-F FS subscale.

7.9.1. Sensitivity Analysis

Two sensitivity analyses will be conducted for each of the primary endpoints as per Section 7.4.4 to explore the impact of different clustering structures. These analyses will be conducted in the same way as described for the primary endpoints.

7.9.2. CACE Analysis

In addition to the ITT analysis, a Complier Average Causal Effect analysis will be undertaken on the 12-month primary endpoints to estimate the effect of SLI amongst compliant participants. This analysis will be considered if more than 20% of SLI participants do not receive the intervention as intended (defined in Section 5.4). Parameter estimates for the intervention effect can then be compared with those from the ITT analysis to aid understanding of the effect of the intervention.

Copyright © 2007 CTRU, University of Leeds. All rights reserved.

7.9.2.1. Methods for handling departures from randomised intervention

There are 5 assumptions that need to be fulfilled before CACE analysis can be employed (35):

1. Outcomes for each participant are independent of the outcomes for other participants, known as the Stable Unit Treatment Value assumption.
2. There is a monotonic relationship between treatment assignment and treatment receipt. Therefore, there are no participants for whom assignment to treatment actually reduces the likelihood of receiving treatment (e.g. no defiers, no participants who would take the intervention if it was not offered).
3. Offering treatment to participants in the intervention arm induces at least some participants to receive the treatment, so the compliance rate is not zero.
4. Assignment to treatment is random.
5. Random assignment to treatment does not affect the outcomes of participants who do not comply with the treatment, an assumption known as the exclusion restriction.

The outcome for each participant depends only on their own treatment assignment and not the assignment of any other participant, however, CES standard error will be used to account for clustering, so we can assume the Stable Unit Treatment Value assumption stands for the STAMINA population.

As the SLI was not delivered to participants assigned to the OUC arm, we can assume there were no “defiers” in the OUC arm. Due to the randomness of the allocation, we can also assume that there was the same lack of “defiers” in the SLI arm.

We can assume that at least some participants in the SLI arm complied with SLI, i.e. the rate of compliance is not zero. Due to the randomness of the allocation, we can assume there is the same rate of non-compliance in the OUC arm.

For the fourth assumption, the allocation was random in nature due to the trial design and use of a randomisation algorithm incorporating minimisation.

For the final assumption, the exclusion restriction, we assume that only the randomised treatment allocation has an effect on the outcome and the effect of assignment is mediated by treatment exposure, which can be assessed by including covariates that predict “engagement” with the intervention, i.e. participant attended the minimum required number of prescribed sessions.

A mixed linear regression model will be used as the CACE model. Participants in the SLI arm will have direct observation of compliance. Compliance will be estimated in the OUC arm using maximum likelihood estimation via the expectation-maximisation algorithm estimation (ML-EM) (36, 37). Two dummy variables will be used to indicate compliance status. For the SLI arm, compliers (as defined in Section 5.4) will be assigned 1 for dummy class variable 1 and 0 for dummy class variable 2 and non-compliers will be assigned 0 for dummy class variable 1 and 1 for dummy class variable 2. For participants in the OUC arm, both dummy class variables will be assigned as 1 (37).

The intervention effect will be fixed at 0 for non-compliers (under the exclusion restriction assumption). Baseline covariates (as described in Section 7.9 for the primary analysis) that are potential predictors will be included in the model to increase the precision of CACE estimates.

7.10. Secondary endpoint analysis

All participant reported secondary endpoints (Table 1) will be analysed using the same approach as described in Section 7.9 for the primary endpoints. Missing data will be handled as per Section 4.4. Summary statistics for each endpoint will be presented overall, by arm and by time point. Models will adjust for the stratification factors as defined in Section 3.4 (age will be treated as a continuous variable) as well as the baseline score of the relevant outcome measure, site, and type of site (cancer vs. non-cancer). Appropriate model assumption

Copyright © 2007 CTRU, University of Leeds. All rights reserved.

checks will be conducted, including deviance residuals and influence statistics (leverage). Scatter plots will be produced for each secondary endpoint with trend lines to visualise differences in outcome distributions between arms. The final estimated treatment effect, covariate estimates, ICC in the SLI arm and associated standard errors, p-values and 97.232% confidence intervals will be presented.

Table 1: *Table showing each participant reported secondary endpoint and the time points at which they are collected*

Endpoint	Time point
FACT-P total	3, 6m
FACT-F FS subscale	3, 6m
Physical, Social/Family, Emotional, Functional wellbeing FACT subscales	3, 6, 12m
FACT-G total score	3, 6, 12m
Godin questionnaire score	3, 6, 12m
FCR4 and FCR7	3, 6, 12m

7.11. ADT symptom index (F57)

Responses to items on the ADT symptom index will be summarised using frequencies and summary statistics and presented overall and by arm, at each of the follow-up timepoints (3, 6 and 12 months). Missing data items will not be imputed.

7.12. Physical (F8, F9, F10)

Measures of the participants' functional capacity and body composition (including blood pressure, chair sit-to-stand, waist and hip circumference, weight and ABSI) will be summarised using frequencies and summary statistics and presented overall and by arm, at each of the follow-up timepoints (3, 6 and 12 months). Missing data items will not be imputed.

7.13. Safety (F18)

The number and proportion of participants with a reported Related/Unexpected Serious Adverse Event (RUSAE) and the number reported per participant will be summarised overall and by arm. A line listing summarising the details of all RUSAEs including onset date, main diagnosis/symptom, associated symptoms, RUSAE description, location where RUSAE started, seriousness criteria, outcome, relevant medical history and action taken will be presented by participant.

7.14. Exploratory analyses

7.14.1. Moderator analysis

For the primary endpoints, potential moderators (38) of the treatment effect will be explored both in terms of their overall relationship with outcome (main effect, predictive ability), and whether they moderate the overall treatment effect (interaction effect between treatment x potential moderator) for the following baseline characteristics:

- A Body Shape Index (ABSI)*
- IMD decile (1, 2…10) **
- Cardiorespiratory comorbidities (F03) (no conditions, hypertension or diabetes, all other cardiorespiratory comorbidities)**
- Musculoskeletal comorbidities (F03) (no conditions, musculoskeletal comorbidities)**
- Disease status (F05) (localised disease, locally advanced, hormone sensitive, castrate resistant)**
- Chair sit-to-stand (F04)*
- Age (F99)*

Copyright © 2007 CTRU, University of Leeds. All rights reserved.

- Second line treatment (F99) (yes or no)**
- Radiotherapy (F03) (yes or no)**
- Length of time on ADT (F99) (≤ 12 weeks OR > 12 weeks)**
- FACT-F FS subscale (F50) (as a moderator for FACT-P only)

Characteristics marked with * will be included as continuous variables. Those marked with ** will be included as categorical variables using the levels indicated above. Summary statistics of each proposed moderator will be tabulated and presented in a box plot for each primary endpoint to explore their main effect. The relationship between each outcome variable and moderator will be plotted graphically (box plots for categorical moderators, scatter plots with trend lines for continuous moderators). Each proposed moderator will be included in the main analysis model for each primary endpoint (one at a time), both as a covariate on its own and as an interaction with the randomised treatment. Appropriate model assumption checks will be conducted, including deviance residuals and influence statistics (leverage). The final estimated treatment effect, covariate estimates, ICC in the SLI arm and associated standard errors, p-values and 97.232% confidence intervals will be presented. Irrespective of whether the main effect is significant, if the interaction is, the characteristic will be said to moderate the treatment effect.

7.14.2. Mediator analysis

For the primary endpoints, the following variables will be considered as potential mediators:

- Godin activity level
- ABSI
- FACT-F FS subscale at 6 months (as a mediator for FACT-P only)

Summary statistics of each potential mediator will be tabulated by arm for each primary endpoint.

An improvement in the Godin activity score could indicate an improvement in quality of life and reduced fatigue, which may correspond to improvements in both FACT-P and FACT-F FS scores. Lower levels of adiposity as measured by the ABSI may similarly correlate with improved quality of life and reduced fatigue.

Causal mediation analysis will be performed in Stata for each potential mediator and each primary endpoint (39).

As STAMINA participants are randomised to their treatment arms, the mediation analysis does not need to account for confounding between treatment and either the mediator or outcome. Mediation models will be adjusted for the same covariates specified in the primary analysis approach outlined in Section 7.9 (including baseline outcome). Additionally, baseline levels of the mediators will be included in the mediator equation to account for pre-existing differences. By adjusting for observed confounders, we assume that any residual unmeasured confounding is negligible. An interaction between the mediator and treatment will be included in the mediation model to allow the mediator effect to vary across treatment arms.

As the potential mediators are continuous, the following models will be fitted in Stata, where m1 denotes the continuous mediator variable, m1baseline represents the corresponding baseline mediator level, and x1,…,xn denotes the additional covariates:

Mediate (FACT-P total score x1,…,xn) (m1 m1baseline) (treatment)
Mediate (FACT-F FS subscale x1,…,xn) (m1 m1baseline) (treatment).

The total effect of treatment on each primary outcome will be estimated, along with the direct and indirect effects (i.e. the component of the total effect mediated through m1). The proportion of the total effect attributable to the mediator will also be reported with a 97.232% confidence interval. Appropriate model assumption checks will be conducted, including the use of scatter plots of the mediator against the outcome.

Copyright © 2007 CTRU, University of Leeds. All rights reserved.

7.14.3. Dose-response analysis

If the results of the CACE analysis are uninformative, modelling of the dose-response relationship between SLI and the primary endpoints will be considered.

8. Reporting and dissemination of results

Analysis will follow the template laid out in this Statistical Analysis Plan. After analysis is complete, the results will be presented to the project teams and a full report will be written up so it can be used as part of the threaded publication plan requested by the NIHR. It is estimated that it will take six months from the final download of the MACRO database to finalisation of the statistical report. The results will also be written up into a manuscript for submission to a peer-reviewed journal. The results may also be submitted as abstracts to appropriate conferences for either poster or oral presentation. All abstracts and manuscripts will be prepared and reviewed in accordance with the publication policy in the trial protocol.

9. References

1. Ness AR WA, Hurley K, Jeffreys M, Penfold C, Pring M, et al. . Recruitment, response rates and characteristics of 5511 people enrolled in a prospective clinical cohort study: head and neck 5000. . Clin Otolaryngology 2016;41:804-9.
2. Mason MD PW, Sydes MR, et al. Final Report of the Intergroup Randomized Study of Combined Androgen-Deprivation Therapy Plus Radiotherapy Versus Androgen-Deprivation Therapy Alone in Locally Advanced Prostate Cancer. J Clin Oncol. 2015;33:2143-50.
3. James ND SM, Clarke NW, et al. Addition of docetaxel, zoledronic acid, or both to first-line long-term hormone therapy in prostate cancer (STAMPEDE): survival results from an adaptive, multiarm, multistage, platform randomised controlled trial. Lancet. 2016;387:1163-77.
4. FJ F. The impact of androgen deprivation on quality of life after radical prostatectomy for prostate carcinoma. Cancer. 2002;95:287-95.
5. K D. Quality of life in prostate cancer patients taking androgen deprivation therapy. J Am Geriatric Soc. 2006;54:85-90.
6. Cheung AS dRC, Hoermann R, Lim Joon D, Zajac JD, Grossmann M. Quality of life decrements in men with prostate cancer undergoing androgen deprivation therapy. Clin Endocrinol (Oxf). 2017;86:388-94.
7. Bourke L CT, Albertsen PC, Hamdy FC, Rosario DJ. Cardiovascular risk in androgen suppression: underappreciated, under-researched and unresolved. Heart. 2011;98:345-8.
8. Bourke L GS, Hooper R, et al. Lifestyle changes for improving disease specific quality of life in sedentary men on long-term androgen-deprivation therapy for advanced prostate cancer: a randomised controlled trial. European urology. 2014;65:865-72.
9. Gunlusoy B CY, Koskderelioglu A, et al. Cognitive Effects of Androgen Deprivation Therapy in Men With Advanced Prostate Cancer. Urology. 2017.
10. DiBlasio CJ HJ, Malcolm JB, et al. Prevalence and predictive factors for the development of de novo psychiatric illness in patients receiving androgen deprivation therapy for prostate cancer. The Canadian Journal of Urology. 2008;15:4249-56.
11. Galvao DA TD, Spry N, Joseph D, Newton RU. Combined resistance and aerobic exercise program reverses muscle loss in men undergoing androgen suppression therapy for prostate cancer without bone metastases: a randomized controlled trial. J Clin Oncol. 2010;28:340-7.
12. Segal RJ RR, Courneya KS, et al. Resistance exercise in men receiving androgen deprivation therapy for prostate cancer. J Clin Oncol. 2003;21:1653-9.
13. Segal RJ RR, Courneya KS, et al. Randomized controlled trial of resistance or aerobic exercise in men receiving radiation therapy for prostate cancer. J Clin Oncol. 2009;27:344-51.
14. Prostate Cancer: Full text guidelines. 2017.
15. Giovannucci E LY, Platz EA, Stampfer MJ, Willett WC. Risk factors for prostate cancer incidence and progression in the health professionals follow-up study. Int J Cancer. 2007;121:1571-8.

Copyright © 2007 CTRU, University of Leeds. All rights reserved.

16. McGowan EL NS, Courneya KS. Randomized controlled trial of a behavior change intervention to increase physical activity and quality of life in prostate cancer survivors. Annals of behavioral medicine : a publication of the Society of Behavioral Medicine. 2013;46:382-93.
17. Hackshaw-McGeagh LE PC, Walsh E, et al. Physical activity, alcohol consumption, BMI and smoking status before and after prostate cancer diagnosis in the ProtecT trial: opportunities for lifestyle modification. Int J Cancer. 2015;137:1509-15.
18. Bassett JC GJ, Chi AC, et al. Impact of a bladder cancer diagnosis on smoking behavior. J Clin Oncol. 2012;30:1871-8.
19. King AJ EM, Moore TH, et al. Prostate cancer and supportive care: a systematic review and qualitative synthesis of men's experiences and unmet needs. Eur J Cancer Care (Engl). 2015;24:618-34.
20. Bourke L SR, Nanton V, Crank H, Rosario DJ, Saxton JM. A qualitative study evaluating experiences of a lifestyle intervention in men with prostate cancer undergoing androgen suppression therapy. Trials. 2012;13:208.
21. Health N. Nuffield Health to expand exercise programme for cystic fibrosis patients in NHS hospitals. 2016.
22. Murray E, Treweek S, Pope C, MacFarlane A, Ballini L, Dowrick C, Finch T, Kennedy A, Mair F, O'Donnell C, Ong BN, Rapley T, Rogers A, May C. Normalisation process theory: a framework for developing, evaluating and implementing complex interventions. BMC Med. 2010 Oct 20;8:63. doi: 10.1186/1741-7015-8-63. PMID: 20961442; PMCID: PMC2978112.
23. Duda JL, Williams GC, Ntoumanis N, Daley A, Eves FF, Mutrie N, Rouse PC, Lodhia R, Blamey RV, Jolly K. Effects of a standard provision versus an autonomy supportive exercise referral programme on physical activity, quality of life and well-being indicators: a cluster randomised controlled trial. Int J Behav Nutr Phys Act. 2014 Jan 29;11:10. doi: 10.1186/1479-5868-11-10. PMID: 24475766; PMCID: PMC3931311.
24. Lafaye de Micheaux P, Liquet B, Marque S, Riou J. Power and sample size determination in clinical trials with multiple primary continuous correlated endpoints. J Biopharm Stat. 2014;24(2):378-97. doi: 10.1080/10543406.2013.860156. PMID: 24605975.
25. Bourke L, Gilbert S, Hooper R, Steed LA, Joshi M, Catto JW, Saxton JM, Rosario DJ. Lifestyle changes for improving disease-specific quality of life in sedentary men on long-term androgen-deprivation therapy for advanced prostate cancer: a randomised controlled trial. Eur Urol. 2014 May;65(5):865-72. doi: 10.1016/j.eururo.2013.09.040. Epub 2013 Oct 4. PMID: 24119318.
26. Krakauer NY, Krakauer JC. A new body shape index predicts mortality hazard independently of body mass index. PLoS One. 2012;7(7):e39504. doi: 10.1371/journal.pone.0039504. Epub 2012 Jul 18. PMID: 22815707; PMCID: PMC3399847.
27. Carpenter, J. and Smuk, M., 2021. Missing data: A statistical framework for practice. Biometrical Journal, 63(5), pp.915-947.
28. Jakobsen, J.C., Gluud, C., Wetterslev, J. et al. When and how should multiple imputation be used for handling missing data in randomised clinical trials – a practical guide with flowcharts. BMC Med Res Methodol 17, 162 (2017).
29. Fiero, M., Hsu, C. and Bell, M., 2017. A pattern-mixture model approach for handling missing continuous outcome data in longitudinal cluster randomized trials. Statistics in Medicine, 36(26), pp.4094-4105.
30. ACPICR, (Association of Chartered Physiotherapists in Cardiovascular Rehabilitation) Healthcare Professionals publications [Available from: https://www.acpicr.com/publications/healthcare-professionals/ Accessed 01/04/19.
31. Connell AM. Employing complier average causal effect analytic methods to examine effects of randomized encouragement trials. Am J Drug Alcohol Abuse. 2009;35(4):253-9. doi: 10.1080/00952990903005882. PMID: 20180678; PMCID: PMC2845534.
32. Schulz, K.F., Altman, D.G., Moher, D. *et al.* CONSORT 2010 Statement: updated guidelines for reporting parallel group randomised trials. *BMC Med* **8**, 18 (2010). https://doi.org/10.1186/1741-7015-8-18
33. Flight L, Allison A, Dimairo M, Lee E, Mandefield L, Walters S. *Recommendations for the analysis of individually randomised controlled trials with clustering in one arm – a case of continuous outcomes.* BMC Medical Research Methodology. 2016;16(1).
34. Clarke P. *When can group level clustering be ignored? Multilevel models versus single-level models with sparse data. Journal of Epidemiology & Community Health*. 2008;62(8):752-758.

Copyright © 2007 CTRU, University of Leeds. All rights reserved.

35. Connell A. Employing Complier Average Causal Effect Analytic Methods to Examine Effects of Randomized Encouragement Trials. The American Journal of Drug and Alcohol Abuse. 2009;35(4):253-259.
36. Jo, B., & Muthen, B. (2003). Longitudinal studies with intervention and noncompliance: Estimation of causal effects in growth mixture modelling. In N. Duan & S. Reise (Eds.), Multilevel modelling: Methodological Advances, issues, and applications, multivariate applications book series(pp. 112–139). Mahwah, NJ: Lawrence Erlbaum Associates Inc
37. Huang S, Cordova D, Estrada Y, Brincks A, Asfour L, Prado G. An Application of the Complier Average Causal Effect Analysis to Examine the Effects of a Family Intervention in Reducing Illicit Drug Use among High-Risk Hispanic Adolescents. Family Process. 2014;53(2):336-347.
38. Wang R, Ware JH. Detecting moderator effects using subgroup analyses. Prev Sci. 2013 Apr;14(2):111-20. doi: 10.1007/s11121-011-0221-x. PMID: 21562742; PMCID: PMC3193873.
39. StataCorp. 2023. Stata 18 Causal Inference and Treatment-Effects Estimation Reference Manual. College Station, TX: Stata Press. https://www.stata.com/manuals/causal.pdf

10. Appendices

10.1. Appendix 1 – Key data items

Database	Form	Description	Field Name
Main	F07 (Randomisation)	Patient date of birth<br><br>Stratification factors<br><br><br><br><br>Randomisation allocation<br><br>Date of randomisation	DOBF07P1<br><br>AgeF07P1<br>PatADTF07P1<br>ChemoF07P1<br>RadioF07P1<br><br>AllocF07P1<br><br>RandDateF07P1
	F14 (Notification of death)	Date of death	DateDeathF14P1
	F15 (Clinical withdrawal)	Date of withdrawal<br><br>Reason for discontinuation<br><br>Time point of withdrawal	WithDateF15P1<br><br>DiscReasF15P1<br><br>WithTimeF15P1
	F16 (Participant withdrawal request)	Date of withdrawal<br><br>Has the participant withdrawn from the STAMINA lifestyle intervention?<br><br>Time of withdrawal<br><br>Has the participant withdrawn from follow-up appointments?<br><br>Has the participant withdrawn from questionnaire completion?<br><br>Has the participant withdrawn from the collection of data from medical records?<br><br>Has a reason for withdrawal been	WithDateF16P1<br><br>PartWithF16P1<br><br>TimeWithF16P1<br><br>FUPWithF16P1<br><br>QuestWithF16P1<br><br>RecWithF16P1<br><br>WithReasYNF16P1

Copyright © 2007 CTRU, University of Leeds. All rights reserved.

		given?<br><br>Details of withdrawal	WithReasF16P1
	F24 (Exercise Record (Week 1 - 12))	Clinical Exercise Specialist<br><br>Mode of delivery<br><br>Date of session	NHCESIDF24P1<br>DelivAF24P1<br>SessDateF24P1
	F27 (Exercise Record (Week 13 - 19))		NHCESIDF27P1<br>DelivAF27P1<br>SessDateF27P1
	F31 (Exercise Record (Week 19 – Month 6))		NHCESIDF31P1<br>DelivAF31P1<br>SessDateF31P1
	F31 (Exercise Record (Week 19 – Month 6))		NHCESIDF33P1<br>DelivAF33P1<br>SessDateF33P1
	F35 (Exercise Record (Week 32 – Month 9))		NHCESIDF35P1<br>DelivAF35P1<br>SessDateF35P1
	F39 (Exercise Record (Week 45 – Month 12))		NHCESIDF39P1<br>DelivAF39P1<br>SessDateF39P1
REDCap*	Participant questionnaire pack (Baseline and follow up)	All FACIT fields<br><br>Date of FACT completion	FACT1 – FACT52<br><br>CompDate

*These constitute key data but as they are entered directly into RedCap by participants they cannot be checked as correct.

10.2. Appendix 2 - Internal pilot and progression criteria

10.2.1. Objectives

The internal pilot progression criteria, as defined in the PGfAR application, were set to assess three important aspects of trial delivery:

- Recruitment rates per site per month
- Follow-up rates at 3 months
- Intervention adherence at 12 weeks

The trial design and implementation were updated from those outlined in the PGfAR application to reflect results from the earlier work packages and context/setting adaptations arising from the COVID-19 pandemic. The number of sites required for recruitment decreased and consequently the number of participants required per site increased. The internal pilot progression criteria were revisited and revised as shown in Table 1. Furthermore, the progression criterion relating to intervention adherence was defined in more detail, to clarify its assessment.

Table 1: Updated progression criteria

Decision Criterion	Go (green)	Modify (amber)	Stop (red)
**Recruitment** [1,2,3]<br>Average number of participants randomised per site per month	≥4	4 < and ≥ 2	<2
**Follow-up at 3 months**[4]<br>% participants completing 3m outcomes	≥75%	50-74%	<50%

Copyright © 2007 CTRU, University of Leeds. All rights reserved.

**12 weeks (patient) intervention adherence**<br>% participants completing 75% scheduled SLI exercise sessions within 18 weeks of randomisation	≥75%	50-74%	<50%

[1] Data from August 2022 onwards was used for analysis of this criterion to allow for a sufficient number of sites to be open and to ensure the trial has reached a steady rate of recruitment.
[2] This target is set at a gym level. Therefore, for sites with 2 gyms the criteria are: Green ≥8, Amber 8 < and ≥ 4, and Red <4.
[3] Based on 12 recruiting sites.
[4] FACT-P and FACT-F only.

10.2.2. Analysis

Descriptive statistics were used to evaluate the progression criteria; no formal statistical testing was performed.

10.2.3. Results

Table 2: Number of participants available for assessment in the internal pilot

Decision Criterion	Number of participants with available data
**Recruitment[1,2]**<br>Average number of participants randomised per site per month	267 (between 9 and 14 NHS sites)<br>(400 recruited overall from up to 14 NHS sites)
**Follow-up at 3 months[3]**<br>% participants completing 3m outcomes	178 (from 8 NHS sites)
**12 weeks (patient) intervention adherence[5]**<br>% participants completing 75%<br>scheduled SLI exercise sessions within 18 weeks of randomisation	115 (from 8 NHS sites)

[1]This target is set at a gym level.
[2] Based on participants recruited between August 2022 and end of January 2023.
[3] Based on participants recruited between January 2022 and end of August 2022. Allows for September/October/November follow-up period and a 2-month chase period in December/January.
[5] Based on participants recruited up to 9th September 2022. Allows for October/November/December follow-up period and a 1-month chase period in January 2023.

Copyright © 2007 CTRU, University of Leeds. All rights reserved.

10.2.4. Recruitment

Table 3: Monthly recruitment

Site No.	Site Code	Site Name	Date Opened	Date Recruiting	No. of Gyms Open	Date Stopped Referrals	Days Between NHS Green Light and NH Gym Green Light	Total Patients Registered	Total Patients Randomised	Jan-22	Feb-22	Mar-22	Apr-22	May-22	Jun-22	Jul-22	Aug-22	Sep-22	Oct-22	Nov-22	Dec-22	Jan-23
1	50147	University Hospitals Plymouth NHS Trust	20/01/2022	09/02/2022	2		20	117	107		8	11	9	13	7	6	4	11	13	10	8	7
2	10852	University Hospitals Coventry & Warwickshire NHS Trust	11/02/2022	11/02/2022	2		0	61	50		0	2	1	2	3	7	3	5	7	8	3	3
3	14035	Chesterfield Royal Hospital NHS Foundation Trust	11/02/2022	11/02/2022	1	16/11/2022	0	49	48		0	0	9	7	6	6	4	5	3	4	3	1
4	13095	Nottingham University Hospitals NHS Trust	11/03/2022	25/04/2022	1		45	36	29				0	1	3	2	3	5	3	5	4	3
5	00093/00062	Sheffield Teaching Hospitals NHS Trust (RHH & WPH)	06/04/2022	06/04/2022	1		0	73	62				0	1	4	7	15	6	11	5	5	5
6	10894	Surrey and Sussex Healthcare NHS Trust	20/04/2022	26/05/2022	2		36	56	46					5	1	3	6	9	7	7	3	3
7	00046	Liverpool University Hospitals NHS Foundation Trust	22/04/2022	22/04/2022	1		0	16	13				0	0	3	2	2	3	1	0	0	2
8	00168	University Hospitals of Derby and Burton NHS Foundation Trust	15/06/2022	13/09/2022	1		90	9	8									2	5	0	0	1
9	17377	Cambridge University Hospital NHS Foundation Trust	25/05/2022	30/06/2022	1		36	29	26						0	4	8	3	0	4	4	0
10	19287	Yeovil District Hospital NHS Foundation Trust	08/07/2022	06/09/2022	1		60	9	8									1	0	1	2	3
11	11402	Stockport NHS Foundation Trust	19/07/2022	25/08/2022	1		37	17	16								0	0	0	6	5	3
12	00075/18978	Bradford Teaching Hospitals NHS Foundation Trust (BRI & SLH)	02/11/2022	06/12/2022	1		34	0	0												0	0
13	11055	Gloucestershire Hospitals NHS Foundation Trust	25/11/2022	09/12/2022	1		14	6	4												0	4
14	50271	Hull University Teaching Hospitals NHS Trust	05/01/2023		1			3	0													
15	20170	Clatterbridge Cancer Centre NHS Foundation Trust	13/01/2023	13/01/2023	1		0	1	0													0
			15	13				484	418	0	8	13	19	29	27	37	45	50	50	50	37	35
								Total OUC	187	0	8	21	40	69	96	133	178	228	278	328	365	400
								Total SU + OUC	231													
									Monthly target:	*0*	*8*	*13*	*19*	*29*	*27*	*37*	*44*	*48*	*60*	*60*	*60*	*60*
									Cumulative target:	*0*	*8*	*21*	*40*	*69*	*96*	*133*	*177*	*225*	*285*	*345*	*405*	*465*

Table 4: Recruitment rates (between August-22 and January-23) [1]

Site No.	Site Code	Site Name	Total randomised	Number of months open (per single gym)	Recruitment rate
1	50147	University Hospitals Plymouth NHS Trust	53	12	4.4
2	10852	University Hospitals Coventry & Warwickshire NHS Trust	29	12	2.4
3	14035	Chesterfield Royal Hospital NHS Foundation Trust	20	6	3.3
4	13095	Nottingham University Hospitals NHS Trust	23	6	3.8
5	00093/00062	Sheffield Teaching Hospitals NHS Trust (RHH & WPH)	47	6	7.8
6	10894	Surrey and Sussex Healthcare NHS Trust	35	12	2.9
7	00046	Liverpool University Hospitals NHS Foundation Trust	8	6	1.3
8	00168	University Hospitals of Derby and Burton NHS Foundation Trust	8	5	1.6
9	17377	Cambridge University Hospital NHS Foundation Trust	19	6	3.2
10	19287	Yeovil District Hospital NHS Foundation Trust	7	5	1.4
11	11402	Stockport NHS Foundation Trust	14	6	2.3
12	00075/18978	Bradford Teaching Hospitals NHS Foundation Trust (BRI & SLH)	0	2	0.0
13	11055	Gloucestershire Hospitals NHS Foundation Trust	4	2	2.0
14	50271	Hull University Teaching Hospitals NHS Trust	0	-	-
15	20170	Clatterbridge Cancer Centre NHS Foundation Trust	0	1	0.0
		**Total**	**267**	**87**	**3.1**

[1] Recruitment is being assessed over a 6-month period hence the total randomised is the number recruited during this period. The number of months open relates to the number of months during this period that sites were open. Three sites have 2 gyms, these sites were open for the full 6-month period however as their target recruitment rate is double that of the other sites, the calculation assumes they have been open for 12 months to allow assessment of the overall recruitment rate

Copyright © 2007 CTRU, University of Leeds. All rights reserved.

across all sites.

Between August-2022 and January-2023 the overall number of men recruited per site per month across all sites is **3.1** which falls into the **amber range.**

Excluding Bradford, Gloucestershire, Hull and Clatterbridge (to ensure sites have had time to recruit) results in a recruitment rate of **3.2** men per site per month which still falls into the **amber range.**

Figure 2: Recruitment graph

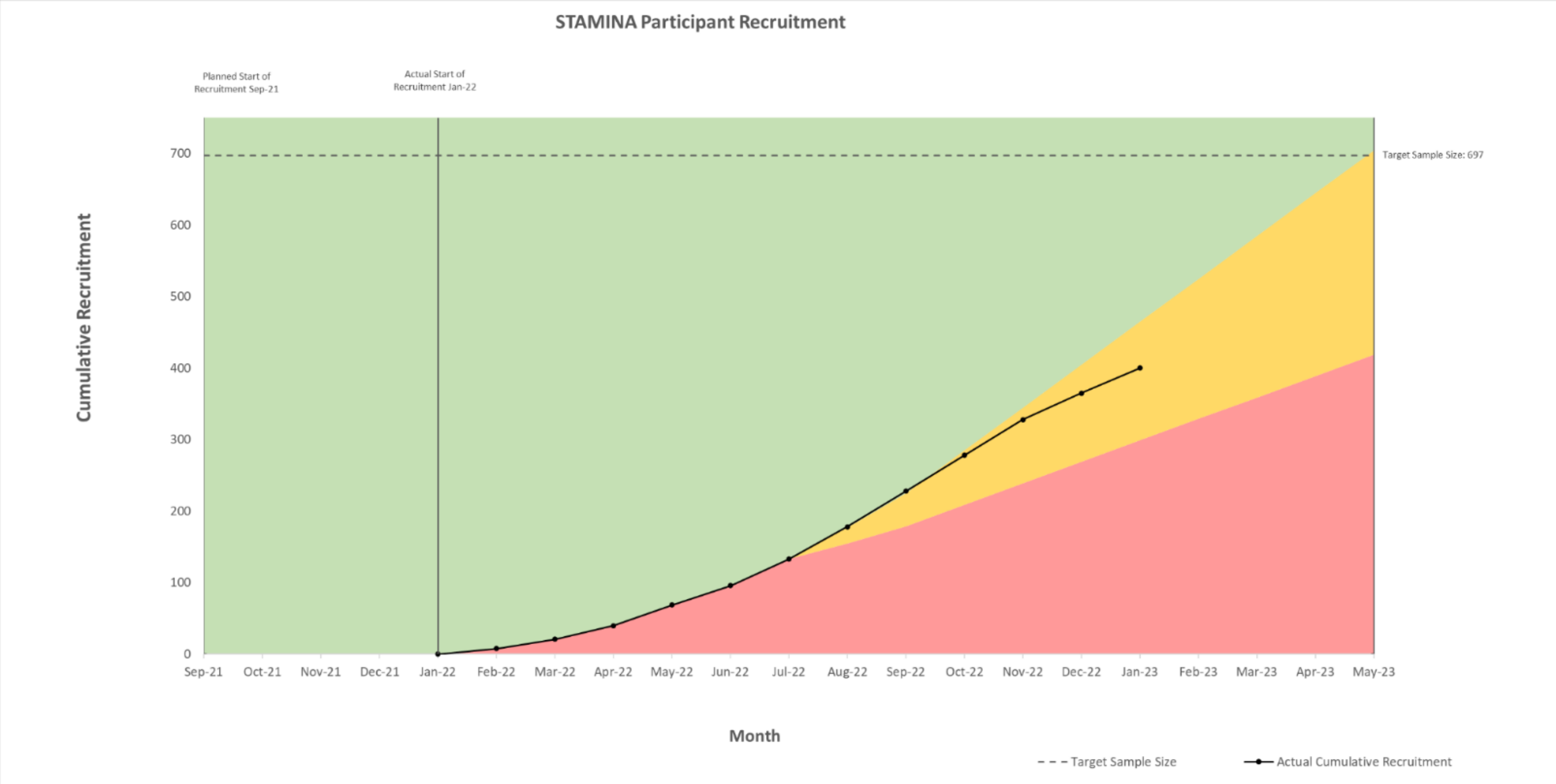

Copyright © 2007 CTRU, University of Leeds. All rights reserved.

10.2.5. Follow-up at 3 months

There were 178 participants randomised between January-22 and August-22 and all of them completed their baseline FACIT questionnaire.

Table 5: FACIT completion at three-months

	STAMINA Lifestyle Intervention	Optimised Usual Care	Total
Yes	90 (90.0%)	61 (78.2%)	151 (84.8%)
No	10 (10.0%)	17 (21.8%)	27 (15.2%)
Total	100 (100%)	78 (100%)	178 (100%)

One participant in the Optimised Usual Care arm did not complete the FACIT due to withdrawal. This participant stated that they had 'Changed their mind about being involved in the trial'. None of the remaining 26 participants had died prior to reaching their three-month follow-up time point. Reasons for non-completion are not available for these 26 participants.
Three-month FACIT completion is **84.8%** which falls into the **green range.**

Table 6: FACIT completion at three-months by site

	Completed	Not completed	Total
University Hospitals Plymouth NHS Trust	50 (86.2%)	8 (13.8%)	58 (100%)
University Hospitals Coventry & Warwickshire NHS Trust	16 (88.9%)	2 (11.1%)	18 (100%)
Chesterfield Royal Hospital NHS Foundation Trust	26 (81.3%)	6 (18.8%)	32 (100%)
Nottingham University Hospital NHS Trust	8 (88.9%)	1 (11.1%)	9 (100%)
Sheffield Teaching Hospitals NHS Trust	23 (85.2%)	4 (14.8%)	27 (100%)
Surrey and Sussex Healthcare NHS Trust	12 (80.0%)	3 (20.0%)	15 (100%)
Liverpool University Hospitals NHS Foundation Trust	7 (100%)	0 (0.0%)	7 (100%)
Cambridge University Hospitals NHS Trust	9 (75.0%)	3 (25.0%)	12 (100%)

Copyright © 2007 CTRU, University of Leeds. All rights reserved.

Table 7: FACIT completion at three-months by site (non-completers)

	STAMINA Lifestyle Intervention	Optimised Usual Care	Total
University Hospitals Plymouth NHS Trust	2 (25.0%)	6 (75.0%)	8 (100%)
University Hospitals Coventry & Warwickshire NHS Trust	0 (0.0%)	2 (100%)	2 (100%)
Chesterfield Royal Hospital NHS Foundation Trust	4 (66.7%)	2 (33.3%)	6 (100%)
Nottingham University Hospital NHS Trust	0 (0.0%)	1 (100%)	1 (100%)
Sheffield Teaching Hospitals NHS Trust	2 (50.0%)	2 (50.0%)	4 (100%)
Surrey and Sussex Healthcare NHS Trust	1 (33.3%)	2 (66.7%)	3 (100%)
Cambridge University Hospitals NHS Trust	1 (33.3%)	2 (66.7%)	3 (100%)

Table 8: FACIT completion at three-months by site (completers)

	STAMINA Lifestyle Intervention	Optimised Usual Care	Total
University Hospitals Plymouth NHS Trust	35 (70.0%)	15 (30.0%)	50 (100%)
University Hospitals Coventry & Warwickshire NHS Trust	7 (43.8%)	9 (56.3%)	16 (100%)
Chesterfield Royal Hospital NHS Foundation Trust	12 (46.2%)	14 (53.8%)	26 (100%)
Nottingham University Hospital NHS Trust	5 (62.5%)	3 (37.5%)	8 (100%)
Sheffield Teaching Hospitals NHS Trust	13 (56.5%)	10 (43.5%)	23 (100%)
Surrey and Sussex Healthcare NHS Trust	6 (50.0%)	6 (50.0%)	12 (100%)
Liverpool University Hospitals NHS Foundation Trust	7 (100%)	0 (0.0%)	7 (100%)
Cambridge University Hospitals NHS Trust	5 (55.6%)	4 (44.4%)	9 (100%)

Copyright © 2007 CTRU, University of Leeds. All rights reserved.

Table 9: Age of completers and non-completers by arm

	Completers			Non-completers		
	**STAMINA Lifestyle Intervention**	**Optimised Usual Care**	**Total**	**STAMINA Lifestyle Intervention**	**Optimised Usual Care**	**Total**
Less than 70 years	36 (40.0%)	23 (37.7%)	59 (39.1%)	4 (40.0%)	5 (29.4%)	9 (33.3%)
Greater than or equal to 70 years	54 (60.0%)	38 (62.3%)	92 (60.9%)	6 (60.0%)	12 (70.6%)	18 (66.7%)
Total	90 (100%)	61 (100%)	151 (100%)	10 (100%)	17 (100%)	27 (100%)

Table 10: Length of time on ADT for completers and non-completers by arm

	Completers			Non-completers		
	**STAMINA Lifestyle Intervention**	**Optimised Usual Care**	**Total**	**STAMINA Lifestyle Intervention**	**Optimised Usual Care**	**Total**
Less than or equal to 12 weeks	46 (51.1%)	30 (49.2%)	76 (50.3%)	5 (50.0%)	8 (47.1%)	13 (48.1%)
Greater than 12 weeks	44 (48.9%)	31 (50.8%)	75 (49.7%)	5 (50.0%)	9 (52.9%)	14 (51.9%)
Total	90 (100%)	61 (100%)	151 (100%)	10 (100%)	17 (100%)	27 (100%)

Copyright © 2007 CTRU, University of Leeds. All rights reserved.

Table 11: Receipt of chemotherapy or ARI for completers and non-completers by arm

	Completers			Non-completers		
	STAMINA Lifestyle Intervention	Optimised Usual Care	Total	STAMINA Lifestyle Intervention	Optimised Usual Care	Total
Yes	28 (31.1%)	17 (27.9%)	45 (29.8%)	4 (40.0%)	4 (23.5%)	8 (29.6%)
No	62 (68.9%)	44 (72.1%)	106 (70.2%)	6 (60.0%)	13 (76.5%)	19 (70.4%)
Total	90 (100%)	61 (100%)	151 (100%)	10 (100%)	17 (100%)	27 (100%)

Table 12: Receipt of radiotherapy for completers and non-completers by arm

	Completers			Non-completers		
	STAMINA Lifestyle Intervention	Optimised Usual Care	Total	STAMINA Lifestyle Intervention	Optimised Usual Care	Total
Yes	4 (4.4%)	3 (4.9%)	7 (4.6%)	0 (0.0%)	1 (5.9%)	1 (3.7%)
No	86 (95.6%)	58 (95.1%)	144 (95.4%)	10 (100.0%)	16 (94.1%)	26 (96.3%)
Total	90 (100%)	61 (100%)	151 (100%)	10 (100%)	17 (100%)	27 (100%)

Copyright © 2007 CTRU, University of Leeds. All rights reserved.

10.2.6. 12 weeks (participant) intervention adherence

There were 115 participants randomised to the STAMINA Lifestyle Intervention up to and including 9th September 2022.

We have withdrawal forms to show three participants withdrew from the SLI within 18 weeks of randomisation (one before starting the SLI and two after starting the SLI). Reasons for withdrawal were provided as 'Does not like the STAMINA Intervention x2', 'Changed their mind about being involved in the trial x1' and 'Has to travel too far to attend gym x1'.

Table 13: SLI Scheduled Sessions (categorical)

Number of sessions scheduled	Number of participants
0	6 (5.2%)
5	1 (0.9%)
6	1 (0.9%)
8	1 (0.9%)
10	1 (0.9%)
11	1 (0.9%)
13	1 (0.9%)
14	1 (0.9%)
16	3 (2.6%)
17	2 (1.7%)
18	6 (5.2%)
19	1 (0.9%)
20	10 (8.7%)
22	10 (8.7%)
23	9 (7.8%)
24	61 (53.0%)
Total	115 (100%)

Copyright © 2007 CTRU, University of Leeds. All rights reserved.

Figure 3: SLI Scheduled sessions

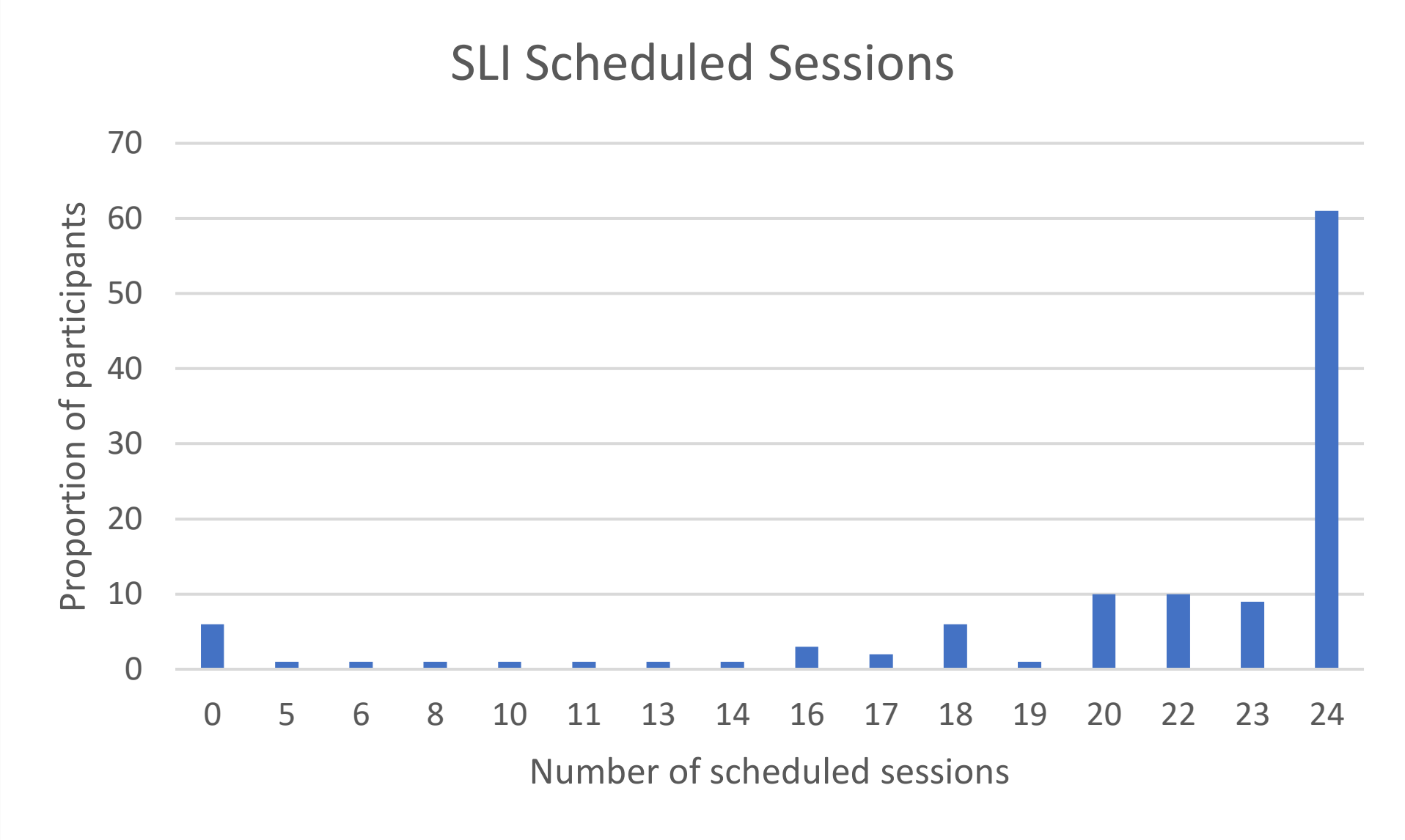

Table 14: SLI Scheduled sessions (summary statistics)

Number of scheduled sessions	
Mean (s.d.)	20.6 (6.2)
Median (range)	24 (0, 24)
Missing	0
N	115

Table 15: Reasons for non-scheduling of SLI sessions

**Reasons for non-scheduling**
Reason unknown
CES Annual leave / availability
Patient illness
Planned holiday
Work commitments
Withdrawal from SLI intervention
Travel distance to NH
Festive week
House move
Hospital appointment / admission
Patient request for rest day
Transition to new medication
Patient time constraints
Radiotherapy
Operation

Copyright © 2007 CTRU, University of Leeds. All rights reserved.

Table 16: SLI Scheduled Sessions (summary statistics) by site

Number of scheduled SLI sessions		
University Hospitals Plymouth NHS Trust	Mean (s.d.)	22.0 (3.9)
	Median (range)	24 (5, 24)
	Missing	0
	N	44
University Hospitals Coventry & Warwickshire NHS Trust	Mean (s.d.)	20.6 (5.5)
	Median (range)	23 (8, 24)
	Missing	0
	N	8
Chesterfield Royal Hospital NHS Foundation Trust	Mean (s.d.)	18.0 (6.6)
	Median (range)	20 (0, 24)
	Missing	0
	N	18
Nottingham University Hospital NHS Trust	Mean (s.d.)	23.9 (0.4)
	Median (range)	24 (23, 24)
	Missing	0
	N	7
Sheffield Teaching Hospitals NHS Trust	Mean (s.d.)	18.1 (9.3)
	Median (range)	23 (0, 24)
	Missing	0
	N	17
Surrey and Sussex Healthcare NHS Trust	Mean (s.d.)	19.5 (8.2)
	Median (range)	23 (0, 24)
	Missing	0
	N	8
Liverpool University Hospitals NHS Foundation Trust	Mean (s.d.)	23.0 (1.5)
	Median (range)	24 (20, 24)
	Missing	0
	N	7
Cambridge University Hospitals NHS Trust	Mean (s.d.)	20.0 (9.8)
	Median (range)	24 (0, 24)
	Missing	0
	N	6

Copyright © 2007 CTRU, University of Leeds. All rights reserved.

Table 17: SLI Session Attendance (categorical)

Number of sessions attended	Number of participants attending	Number of participants attending (cumulative)
0	6 (5.2%)	6 (5.2%)
1	1 (0.9%)	7 (6.1%)
2	5 (4.3%)	12 (10.4%)
3	2 (1.7%)	14 (12.2%)
4	1 (0.9%)	15 (13.0%)
5	1 (0.9%)	16 (13.9%)
6	1 (0.9%)	17 (14.8%)
8	1 (0.9%)	18 (15.7%)
9	1 (0.9%)	19 (16.5%)
10	2 (1.7%)	21 (18.3%)
11	1 (0.9%)	22 (19.1%)
12	1 (0.9%)	23 (20.0%)
13	5 (4.3%)	28 (24.3%)
14	1 (0.9%)	29 (25.2%)
15	4 (3.5%)	33 (28.7%)
16	8 (7.0%)	41 (35.7%)
17	6 (5.2%)	47 (40.9%)
18	8 (7.0%)	55 (47.8%)
19	10 (8.7%)	65 (56.5%)
20	7 (6.1%)	72 (62.6%)
21	5 (4.3%)	77 (67.0%)
22	14 (12.2%)	91 (79.1%)
23	8 (7.0%)	99 (86.1%)
24	16 (13.9%)	115 (100.0%)
Total	115 (100%)	-

Figure 4: SLI Session attendance

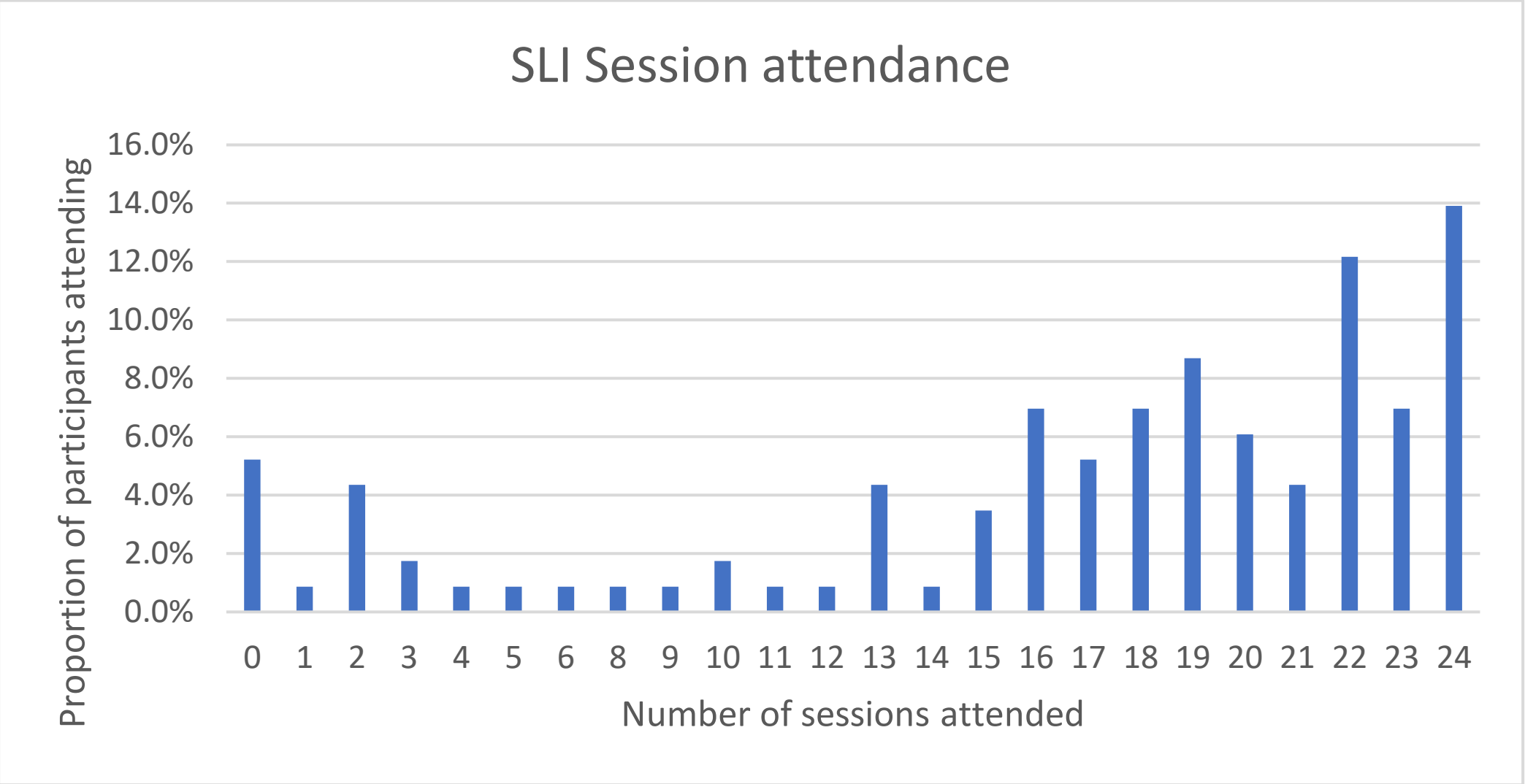

Copyright © 2007 CTRU, University of Leeds. All rights reserved.

Table 18: SLI Session Attendance (summary statistics)

Number of sessions attended	
Mean (s.d.)	16.7 (7.2)
Median (range)	19 (0, 24)
Missing	0
N	115

Table 19: Reasons for non-attendance at SLI sessions

Reasons for non-attendance
Ankle injury
Appointment clash
Awaiting covid test results
Away with work
Caring responsibilities
Covid jab
Holiday & Recovery from broken ribs
Isolating ahead of an operation
Motorbike broke down on route
Neck and shoulder ache
Patient did not attend over festive period
Patient fatigue
Patient holiday
Patient recovering from broken ribs
Patient tested positive for covid
Patient unwell
Patient's previous appointment overan so session missed
Radiotherapy treatment
Reason unknown
Recovery from operation
Scheduled operation
Transition to new medication
Work commitments

Table 20: Proportion of participants who have attended ≥75% of scheduled SLI sessions

Number of participants being assessed	Number of participants who have attended ≥75% of SLI sessions	Proportion of participants who have attended ≥75% of SLI sessions
115	83	72.2%

Intervention adherence is **72.2%** which falls into the **amber range.**

Copyright © 2007 CTRU, University of Leeds. All rights reserved.

Table 21: Proportion of participants who have attended ≥75% of scheduled SLI sessions by site

Site	Number of participants being assessed	Number of participants who have attended ≥75% of scheduled SLI sessions	Proportion of participants who have attended ≥75% of scheduled SLI sessions
University Hospitals Plymouth NHS Trust	44	34	72.2%
University Hospitals Coventry & Warwickshire NHS Trust	8	5	62.5%
Chesterfield Royal Hospital NHS Foundation Trust	18	13	72.2%
Nottingham University Hospital NHS Trust	7	6	85.7%
Sheffield Teaching Hospitals NHS Trust	17	10	58.8%
Surrey and Sussex Healthcare NHS Trust	8	5	62.5%
Liverpool University Hospitals NHS Foundation Trust	7	6	85.7%
Cambridge University Hospitals NHS Trust	6	4	66.7%

Copyright © 2007 CTRU, University of Leeds. All rights reserved.

Table 22: SLI Session Attendance (summary statistics) by site

Number of sessions attended		
University Hospitals Plymouth NHS Trust	Mean (s.d.)	17.7 (6.1)
	Median (range)	19 (1, 24)
	Missing	0
	N	44
University Hospitals Coventry & Warwickshire NHS Trust	Mean (s.d.)	15.8 (5.7)
	Median (range)	16 (6, 24)
	Missing	0
	N	8
Chesterfield Royal Hospital NHS Foundation Trust	Mean (s.d.)	13.8 (7.9)
	Median (range)	17 (0, 23)
	Missing	0
	N	18
Nottingham University Hospital NHS Trust	Mean (s.d.)	21.6 (2.7)
	Median (range)	23 (17, 24)
	Missing	0
	N	7
Sheffield Teaching Hospitals NHS Trust	Mean (s.d.)	15.5 (9.4)
	Median (range)	20 (0, 24)
	Missing	0
	N	17
Surrey and Sussex Healthcare NHS Trust	Mean (s.d.)	15.0 (9.5)
	Median (range)	18 (0, 24)
	Missing	0
	N	8
Liverpool University Hospitals NHS Foundation Trust	Mean (s.d.)	19.1 (2.7)
	Median (range)	20 (15, 22)
	Missing	0
	N	7
Cambridge University Hospitals NHS Trust	Mean (s.d.)	16.0 (9.9)
	Median (range)	20 (0, 24)
	Missing	0
	N	6

Table 23: Time from randomisation to first session attendance (in days)

Time from randomisation to first session attendance (in days)	
Mean (s.d.)	33.8 (17.8)
Median (range)	29 (9, 109)
Missing	0
N	109

Copyright © 2007 CTRU, University of Leeds. All rights reserved.

Table 24: Time from randomisation to first session attendance (in days) by site

Time from randomisation to first attendance in days		
University Hospitals Plymouth NHS Trust	Mean (s.d.)	30.8 (18.2)
	Median (range)	28 (9, 98)
	Missing	0
	N	44
University Hospitals Coventry & Warwickshire NHS Trust	Mean (s.d.)	33.0 (9.4)
	Median (range)	32 (21, 47)
	Missing	0
	N	8
Chesterfield Royal Hospital NHS Foundation Trust	Mean (s.d.)	49.6 (23.0)
	Median (range)	56 (15, 109)
	Missing	0
	N	17
Nottingham University Hospital NHS Trust	Mean (s.d.)	25.1 (9.7)
	Median (range)	22 (14, 41)
	Missing	0
	N	7
Sheffield Teaching Hospitals NHS Trust	Mean (s.d.)	35.8 (15.7)
	Median (range)	32 (11, 77)
	Missing	0
	N	14
Surrey and Sussex Healthcare NHS Trust	Mean (s.d.)	29.1 (12.4)
	Median (range)	25 (19, 49)
	Missing	0
	N	7
Liverpool University Hospitals NHS Foundation Trust	Mean (s.d.)	30.9 (6.4)
	Median (range)	31 (24, 39)
	Missing	0
	N	7
Cambridge University Hospitals NHS Trust	Mean (s.d.)	24.2 (4.7)
	Median (range)	22 (20, 32)
	Missing	0
	N	5

Table 25: Time from randomisation to completion of 18th session (for men who reached this point) in weeks

Time from randomisation to completion of 18th session in weeks	
Mean (s.d.)	14.1 (2.1)
Median (range)	14 (10, 18)
Missing	0
N	68

Copyright © 2007 CTRU, University of Leeds. All rights reserved.

Table 26: Time from randomisation to completion of 18th session (for men who reached this point) in weeks by site

Time between randomisation to 18th session (for men who reached this point) in weeks		
University Hospitals Plymouth NHS Trust	Mean (s.d.)	13.7 (2.3)
	Median (range)	14 (10, 18)
	Missing	0
	N	28
University Hospitals Coventry & Warwickshire NHS Trust	Mean (s.d.)	13.8 (1.6)
	Median (range)	15 (12, 15)
	Missing	0
	N	3
Chesterfield Royal Hospital NHS Foundation Trust	Mean (s.d.)	15.4 (2.6)
	Median (range)	15 (12, 18)
	Missing	0
	N	8
Nottingham University Hospital NHS Trust	Mean (s.d.)	13.6 (2.7)
	Median (range)	13 (11, 17)
	Missing	0
	N	6
Sheffield Teaching Hospitals NHS Trust	Mean (s.d.)	15.1 (1.0)
	Median (range)	15 (14, 17)
	Missing	0
	N	10
Surrey and Sussex Healthcare NHS Trust	Mean (s.d.)	13.4 (2.0)
	Median (range)	13 (12, 15)
	Missing	0
	N	4
Liverpool University Hospitals NHS Foundation Trust	Mean (s.d.)	14.2 (0.9)
	Median (range)	14 (13, 15)
	Missing	0
	N	5
Cambridge University Hospitals NHS Trust	Mean (s.d.)	13.3 (1.9)
	Median (range)	13 (12, 16)
	Missing	0
	N	4

Copyright © 2007 CTRU, University of Leeds. All rights reserved.

11. Approval of Analysis Plan

Clinical Trials Research Unit (CTRU)

The following Final analysis plan (Stage 1), February 2025, for the STAMINA trial has been approved by the following personnel. Any signed amendments to the plan will be filed with this document.

Trial Statistician: ____

Print Name: ____

Date: ____

Supervising Statistician: ____

Print Name: ____

Date: ____

CTRU Scientific Lead: ____

Print Name: ____

Date: ____

Senior Trial Co-ordinator/Manager: ____

Print Name: ____

Date: ____

Data Manager / Senior Data Manager: ____

Print Name: ____

Date: ____

CTRU Project Delivery Lead: ____

Print Name: ____

Date: ____

Chief Investigator: ____

Copyright © 2007 CTRU, University of Leeds. All rights reserved.

Print Name: ____

Date: ____

Additional information:

Copyright © 2007 CTRU, University of Leeds. All rights reserved.
